# Distinct transcriptome and traits of freshly dispersed *Pseudomonas aeruginosa* cells

**DOI:** 10.1128/msphere.00884-24

**Published:** 2024-11-27

**Authors:** Manmohit Kalia, Karin Sauer

**Affiliations:** 1 Department of Biological Sciences, Binghamton University, Binghamton, New York, USA; 2 Binghamton Biofilm Research Center, Binghamton University, Binghamton, New York, USA; University of Galway, Galway, Ireland

**Keywords:** RNA seq, CTC stain, pyoverdine, pyocyanin, macrophage, immune evasion, secretion system

## Abstract

Bacteria assume two distinct lifestyles: the planktonic and biofilm modes of growth. Additionally, dispersion has emerged as a third phenotype, accompanied by the distinct phenotypes and the unique expression of >600 genes. Here, we asked whether the distinct phenotype of dispersed cells is already apparent within minutes of egressing from the biofilm. We used RNA-seq to show that the physiology of freshly dispersed cells from *Pseudomonas aeruginosa* biofilms is highly different from those of planktonic and biofilm cells, apparent by dispersed cells uniquely expressing 194 genes. Unique and differentially expressed genes relative to planktonic or biofilm cells include genes associated with type IV pili, pyoverdine, type III and type VI secretion systems, and antibiotic resistance that are downregulated in dispersed cells, whereas the transcript abundance of genes involved in swimming motility, Hxc type II secretion system and various other virulence factors, and metabolic and energy-generating pathways are increased, indicative of dispersion coinciding with an awakening and re-energizing of dispersed cells, and a switch in virulence, further apparent by freshly dispersed cells significantly subverting engulfment by macrophages. The findings suggest that dispersed cells display a distinct phenotype within minutes of egressing from the biofilm, with freshly dispersed cells already capable of efficiently evading phagocytosis.

**IMPORTANCE:**

Dispersion is considered a transitionary phenotype, enabling bacteria to switch between the communal, biofilm lifestyle, where cells share resources and are protected from harmful conditions to the planktonic state. Here, we demonstrate that within minutes of leaving the biofilm, dispersed cells express genes and display phenotypic traits that are distinct from biofilms and planktonic cells. Our findings suggest that dispersed cells quickly adapt to a less structured and protected but more nutrient-rich environment, with this trade-off in environment coinciding with an awakening and a switch in virulence, specifically a switch from directly intoxicating host cells and potential competitors toward more broadly active virulence factors and strategies of evasion. To our knowledge, this is the first report of dispersed cells’ distinct (trade-off) phenotype and their enhanced resilience so soon after egressing from the biofilm.

## INTRODUCTION

Biofilm dispersion is the last stage of the biofilm developmental lifecycle, from which bacteria transition back to the motile state after escaping from the biofilm matrix. Dispersion has likewise been shown to be inducible in response to various external signals, including nitric oxide (NO), changes in concentration of oxygen and carbon and energy sources, pH, and exposure to the fatty acid signaling molecule *cis*-2-decenoic acid (1–4). Recent findings furthermore suggest that in *Pseudomonas aeruginosa,* native dispersion and dispersion in response to nitric oxide, glutamate, and *cis*-2-decenoic acid rely on similar factors, namely the chemisensory transducer BdlA, the phosphodiesterases DipA and RbdA, as well as AmrZ (5) . Upon sensing of dispersion cues, BdlA undergoes non-processive proteolysis and activation in a process requiring phosphorylation of BdlA, temporarily elevated c-di-GMP levels, and the protease ClpP, the chaperone ClpD (6, 7). BdlA, in turn, activates the phosphodiesterase DipA and recruits a second phosphodiesterase, RbdA, to ultimately reduce the intracellular level of the secondary messenger c-di-GMP (8, 9). The alginate and motility regulator AmrZ contributes to dispersion in a BdlA-dependent manner, likely by regulating genes affecting virulence, c-di-GMP levels, Pel and Psl abundance, and motility (10, 11).

Recent findings indicated dispersion signal transduction and regulatory mechanisms governing dispersion in response to exogenous dispersion signals such as nitric oxide and nutrients to also govern native dispersion (5). The findings suggest dispersed cells obtained in response to native and induced dispersion are likely to adopt similar phenotypic traits relative to biofilm cells from which they egressed. Relative to biofilm and planktonic cells, dispersed cells have been reported to have reduced growth rates; however, Chua et al. (12) reported *P. aeruginosa* dispersed cells demonstrate an extended lag phase, after which dispersed cells displayed growth rates comparable to planktonic bacteria. Dispersed cells have furthermore been shown to have a hyperadhesive phenotype, apparent by *P. aeruginosa* and *Marinobacter hydrocarbonoclasticus* dispersed cells demonstrating increased attachment to abiotic surfaces compared to the planktonic cells (13, 14). The enhanced attachment phenotype is likely linked to dispersed cells producing more matrix-degrading enzymes (15–17) while inversely regulating genes associated with the biosynthesis of exopolysaccharides, fimbriae, and flagella (4, 8, 18). Additional phenotypes associated with dispersed cells transitioning toward the motile lifestyle include changes in antibiotic susceptibility relative to biofilms (14, 18). However, increased susceptibility was found to be somewhat dependent on the antibiotic used (14). For example, dispersed cells by *P. aeruginosa* are susceptible to tobramycin and colistin when dispersed in response to nitric oxide but resistant to colistin when dispersed in response to glutamate (14).

In contrast, however, dispersed cells by *Streptococcus mutans* cells retained their resistance to chlorohexidine and acid treatments (1, 2). Additionally, dispersed cells have been reported to be more virulent than planktonic cells when tested using various acute and chronic virulence models (12, 19–21). Increased virulence has been linked to dispersed cells’ increased ability to escape and kill macrophages. The phenotypes of dispersed cells suggested dispersed cells to be distinct from biofilm and planktonic cells, which is further supported by dispersed cells exhibiting protein production and gene expression profiles distinct from biofilms from which they escaped (12, 14, 19, 21, 22). Using single-nucleotide resolution transcriptomic analysis of *P. aeruginosa* 5 hours after induction of dispersal by nitric oxide or upon overproduction of the *Escherichia coli* phosphodiesterase YhjH, Chua et al. (12) found dispersed cells to differ significantly from both planktonic and biofilm cells. Examination of the transcriptome by principal component analysis (PCA) revealed that the dispersed cells have distinct physiology compared to planktonic and biofilm cells. This was apparent by dispersed cells differing from planktonic cells by the differential expression of approximately 630 genes, 353 genes that were upregulated, and 280 genes that were downregulated in both dispersal conditions compared with planktonic cells (12). Specifically, planktonic cells exhibited a higher expression level of the genes involved in *las* and *rhl* quorum sensing than biofilm and dispersed cells.

In contrast, biofilm cells exhibited a higher expression level of the genes involved in matrix protein synthesis (for example, *cdrA*, *cdrB*) and a lower expression level of the genes involved in motility and chemotaxis (for example, *pilA*, *fliC*, *aer*) compared with planktonic and dispersed cells. Additional differences included the expression of the small regulatory RNAs RsmY and RsmZ, which were downregulated in dispersed cells relative to planktonic and biofilm cells, whereas type II secretion system (TTSS) genes were found to be induced (12). Moreover, dispersed cells exhibited a higher expression level of the genes involved in virulence (for example, *secB*) and a lower expression level of the genes involved in iron uptake and iron stress, apparent by the expression of genes encoding the major siderophore pyoverdine being downregulated in dispersed cells relative to planktonic and biofilm cells (12).

While it is clear from the above studies that the phenotype of dispersed cells is distinct from planktonic and biofilm cells 5 hours post dispersion, little is known about the phenotype of dispersed cells within minutes of egressing from the biofilm, and if such freshly dispersed cells already express traits, similar to older dispersed cells, that distinguish them as a transitionary phenotype from both biofilm and planktonic cells. Understanding the dispersion transcriptome and the phenotypic traits of dispersed cells will be crucial, however, for developing dispersion as a potential treatment strategy for biofilm-related infections. Moreover, understanding how dispersed cells adapt over time toward the planktonic phenotype is important. In this study, we elucidated the transcriptome and physiology associated with freshly dispersed *P. aeruginosa* cells, which were obtained within less than 30 minutes of dispersion. We employed RNA sequencing to elucidate the molecular signatures specific to biofilm, planktonic, and dispersed cells and to identify gene expression patterns that distinguish dispersed cells from their parent biofilms and planktonic counterparts. To gain insight into how dispersed cells transition to the planktonic mode, we furthermore compared freshly dispersed cells to dispersed cells that remained exposed to the dispersion signals for 5 hours (12).

## RESULTS AND DISCUSSION

### The transcriptome of freshly dispersed cells is distinct from biofilm and planktonic cells, regardless of the dispersion inducer used

To unravel the phenotype of freshly dispersed cells and provide a comprehensive description of what a dispersed cell is, we first employed RNA sequencing of *Pseudomonas aeruginosa* dispersed cells to elucidate the molecular signatures specific to dispersed cells relative to biofilm and planktonic cells and identify gene expression patterns that distinguish dispersed cells from their parent biofilms and planktonic counterparts. Dispersed cells were obtained from biofilms in response to the dispersion inducers nitric oxide and glutamate, and the respective dispersed cells were collected within less than 30 minutes post induction of dispersion.

**Fig 1:**
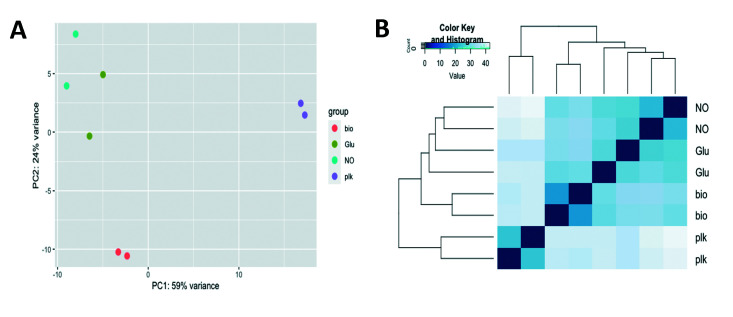
Gene expression analysis of freshly dispersed cells. (**A**) PCA of gene expression of *P. aeruginosa* planktonic cells, dispersed cells, and biofilm cells. Each cell type is shown by a different color. Individual data points represent biological RNA-seq replicates; two independent repeats were used for bio, biofilm; Glu, dispersed cells obtained in response to glutamate; NO, dispersed cells obtained in response to nitric oxide; plk, planktonic cells. (**B**) Heatmap and hierarchical clustering analysis. Raw data of RNA-seq were normalized using the DESeq package before clustering and PCA. The differentially expressed genes (fold change >±2 log2, adjusted *P*-value <0.05) between any pairs of dispersed cells, planktonic cells, and biofilm cells were identified by performing a negative binomial test using the DESeq package of R/Bioconductor. The full list of genes that were differentially expressed between any pairs of the above groups is provided in Table S1.

To explore the relationships between the three different modes of growth, we first evaluated the transcriptomic data using PCA. While PCA can be used to identify outlying samples for quality control, it can also uncover trends and clusters between samples and uncover relationships between variables, such as dispersion. Here, the PCA revealed the largest source of variation to be the mode of growth, accounting for 59% of the total variance between the biofilms (sessile) and planktonic (motile) cells. The data points separated along the first principal component (PC1) (Fig. 1A), confirming previous reports of biofilm and planktonic cells differing with respect to their gene expression profile. Furthermore, the transcriptome of dispersed cells, obtained in response to nitric oxide and glutamate, clustered separately from biofilm and planktonic cells. Specifically, the cluster representing the transcriptome of dispersed cells was separated from the biofilm cells cluster on the second principal component (PC2), accounting for 24% of the variance (Fig. 1A). The findings indicate freshly dispersed cells to have a distinct gene expression profile compared to the biofilm cells from which they egressed. PCA revealed even larger variations between dispersed cells and planktonic cells, apparent by the separation on the PC1 (Fig. 1A), suggesting dispersed cells, despite having returned to the motile lifestyle, are distinct from planktonic cells. Findings obtained by PCA were supported by hierarchical clustering analysis, supporting the transcriptome of dispersed cells to be more distinct from planktonic cells than biofilm cells (Fig. 1B).

**TABLE 1:** The dispersion transcriptome^*a*^

Locus ID	Description	Fold change (log2)			
		Glutamate dispersed cells		NO dispersed cells	
PA0506	Acyl-CoA dehydrogenase	Upregulated	1.574357	Upregulated	1.223554
PA0840	Oxidoreductase	Upregulated	1.83432	Upregulated	2.018073
PA1093	Hypothetical protein	Upregulated	1.293367	Upregulated	1.228576
PA1094	FliD-B type flagellar hook-associated protein	Upregulated	0.966737	Upregulated	0.838656
PA1136	Transcriptional regulator	Upregulated	2.874245	Upregulated	3.124099
PA1137	Oxidoreductase	Upregulated	3.332602	Upregulated	3.866577
PA1581	sdhC-succinate dehydrogenase subunit C	Upregulated	1.123353	Upregulated	0.880398
PA1787	acnB-aconitate hydratase B	Upregulated	1.081276	Upregulated	0.702492
PA2197	Conserved hypothetical protein	Upregulated	0.710304	Upregulated	1.153924
PA2550	Acyl-CoA dehydrogenase	Upregulated	1.42034	Upregulated	1.157598
PA2561	Methyl-accepting chemotaxis protein-ctpH	Upregulated	1.031822	Upregulated	1.155627
PA2736.1	tRNA-pro	Upregulated	0.349694	Upregulated	0.895849
PA2840	ATP-dependent RNA helicase	Upregulated	2.776142	Upregulated	2.419303
PA3160	O-antigen chain length regulator	Upregulated	0.409712	Upregulated	0.700575
PA3183	zwf-Glucose-6-phosphate 1-dehydrogenase	Upregulated	0.992333	Upregulated	1.245576
PA3194		Upregulated	1.003429	Upregulated	1.268914
PA3234	Acetate permease	Upregulated	0.850115	Upregulated	0.722684
PA3235		Upregulated	1.168466	Upregulated	1.139177
PA3742	rpIS-50S ribosomal protein L19	Upregulated	1.238263	Upregulated	1.159481
PA3920	Metal transporting P-type ATPase	Upregulated	1.274297	Upregulated	1.608189
PA4239		Upregulated	1.027846	Upregulated	0.613988
PA4244		Upregulated	1.057922	Upregulated	0.741539
PA4245	rpmD-50S ribosomal protein L30	Upregulated	1.083746	Upregulated	0.966522
PA4248		Upregulated	1.036283	Upregulated	0.702379
PA4249	rpsH-30S ribosomal protein S8	Upregulated	1.439878	Upregulated	1.194785
PA4255	rpmC-50S ribosomal protein L29	Upregulated	0.987271	Upregulated	0.700314
PA4256	rpIP-50S ribosomal protein L16	Upregulated	1.448582	Upregulated	1.059879
PA4261	rpIW-50S ribosomal protein L23	Upregulated	1.18652	Upregulated	0.891766
PA4266	fusA1-elongation factor G	Upregulated	1.157333	Upregulated	0.617394
PA4267	rpsG-30S ribosomal protein S7	Upregulated	1.082922	Upregulated	0.61328
PA4273	rpIA-50S ribosomal protein L1	Upregulated	1.236553	Upregulated	0.816959
PA4274	rpIK-50S ribosomal protein L11	Upregulated	1.255703	Upregulated	0.907037
PA4386		Upregulated	0.483919	Upregulated	1.052386
PA4430	Cytochrome B	Upregulated	1.244303	Upregulated	0.843705
PA4658		Upregulated	0.939783	Upregulated	1.015749
PA5316		Upregulated	1.352634	Upregulated	1.400194
PA5367	pstA-phosphate ABC transporter permease	Upregulated	2.119658	Upregulated	1.54155
PA5368	pstC-phosphate ABC transporter permease	Upregulated	1.776169	Upregulated	1.306284
PA5369	pstS-(PA5369) phosphate ABC transporter substrate-binding protein	Upregulated	2.380728	Upregulated	1.58242
PA0008	GlyS-glysyl-tRNA synthetase	Upregulated	1.025369	Upregulated	0.840142
PA0140	Alkylhydroperoxide reductase subunit F	Upregulated	1.296643	Upregulated	0.635864
PA0283	Sulfate binding protein precursor	Upregulated	0.569928	Upregulated	2.084621
PA0432	SahH-S-adenosyl-L-homocysteine hydrolase	Upregulated	0.555658	Upregulated	0.100262
PA0508	Probable acyl-CoA dehydrogenase	Upregulated	1.638803	Upregulated	1.018265
PA0546	Methionine adenosyltransferase	Upregulated	0.776164	Upregulated	0.340434
PA0548	Transketolase	Upregulated	0.64417	Upregulated	0.129716
PA0729.1	tRNA-Gly	Upregulated	0.784979	Upregulated	1.081866
PA0833	Hypothetical protein	Upregulated	0.927836	Upregulated	1.258061
PA0888	aotJ-arginine/ornithine binding protein AotJ	Upregulated	0.842525	Upregulated	0.372018
PA0889	aotQ-arginine/ornithine transport protein AotQ	Upregulated	1.193255	Upregulated	0.246144
PA0898	aruD-sucinylglutamate 5-semialdehyde dehydrogenase	Upregulated	1.097161	Upregulated	0.371946
PA0916	Conserved hypothetical protein	Upregulated	1.291185	Upregulated	0.643439
PA1076	Hypothetical protein		−0.10099	Upregulated	0.1182
PA1099	Two component response regulator	Upregulated	0.84346	Upregulated	1.007035
PA1288	Xylipin transporter	Upregulated	0.999622	Upregulated	0.703785
PA1317	cyoA-cytochrome o ubiquinol oxidase subunit II	Upregulated	1.24985	Upregulated	0.820095
PA1318	cyoB-cytochrome o ubiquinol oxidase subunit 1	Upregulated	0.840862	Upregulated	0.713546
PA1340	AatM	Upregulated	1.068743	Upregulated	0.607256
PA1551	Probable ferredoxin	Upregulated	1.101473	Upregulated	1.379079
PA1552	Cytochrome c oxidase, cbb3-type CcoP subunit	Upregulated	1.176545	Upregulated	0.800953
PA1553	Cytochrome c oxidase, cbb3-type, coO subunit	Upregulated	0.973052	Upregulated	0.658904
PA1554	Cytochrome c oxidase, cbb3-type, coN subunit	Upregulated	1.344648	Upregulated	0.834431
PA1585	2-Oxoglutarate dehydrogenase (E1 subunit)	Upregulated	0.590433	Upregulated	0.257621
PA1588	Succinyl-coA synthetase beta chain	Upregulated	0.803002	Upregulated	0.393744
PA1673	mhr-microoxic hemerythrin, Mhr		−0.71481	Upregulated	0.284462
PA1796.4	tRNA-His	Upregulated	0.659719	Upregulated	0.691559
PA1984	exaC	Upregulated	0.803372	Upregulated	0.446629
PA2147	katE-catalase HPII	Upregulated	0.960918	Upregulated	0.105907
PA2252	Probable AGCS sodium/alanine/glycine symporter	Upregulated	1.003606	Upregulated	0.372598
PA2567	Hypothetical protein	Upregulated	1.437009	Upregulated	1.697091
PA2624	Isocitrate dehydrogenase	Upregulated	0.834501	Upregulated	0.710761
PA2638	nuoB-NADH dehydrogenase	Upregulated	1.339827	Upregulated	1.053319
PA2639	nuoD-NADH dehydrogenase I chain C,D	Upregulated	0.908409	Upregulated	0.38737
PA2642	nuoG-NADH dehydrogenase I chain G	Upregulated	0.932978	Upregulated	0.532292
PA2647	nuoL-NADH dehydrogenase I chain L	Upregulated	0.971877	Upregulated	0.588869
PA2649	nuoN-NADH dehydrogenase I chain N	Upregulated	0.842895	Upregulated	0.823345
PA2740	pheS-phenylalanine-tRNA synthetase, alpha-subunit	Upregulated	0.866551	Upregulated	0.452265
PA2754	Conserved hypothetical protein		−0.60057	Upregulated	0.480409
PA2830	Heat shock protein HtpX	Upregulated	0.487969	Upregulated	1.124719
PA2976	Ribonuclease E	Upregulated	0.68855	Upregulated	0.378461
PA3044	Two component sensor RocS2		−0.81144	Upregulated	0.056449
PA3068	gdhB-NAD-dependent glutamate dehydrogenase	Upregulated	0.628916	Upregulated	0.11409
PA3126	Heat shock protein IbpA	Upregulated	0.061266	Upregulated	0.868018
PA3262.1	tRNA-Asp	Upregulated	0.590536	Upregulated	0.338509
PA3385	amrZ-alginate and motility regulator Z	Upregulated	1.734455	Upregulated	1.610377
PA3418	ldh-leucine dehydrogenase		−0.35858	Upregulated	0.014165
PA3637	CTP synthase	Upregulated	0.880197	Upregulated	0.62778
PA3700	Lysyl-tRNA synthetase	Upregulated	1.021677	Upregulated	0.73513
PA3768	Probable metallo-oxidoreductase		−0.73303	Upregulated	0.061489
PA3769	GMP synthase	Upregulated	0.878636	Upregulated	0.30658
PA3770	Inosine-5'-monophosphate dehydrogenase	Upregulated	0.855098	Upregulated	0.448326
PA3814	L-cysteine desulfurase (pyridoxal phosphate-dependent)	Upregulated	0.76699	Upregulated	0.410849
PA3913	Probable protease	Upregulated	0.797904	Upregulated	1.297057
PA3930	Cyanide insensitive terminal oxidase	Upregulated	1.13987	Upregulated	0.258596
PA3972	Probable acyl-coA dehydrogenase	Upregulated	1.014161	Upregulated	1.2143
PA4053	6,7-Dimethyl-8-ribityllumazine synthase	Upregulated	0.797768	Upregulated	0.456159
PA4131	Probable iron sulfur protein	Upregulated	1.332596	Upregulated	0.423339
PA4133	Cytochrome c oxidase subunit (cbb3 type)	Upregulated	0.661268	Upregulated	0.344249
PA4237	50S ribosomal protein L17	Upregulated	0.924128	Upregulated	0.695099
PA4252	50S ribosomal protein L24	Upregulated	0.815203	Upregulated	0.508448
PA4259	30S ribosomal protein S19	Upregulated	0.840249	Upregulated	0.998411
PA4265	Elongation factor Tu	Upregulated	0.678767	Upregulated	0.365132
PA4269	DNA directed RNA Polymerase beta chain	Upregulated	0.680811	Upregulated	0.298718
PA4270	DNA directed RNA Polymerase beta chain	Upregulated	0.546756	Upregulated	0.231753
PA4277	Elongation factor Tu	Upregulated	0.831075	Upregulated	0.498717
PA4333	Probable fumarase	Upregulated	0.849468	Upregulated	0.634796
PA4352	Conserved hypothetical protein		−0.9037	Upregulated	0.078307
PA4356	Xenobiotic reductase		−0.51786	Upregulated	0.100857
PA4385	GroEL protein	Upregulated	0.555218		0.900314
PA4429	Probable cytochrome c1 precursor	Upregulated	0.950586	Upregulated	0.480124
PA4431	Probable iron-sulfur protein	Upregulated	0.893213	Upregulated	0.335328
PA4519	Ornithine decarboxylase	Upregulated	0.781826	Upregulated	0.334712
PA4598	Resistance-nodulation-cell division (RND) multidrug efflux transporter MexD	Upregulated	1.737234	Upregulated	0.604697
PA4602	Serine hydroxymethyltransferase	Upregulated	1.056377	Upregulated	0.755105
PA4758	Carbamoyl-phosphate synthase small chain	Upregulated	0.761742	Upregulated	0.975523
PA4759	Dihydrodipicolinate reductase	Upregulated	0.910949	Upregulated	1.203546
PA4761	DnaK protein	Upregulated	0.152286	Upregulated	0.941725
PA4855	Phosphoribosylamine-glycine ligase	Upregulated	1.202422	Upregulated	0.740721
PA4929	NicD	Upregulated	1.348215	Upregulated	1.124534
PA5015	Pyruvate dehydrogenase	Upregulated	0.599174	Upregulated	0.454132
PA5053	Heat shock protein HslV	Upregulated	1.34497	Upregulated	1.759096
PA5098	hutH-histidine ammonia-lyase	Upregulated	1.021263	Upregulated	0.482614
PA5119	glnA-glutamine synthetase	Upregulated	0.474793	Upregulated	0.541001
PA5315	50S ribosomal protein L33	Upregulated	0.84785	Upregulated	0.761561
PA5366	ATP binding component of ABC phosphate transporter	Upregulated	1.401961	Upregulated	0.735852
PA5427	Alcohol dehydrogenase		−0.79947	Upregulated	0.06287
PA5490	cc4-cytochrome c4 precursor	Upregulated	0.897352	Upregulated	0.531742
PA5495	Homoserine kinase		−0.04312	Upregulated	0.320808
PA0027	Hypothetical protein	Downregulated	−1.14918	Downregulated	−1.38885
PA0039	Hypothetical protein	Downregulated	−0.97711	Downregulated	−1.29894
PA0078	tssL1	Downregulated	−1.61908	Downregulated	−1.653
PA0396	pilU twitching motility protein	Downregulated	−0.81476	Downregulated	−1.03689
PA0451	Conserved hypothetical protein	Downregulated	−1.49988	Downregulated	−0.92378
PA0553	Hypothetical protein	Downregulated	−0.78642	Downregulated	−0.65239
PA0657	Probable ATPase	Downregulated	−1.54727	Downregulated	−1.28335
PA0665	Iron-sulfur cluster insertion protein ErpA	Downregulated	−0.54209	Downregulated	−0.56788
PA0668.5	5S ribosomal RNA	Downregulated		Downregulated	
PA0672	Heme oxygenase	Downregulated	−0.49761	Downregulated	−0.14531
PA0905.1	tRNA-Ser	Downregulated	−1.15073	Downregulated	−1.1498
PA1013.1	tRNA-ser	Downregulated	−0.45042	Downregulated	−0.31687
PA1134	Hypothetical protein	Downregulated	−1.22292	Downregulated	−0.28335
PA1159	Cold shock protein	Downregulated	−1.30326	Downregulated	−1.63393
PA1248	aprF-alkaline protease secretion protein AprF	Downregulated	−1.33189	Downregulated	−1.04807
PA1300	ECF subfamily sigma-70 factor	Downregulated	−1.44306	Downregulated	−0.88122
PA1301	Transmembrane sensor	Downregulated	−1.7332	Downregulated	−1.29064
PA1392	Hypothetical protein	Downregulated	−1.75365	Downregulated	−1.13984
PA1530.1	ffs			Downregulated	
PA1604	Hypothetical protein	Downregulated	−1.08326	Downregulated	−0.02943
PA1796.3	tRNA-Leu	Downregulated	−0.9461	Downregulated	−0.38983
PA2034	Hypothetical protein	Downregulated	−1.29795	Downregulated	−0.98811
PA2389	pvdR-pyoverdine biosynthesis PvdR	Downregulated	−1.32394	Downregulated	−1.31531
PA2570.1	tRNA-Leu	Downregulated	−0.24898	Downregulated	−0.61465
PA2591	vqsR	Downregulated	−0.86386	Downregulated	−0.70927
PA2662	Conserved hypothetical protein		1.397852	Downregulated	−0.46411
PA2738	himA-integration host factor subunit alpha	Downregulated	−0.96275	Downregulated	−0.95322
PA2852.1		Downregulated	−1.98698	Downregulated	−0.28953
PA3104	xcpP-secretin protein XcpP	Downregulated	−0.87546	Downregulated	−0.68226
PA3115	fimV-Motility protein FimV	Downregulated	−0.58301	Downregulated	−0.53673
PA3224	Hypothetical protein	Downregulated	−0.99838	Downregulated	−0.28753
PA3309	Conserved hypothetical protein	Downregulated	−1.17036	Downregulated	−0.51119
PA3417	Probable pyruvate dehydrogenase E1 component, alpha subunit	Downregulated	−1.36532	Downregulated	−0.31572
PA3530	Hypothetical protein	Downregulated	−1.93133	Downregulated	−1.08977
PA3749	Probable major facilitator superfamily (MFS) transporter	Downregulated	−1.75895	Downregulated	−1.958
PA3945	Conserved hypothetical protein	Downregulated	−0.73707	Downregulated	−0.49597
PA4132	MpaR		0.800486	Downregulated	−0.02167
PA4168	Second ferric pyoverdine receptor FpvB	Downregulated	−1.03613	Downregulated	−0.6785
PA4227	pchR-transcriptional regulator PchR	Downregulated	−2.02201	Downregulated	−1.43216
PA4404		Downregulated	−1.45882	Downregulated	−1.56322
PA4470	fumC1-fumarate hydratase	Downregulated	−1.57899	Downregulated	−0.9378
PA4471	Hypothetical protein	Downregulated	−1.40406	Downregulated	−0.61311
PA4550		Downregulated	−1.27935	Downregulated	−1.05859
PA4607		Downregulated	−1.16974	Downregulated	−1.67189
PA4613	katB, catalase		1.582839	Downregulated	−0.20076
PA4614		Downregulated	−0.64012	Downregulated	−0.85867
PA4690.1	5S ribosomal RNA	Downregulated		Downregulated	
PA4709	phuS	Downregulated	−1.41225	Downregulated	−0.71866
PA5039	aroK-shikimate kinase	Downregulated	−1.2024	Downregulated	−1.08712
PA5208	Conserved hypothetical protein	Downregulated	−0.39574	Downregulated	−0.30675
PA5227.1	ssrS-ncRNA	Downregulated	−1.38714	Downregulated	−1.31138
PA5369.1	5S ribosomal RNA	Downregulated		Downregulated	
PA5475	Hypothetical protein	Downregulated	−0.87033	Downregulated	−0.06788
PA0256	Hypothetical protein	Downregulated	−0.22622	Downregulated	−0.40584
PA0423	PasP	Downregulated	−0.75411	Downregulated	−0.74138
PA0706	Cat-chloramphenicol acetyltransferase	Downregulated	−0.70645	Downregulated	−1.06702
PA0867	Membrane bound lysozyme inhibitor of c-type lysozyme MilC	Downregulated	−0.96531	Downregulated	−1.32176
PA1245	AprX	Downregulated	−0.82295	Downregulated	−0.86183
PA1430	Transcriptional regulator LasR	Downregulated	−0.35513	Downregulated	−0.42317
PA1804	DNA binding protein HU	Downregulated	−0.48589	Downregulated	−0.7316
PA1869	Acp1	Downregulated	−0.82325	Downregulated	−1.13489
PA3623	Conserved hypothetical protein	Downregulated	−0.75899	Downregulated	−0.6439
PA3808	Conserved hypothetical protein	Downregulated	−0.44342	Downregulated	−0.86057
PA4408	Cell division protein FtsA	Downregulated	−0.51826	Downregulated	−0.96422
PA4466	Probable phosphoryl carrier protein	Downregulated	−0.10875	Downregulated	−0.17412
PA4876	smE	Downregulated	−0.26483	Downregulated	−0.46575

^*a*^ Transcript abundance of genes that are uniquely expressed in glutamate and nitric oxide dispersed cells, relative to biofilm and planktonic cells. Genes uniquely expressed in freshly dispersed cells are shown. Fold change (log2) is relative to biofilm cells. Genes were identified using a negative binomial test with a significance threshold of *P*-value 0.05.

Our findings of freshly dispersed cells displaying distinct expression patterns relative to biofilm and planktonic cells indicated dispersed cells to adopt a unique phenotype within minutes of egressing from the biofilm.

### Comparison of freshly dispersed cells and dispersed cells obtained 5 hours post-exposure to dispersion signals

Previous findings indicated that dispersed cells that remained exposed to the dispersion signals for 5 hours are distinct from planktonic and biofilm cells, with dispersed cells representing a distinct stage in transitioning from bacterial biofilm to planktonic lifestyle (12). Similarly, our findings indicated that freshly dispersed cells display distinct expression patterns relative to biofilm and planktonic cells (Fig. 1) and, what’s more, share many similarities with dispersed cells that were exposed for 5 hours to dispersion signals (12). This raised the question of similarity between freshly dispersed cells and dispersed cells obtained 5 hours post-exposure to dispersion signals and whether continued exposure to dispersion signals affects dispersed cells’ transcriptome.

**Fig 2:**
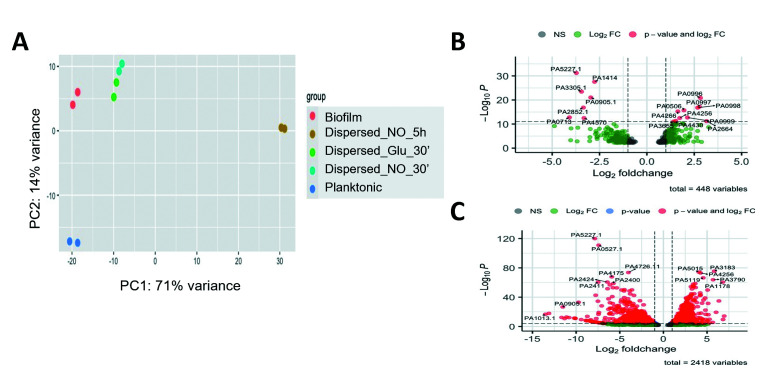
Comparison of the transcriptome of freshly dispersed cells to previously reported transcriptome of dispersed cells obtained 5 hours post dispersion. (**A**) PCA. PCA of gene expression of *P. aeruginosa* planktonic cells, freshly dispersed cells obtained in response to glutamate and nitric oxide, dispersed cells obtained post-exposure to dispersing inducing conditions for 5 hours, and biofilm cells. Transcriptomic data of dispersed cells obtained post-exposure to dispersion-inducing conditions for 5 hours was obtained from Chua et al. (12) and compared to transcriptomic data of biofilms obtained in this study. Each cell type is shown by a different color. Individual data points represent biological RNA-seq replicates; two independent repeats were used for biofilms, planktonic cells, dispersed cells obtained within 30 minutes in response to glutamate (Dispersed_Glu_30') or nitric oxide (Dispersed_NO_30'), or obtained 5 hours post dispersion in response to nitric oxide (Dispersed_NO_5 h). (**B–C**) Volcano plots, generated by the Enhanced Volcano package using a DESeq2 data set, with default log fold change thresholds of −1 and 1 and an adjusted *P*-value threshold of 0.05. The cutoff value for log2 fold change (log2FC) was >|2|, and the cutoff value for *P*-value was 10e − 5. Only the variables passing the log2FC and *P*-value thresholds are colored red. (**B**) Volcano plot of transcriptomic changes in PAO1 biofilms versus freshly dispersed cells obtained in response to nitric oxide. (**C**) Volcano plot of transcriptomic changes in PAO1 biofilms versus dispersed cells obtained post-exposure to the dispersion signal nitric oxide for 5 hours. Transcriptomic data of 5-hour nitric oxide dispersed cells were obtained from Chua et al. (12) and compared to transcriptomic data of biofilms obtained in this study.

We therefore evaluated the transcriptomic data of the two different types of dispersed cells relative to biofilms using PCA. The PCA revealed little separation of freshly dispersed cells, obtained in response to nitric oxide and glutamate, along the PC2 (Fig. 2A). Similarly, dispersed cells obtained 5 hours post induction of dispersion formed a tight cluster (Fig. 2A). Both types of dispersed cells clustered away from planktonic cells, accounting for 14% of the variance (Fig. 2A). However, the largest source of variation was noted to be the time of dispersed cells post egressing from the biofilm, accounting for 71% of the variance (Fig. 2A). This was apparent by the transcriptomes of dispersed cells obtained 5 hours post induction of dispersion separating along the PC1, away from biofilms, with the transcriptome of freshly dispersed cells, obtained in response to nitric oxide and glutamate, clustering much closer to that of biofilms (Fig. 2A).

To further explore similarities and differences between freshly dispersed cells and dispersed cells obtained 5 hours post induction of dispersion, we used volcano plots, comparing dispersed cells to biofilm cells. The volcano plots show statistical significance (*P*-value, adjusted *P*-value threshold of 0.05.) versus magnitude of change (base-2 log fold change). Relative to freshly dispersed cells obtained in response to nitric oxide (Fig. 2B), dispersed cells exposed to nitric oxide for 5 hours demonstrated a larger number of genes that are differentially expressed (Fig. 2C). Importantly, while freshly dispersed cells only differ from biofilm cells by 409 genes, extended exposure to nitric oxide appears to coincide with the differential expression of more than 2,000 genes (Fig. 2B and C). Similar results were obtained when freshly dispersed cells were evaluated in response to glutamate instead of nitric oxide (Fig. S1).

It is possible that differences in the experimental setup account for the noted difference in the transcriptome of 5-hour-old and freshly dispersed cells. The studies differed with respect to biofilm growth conditions. While the current study used biofilm grown under continuous growth conditions, biofilms reported by Chua et al. (12) were grown under semi-batch conditions for at least 3 days. More importantly, however, both growth conditions have been reported to enable the formation of biofilms with similar three-dimensional architecture. Moreover, both studies used similar growth media and dispersion inducers, physically separated dispersed cells from biofilms for sample collection, and any differences in sequencing were normalized. It is, therefore, more likely that the differences in the transcriptome reflect dispersed cells expanding or enhancing their distinct gene expression profile following egress from the biofilm, even after prolonged separation from the biofilm.

### Freshly dispersed cells have a unique physiology

Our comparative transcriptomic study revealed freshly dispersed cells obtained in response to nitric oxide or glutamate to demonstrate a distinct gene expression profile compared to the biofilm cells from which they egressed (Fig. 1). The PCA indicated that freshly dispersed cells were distinct from both biofilm and planktonic cells (Fig. 1). We, therefore, asked which genes are differentially expressed in freshly dispersed cells that make this group of cells so unique. Using a negative binomial test with a significance threshold of *P*-value 0.05, we identified a total of 194 genes that were differentially expressed in dispersed cells relative to both biofilm and planktonic cells, with 128 being upregulated and 65 downregulated. The respective genes are shown in Table 1. It is of interest to note that the number of uniquely expressed genes expanded to 633 genes (353 up, 280 down) post 5 hours of dispersion (12).

While the unique genes of freshly dispersed cells do not represent complete operons or pathways, genes that are significantly downregulated in dispersed cells suggest freshly dispersed cells limit twitching motility and pyoverdine biosynthesis. In contrast, genes that were found significantly upregulated in freshly dispersed cells relative to biofilm and planktonic cells comprised genes encoding 30S and 50S ribosomal proteins, c-di-GMP modulating enzymes, protein synthesis, transport, energy generation, chemotaxis, and flagellar-driven motility, as well as numerous genes related to metabolic activities (Table 1). The unique transcriptome of freshly dispersed cells thus supports the notion of dispersed cells differentiating from biofilms (and planktonic cells) at the time of dispersion via overall shifts in motility and protein synthesis and, more specifically, metabolic activities and energy generation while adjusting to iron availability.

**Fig 3:**
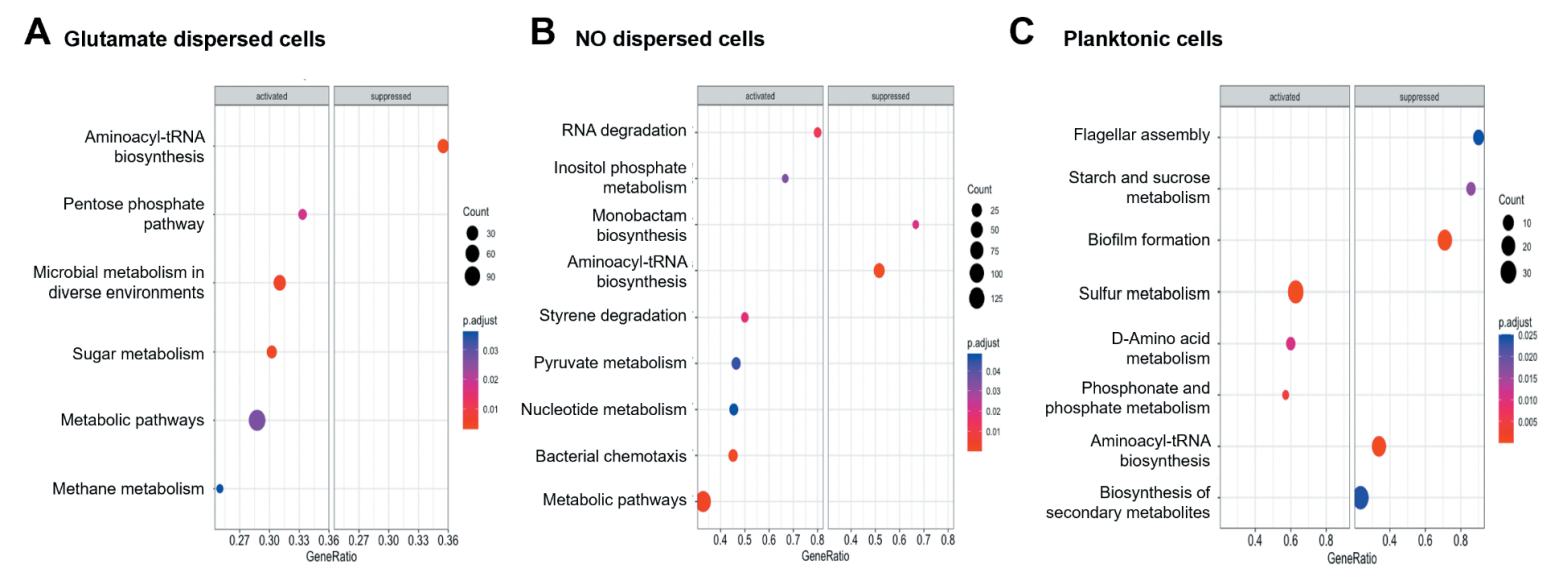
KEGG pathways enrichment in the dispersed cells (glutamate and nitric oxide) and planktonic cells compared with the biofilms. The top 10 upregulated and the top 10 downregulated pathways in (**A**) glutamate dispersed cells, (**B**) nitric oxide dispersed cells, and (**C**) planktonic cells. Only pathways with an adjusted *P*-value of less than 0.05 were considered significantly enriched.

Freshly dispersed cells were found to exhibit a set of differentially expressed genes (DEGs) that were distinct and related to the manner by which dispersion was achieved. For example, nitric oxide-induced dispersed cells displayed 102 DEGs at a significance level of *P* < 0.05, which were more strongly up/downregulated compared to glutamate-induced dispersed cells, while glutamate-induced dispersed cells exhibited 30 DEGs at a significance level of *P* < 0.05, that were more strongly up/downregulated compared to nitric oxide-induced dispersed cells (Table 1). This is apparent in Gene Ontology (GO) analysis. In the case of glutamate-dispersed cells, the top 10 enriched GO terms included ABC-type quaternary ammonium compound transporting activity, pyoverdine biosynthetic process, oxidoreductase activity, response to the metal ion, transmembrane transporter activity, and transporter activity, as depicted in the dot plot of the GO analysis (Fig. 3). GO terms enriched in NO dispersed cells primarily involved the regulation of iron transport, metal ion transport, monoatomic ion transport, cell death, autolysis, and programmed cell death (Fig. 3). In contrast, planktonic cells were characterized by transcription, DNA-templated transcription, transcription regulator activity, DNA binding transcription factor activity, peptide transmembrane transporter activity, siderophore biosynthetic process, and siderophore metabolic process (Fig. 3). It is important to note that expression of these genes was not directly related to nitric oxide or glutamate as planktonic cells exposed to the dispersion inducers failed to demonstrate differential expression of the respective genes of interest (Table S1).

In addition to uniquely expressed genes, freshly dispersed cells furthermore express genes differently from biofilms. These two sets of genes are further discussed below to provide a more detailed portrait of dispersed cells and how dispersed cells represent a distinct stage in the transition from bacterial biofilm to planktonic lifestyle. Specifically, we will address differential expression of genes associated with metabolic pathways and energy generation, shifts in motility, iron acquisition and signaling, c-di-GMP modulation, antibiotic resistance and oxidative stress, and virulence.

### Increased expression levels of energy-generating and pathways in freshly dispersed cells

Dispersed cells were characterized by their unique upregulation of genes related to energy generation and metabolic activities (Table 1). Specifically, freshly dispersed cells upregulated genes involved in the Entner-Doudoroff and pentose phosphate pathways (Table 2). Likewise, dispersed cells demonstrated upregulation of genes involved in the TCA and oxidative phosphorylation relative to biofilm cells (Table 2). The transcript abundance of genes contributing to the respective pathways was increased regardless of the dispersion inducer (nitric oxide, glutamate) used. Moreover, the transcriptome analysis indicated upregulation of nucleotide metabolism, pyruvate metabolism, inositol phosphate metabolism, and RNA degradation in dispersed cells. However, the extent to which the respective genes were upregulated differed in dispersed cells obtained in response to nitric oxide and glutamate (Table 2). In comparison, biofilms were found to preferentially activate fermentative pathways and found to downregulate the TCA cycle relative to planktonic cells (Table 2). Our findings agree with previous transcriptomic and metabolic studies indicating biofilms activate fermentative pathways leading to acetate production while harboring reduced TCA cycle intermediates in *P. aeruginosa* biofilms relative to their planktonic counterparts (23).

**TABLE 2:** Differential expression of genes in freshly dispersed cells linked to metabolic pathways and energy generation^*a*^

Locus ID	Product	Fold change (log2)		
		NO dispersed cells	Glutamate dispersed cells	Planktonic cells
Glycolysis/Entner-Doudoroff				
PA0555	Fructose-1,6-bisphosphate aldolase	0.72	0.38	0.0702
PA0887	Acetyl-coenzyme A synthetase	1.297	1.43	1.87
PA1587	Dihydrolipoamide dehydrogenase Lpd	0.666	0.38	−0.11
PA1984	NAD + dependent aldehyde dehydrogenase ExaC	1.63	0.962	0.424
PA2555	Probable AMP-binding enzyme	0.359	0.25	−0.886
PA3001	Probable glyceraldehyde-3-phosphate dehydrogenase	0.67	0.311	−0.257
PA3635	Alcohol dehydrogenase class III	0.591	0.376	−0.091
PA4022	Enolase	1.13	0.695	−0.18
PA4152	Probable hydrolase	0.797	−0.77	1.36
PA4239	Pyruvate kinase II	1.54	0.966	−0.87
PA4899	Probable aldehyde dehydrogenase	0.422	0.113	−1.51
PA5015	Pyruvate dehydrogenase	0.87	0.625	0.305
PA5016	Dihydrolipoamide acetyltransferase	0.644	0.346	0.117
PA5110	Fructose-1,6-bisphosphatase	0.526	0.269	−110
PA5131	Phosphoglycerate mutase	0.629	0.15	0.18
PA5192	Phosphoenolpyruvate carboxykinase	0.52	0.284	−1.59
TCA cycle				
PA4333	Probable fumarase	1.15	1.16	0.32
PA4640	Malate:quinone oxidoreductase	0.47	0.803	−0.55
PA5015	Pyruvate dehydrogenase	0.62	0.87	0.305
PA5016	Dihydrolipoamide acetyltransferase	0.346	0.644	0.117
PA5435	Probable transcarboxylase subunit	0.088	0.21	−1.44
PA5436	Probable biotin carboxylase subunit of a transcarboxylase	0.42	0.46	−1.58
Pentose phosphate pathway				
PA0330	Ribose 5-phosphate isomerase	0.77	0.28	−0.302
PA0548	Transketolase	0.96	0.432	−0.152
PA0555	Fructose-1,6-bisphosphate aldolase	0.72	0.389	0.07
PA0607	Ribulose-phosphate-3-epimerase	0.86	0.346	−0.54
PA1499	Conserved hypothetical protein	1.91	2.11	3.99
PA2261	Probable 2-ketogluconate kinase	1.21	2.65	3
PA3182	6-Phosphogluconolactonase	1.92	2.68	1.196
PA3183	Glucose-6-phosphate-1-dehydrogenase	1.26	1.46	1.03
PA3194	Phosphogluconate dehydratase	1.45	1.67	1.298
PA4670	Ribose-phosphate pyrophosphokinase	1.006	0.374	−1.22
PA4732	Glucose-6-phosphate isomerase	0.18	0.08	−0.281
PA5110	Fructose-1,6-bisphosphatase	0.526	0.269	−1.1
PA5322	Phosphomannomutase AlgC	0.58	0.052	−0.9
Oxidative phosphorylation				
PA0113	Probable cytochrome c oxidase assembly factor	0.434	0.111	−0.12
PA1317	Cytochrome o ubiquinol oxidase subunit II	1.64	0.809	0.03
PA1318	Cytochrome o ubiquinol oxidase subunit I	1.17	0.947	0.37
PA1319	Cytochrome o ubiquinol oxidase subunit III	1.47	1.55	−0.01
PA1320	Cytochrome o ubiquinol oxidase subunit IV	0.86	0.24	−0.19
PA1552	Cytochrome c oxidase, cbb3-type CcoP subunit	1.333	1.04	0.5
PA1553	Cytochrome c oxidase, cbb3-type CcoO subunit	1.19	0.71	0.015
PA1554	Cytochrome c oxidase, cbb3-type, CcoN subunit	1.67	0.74	0.12
PA1555	Cytochrome c oxidase, cbb3-type CcoP subunit	0.14	0.544	−0.48
PA1556	Cytochrome c oxidase, cbb3-type CcoO subunit	0.143	1.01	−0.51
PA1557	Cytochrome c oxidase, cbb3-type, CcoN subunit	0.109	0.68	−0.48
PA1581	Succinate dehydrogenase (C subunit)	1.282	1.12	−0.63
PA1582	Succinate dehydrogenase (D subunit)	1.11	0.84	−0.48
PA1583	Succinate dehydrogenase (A subunit)	0.84	0.64	−0.114
PA1584	Succinate dehydrogenase (B subunit)	0.436	0.24	−0.396
PA5553	ATP synthase epsilon chain	0.52	0.426	−0.89
PA5554	ATP synthase beta chain	1.03	0.874	−0.87
PA5555	ATP synthase gamma chain	0.98	0.696	−1.12
PA5556	ATP synthase alpha chain	1.12	0.72	−1.01
PA5558	ATP synthase B chain	0.57	0.354	−1.26
PA5557	ATP synthase delta chain	0.809	0.46	−1.09
PA5559	ATP synthase C chain	0.93	0.74	−1.007
PA5560	ATP synthase A chain	0.9	0.384	−1.3
PA1552.1	Cytochrome c oxidase, cbb3-type CcoQ subunit	1.02	0.71	−0.4

^*a*^ Fold change (log2) is relative to biofilm cells.

In addition to the upregulation of genes linked to energy-generating metabolic pathways, dispersed cells demonstrated increased expression of genes linked to oxidative phosphorylation, indicative of dispersed cells harnessing oxygen reduction to generate ATP. This was apparent by the upregulation of genes encoding various NADH dehydrogenases, cytochromes, and terminal oxidases (Tables 1 and 2). Oxidative phosphorylation playing a role in the dispersion response is in agreement with findings by Huynh et al. (2), indicating that dispersion, induced in response to glucose deprivation, was abolished upon exposure to the protonophore CCCP (carbonyl cyanide *m*-chlorophenyl hydrazone, also known as 3-chlorophenyl hydrazonomalononitrile), which interferes with the transmembrane electrochemical gradient and proton motive force in bacteria (24, 25), apparent by reduced ATP production and increased membrane permeability (24, 26–28).

Dispersed cells were found to upregulate *nirQ. nirQ* encodes denitrification regulatory protein (nitric oxide reductase), also known as ATP-related protein NirQ, which reduces nitric oxide to nitrous oxide (N_2_O) to avoid the accumulation of toxic NO in the cell. NirQ has also been reported to be necessary for posttranslational activation of NOR in *Pseudomonas stutzeri (29*). During the denitrification process, NirQ induces a concentration gradient of hydrogen ions through the cell membrane, which leads to the synthesis of ATP (30). In agreement with the role of NirQ, genes comprising the *nor* operon were highly upregulated in dispersed cells relative to biofilm cells (Table 2). However, the expression of *kdpB* associated with potassium ion (K^+^) transport was reduced (Table 2). Interestingly, the extent to which the *nor* genes were upregulated differed in dispersed cells obtained in response to nitric oxide and glutamate, with dispersed cells obtained in response to nitric oxide demonstrating the highest transcript abundance of *nirQ* and *nor* genes (Table 2).

Increased expression of genes associated with the utilization of the Entner-Doudoroff pathway, a high flux pathway, along with oxidative phosphorylation and other NAD(P)H-supplying reactions, suggested dispersed cells to either generate NAD(P)H to counteract oxidative stress or, alternatively, to ramp up their energy-generating pathways to metabolically become “awake” and meet their energy needs. The latter is in agreement with the significant increase in the transcript abundance of genes encoding ribosomal proteins by freshly dispersed cells (Tables 1 and 2), suggesting dispersed cells likely undergo a metabolic reconstruction as if they are emerging from a dormant state after egressing from the biofilm.

**Fig 4:**
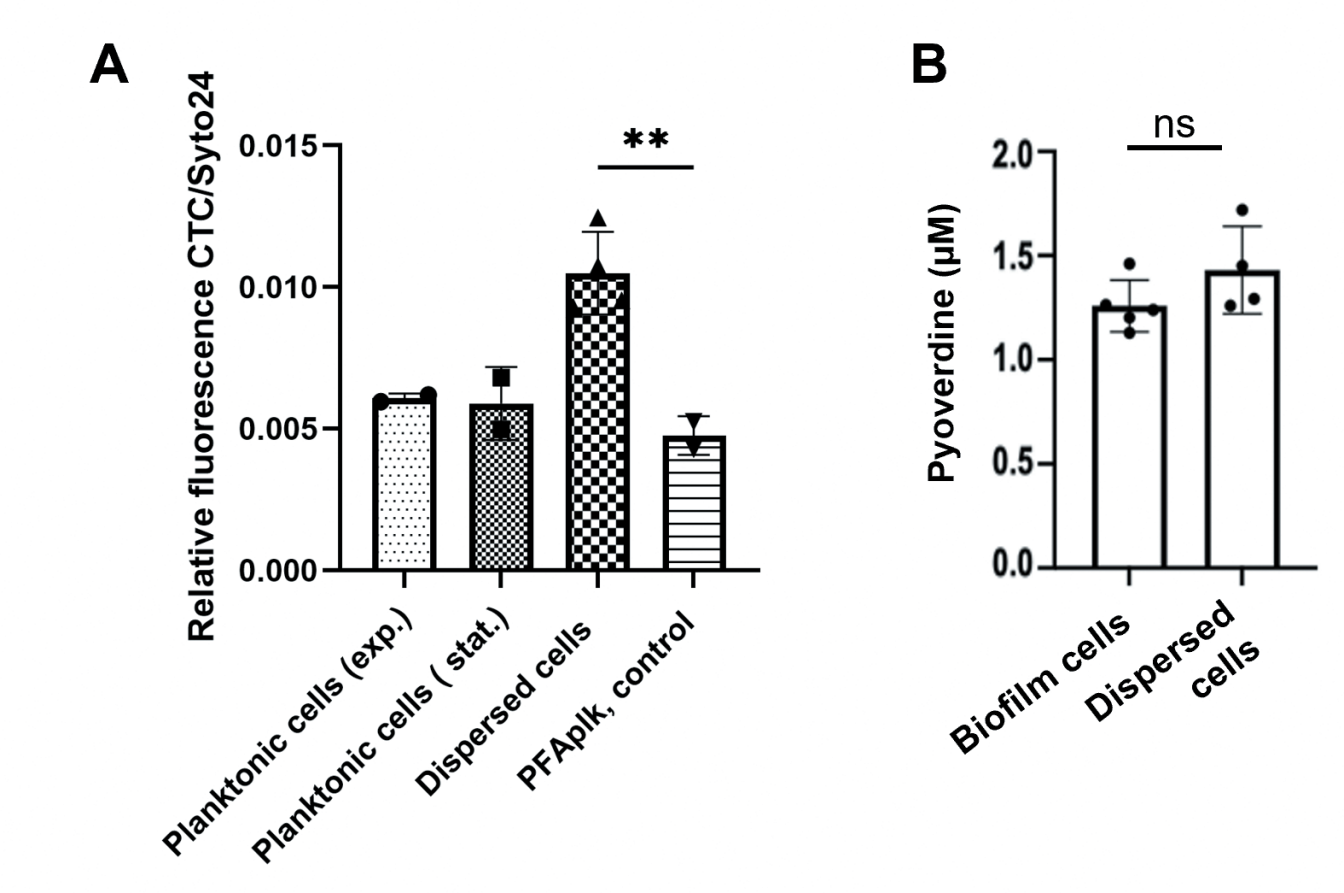
Metabolic and biochemical properties of dispersed cells. (**A**) Metabolic state of freshly dispersed cells. The metabolic activity of cells was monitored using CTC, which is reduced by respiratory enzymes to form red formazan crystals. The intensity of red fluorescence is proportional to the metabolic activity of cells. To normalize the metabolic activity of cells monitored by CTC, Syto24 was used as an internal control. Relative fluorescence was obtained by dividing the red fluorescence obtained from CTC by the green fluorescence obtained from Syto24 to correct any cell number or viability variations. Dispersed cells were obtained in response to nitric oxide. Planktonic cells were grown in Vogel-Bonner minimal medium to exponential (exp.) and stationary (stat.) phase. Planktonic cells treated with paraformaldehyde (PFA) were used as control (PFAplk). Experiments were carried out in triplicate using biological replicates. Statistical significance was determined by an analysis of variance, **, *P* < 0.01. (**B**) Abundance of pyoverdine in *P. aeruginosa* biofilm and freshly dispersed cells. Dispersed cells were obtained in response to nitric oxide. Pyoverdine was obtained by chloroform extraction. The top phase containing pyoverdine was utilized to measure the fluorescence as follows: excitation, 405 nm; emission, 460 nm. Experiments were carried out at least in triplicate using biological replicates. Statistical significance was determined by an unpaired *t*-test. ns, not significant.

To determine if increased expression of the various pathways correlated with increased energy generation and dispersed cells “awakening” to a more active state, we used 5-Cyano-2,3-ditolyl tetrazolium chloride (CTC) staining. The CTC staining method is frequently used for monitoring the metabolic activity of chemoorganotrophic bacteria (31), as well as respiratory activity (32), and is, therefore, a direct indicator of both oxidative metabolism as well as viability (31). Relative to planktonic cells grown to exponential and stationary phase, dispersed cells demonstrated increased CTC staining (Fig. 4A), confirming dispersed cells adopt a more highly energetic, active state. Thus, our findings indicate dispersion leads to higher levels of intracellular ATP and increased metabolic activity.

### Signs of reversion to the planktonic lifestyle in freshly dispersed cells

Dispersion by *P. aeruginosa* has been correlated with motile cells leaving the biofilm (4, 5, 12, 15). The unique dispersion transcriptome (Table 1) suggested freshly dispersed cells repress *pilU* (twitching motility protein) but increase the expression of *fliD* (B-type flagellar hook-associated protein), indicative of freshly dispersed cells undergoing a switch in motility. When considering additional genes that are differentially expressed relative to biofilms, we, therefore, expected dispersed cells to demonstrate increased transcript abundance of genes associated with a motile lifestyle, including genes associated with flagella biosynthesis and flagellar-driven motility, but reduced expression of genes associated with pili biosynthesis and biofilm matrix production. However, freshly dispersed cells were only found to upregulate some but not all flagellar genes (Table S2). Specifically, the expression of *fleSR* encoding a two-component sensor and regulator involved in flagellin synthesis was increased. Similarly, the transcript abundance of class IV genes, including *fliC*, *fliD*, and *flgMN* was significantly increased (Table S3). *fliC* encodes the flagella filament protein, *flgN* encodes the hook-filament junction chaperone protein, and *flgM* encodes sigma factor 28 (33, 34). In contrast, most flagellar genes belonging to class II and class III were found to be comparable or repressed relative to biofilm cells. Likewise, genes encoding the two sets of stators, MotAB and MotCD, with MotCD contributing to more stable motor speed, remained mostly unchanged. The findings suggested freshly dispersed cells only express the necessary genes to complete the assembly of the flagella filament (class IV) while making use of pre-existing class II and III proteins, including the flagellar body and rod proteins as well as the flagellum-specific ATP synthase to regain their motile state.

Flagellar-driven motility is inversely related to exopolysaccharide production (35). The *P. aeruginosa* PAO1 genome encodes three polysaccharides, Pel, Psl, and alginate, with Psl being the primary matrix polysaccharide produced by non-mucoid *P. aeruginosa* (36, 37), while mucoid strains primarily produce the alginate (38). While the transcript abundance of genes involved in the biosynthesis of Psl and alginate biosynthesis was modestly reduced in dispersed cells relative to biofilms, little difference in *pel* expression was noted. The exception was *pelA* encoding a bifunctional enzyme harboring both deacetylase (Pel biosynthesis) and hydrolase (Pel degradation) activities (39, 40). The transcript abundance of *pelA* was increased in dispersed cells relative to biofilms (Table S2). Interestingly, previous findings indicated overexpression of *pelA* to result in dispersion (15), with exogenously added PelA, resulting in the disassembly of the biofilm structure (40).

The trend of freshly dispersed cells demonstrating increased expression of flagella genes but reduced expression of pili and matrix components was likewise noted for dispersed cells 5 hours post dispersion (12). However, the two populations of dispersed cells differed in the extent to which the respective genes were differentially expressed. For example, while *fliC* expression increased less than twofold in freshly dispersed cells relative to biofilms (Table S2), much larger expression level changes of genes involved in motility and chemotaxis (for example, *pilA*, *fliC*, *aer*) were noted 5 hours post induction of dispersion (12).

### Dispersion affects iron acquisition and cell-to-cell signaling

Freshly dispersed cells demonstrated reduced expression of the *pvdR* gene associated with pyoverdine biosynthesis and iron acquisition (Table 1). Additional genes linked to pyoverdine biosynthesis and iron acquisition were identified when the transcriptome of freshly dispersed cells was compared, and freshly dispersed cells showed reduced expression of the iron starvation sigma factor, *pvdS*, as well as PvdS-regulated genes, such as *pvdG*, *pvdH*, *pchB*, *pchC*, *pchD*, *pchE*, *pchG*, *pchF* , and *fpvA,* relative to biofilms (Table S3). A similar trend was noted in 5-hour-old dispersed cells (12). However, while these genes were significantly 64-fold repressed in dispersed cells post 5-hour induction of dispersal (12), the difference in expression between freshly dispersed and biofilm cells was not significant. In agreement with these findings, no significant difference in pyoverdine levels was detected between freshly dispersed cells and biofilm cells (Fig. 4B). More significant changes in gene expression were noted for genes involved in cell signaling. For example, on average, freshly dispersed cells exhibited a two- to fourfold reduction in the gene expression of *lasA*, *lasR*, *rhlA*, *rhlB*, and *rhlR* in a manner similar to planktonic cells. In contrast, expression of genes involved in PQS biosynthesis was increased in freshly dispersed cells. Genes associated with PQS biosynthesis were among the most highly expressed genes in dispersed cells (Fig. 2C; Fig. S1). The finding of increased *pqs* gene transcript abundance is in agreement with previous reports of increased PQS production during biofilm dispersion in *P. aeruginosa* (41), with PQS, in turn, enhancing swimming motility by upregulating flagellar genes and dispersion (41). Interestingly, the expression of genes involved in PQS biosynthesis was significantly reduced post 5-hour induction of dispersal (12), suggesting a role of PQS to be limited to early events in the dispersion response.

### Freshly dispersed cells differentially express genes encoding c-di-GMP modulating enzymes

c-di-GMP has emerged as a key regulator of biofilm formation (42–44). c-di-GMP is a ubiquitous bacterial second messenger capable of regulating a myriad of cellular functions in bacteria, including group behavior such as (surface-associated) motility, surface adaptation, and biofilm formation (42–44). It is now widely recognized that high c-di-GMP levels favor the biofilm mode of growth, while low levels favor the motile, planktonic mode of growth (44). For example, high c-di-GMP levels regulate the production of the *P. aeruginosa* exopolysaccharides, Pel, and Psl (45), as well as the biosynthesis of fimbriae and pili, which assist in the initial attachment of planktonic cells to a surface or each other (46). In contrast, low levels of c-di-GMP have been shown to induce cell lysis, which increases the amount of eDNA available in the biofilm matrix and biofilm dispersal (47). The level of c-di-GMP in the cell is contingent on the activity of two groups of enzymes: diguanylate cyclase (DGC) and PDE (48–50). DGCs, with a canonical GG(D/E)EF motif, synthesize c-di-GMP from two molecules of GTP, while PDEs, with a canonical E(A/E/V)L motif, degrade c-di-GMP to pGpG (51) which is hydrolyzed into two GMP molecules by the oligoribonuclease Orn (52, 53). More recently, proteins with an HD-GYP domain were also shown to demonstrate c-di-GMP phosphodiesterase activity (54).

**Fig 5:**
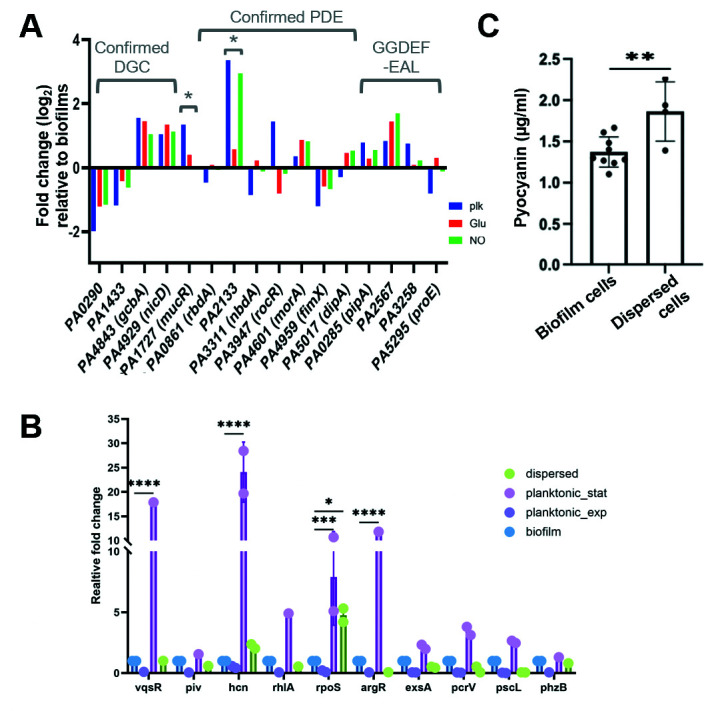
Transcript abundance of select genes linked to virulence and c-di-GMP modulation. (**A**) Transcript abundance of various enzymes that modulate c-di-GMP in dispersed and planktonic cells relative to the biofilms, based on RNA-seq. The enzymes are categorized into DGCs and PDEs. Where known, enzymes are categorized as DGC or PDE. MucR has been shown to harbor both DGC and PDE activity (5). Enzymes that have not been well characterized are listed by the presence of the GGDEF and/or EAL domain. Fold change (log2) for planktonic cells (plk) and dispersed cells obtained in response to glutamate (glu) and NO is relative to biofilms. Statistical significance was determined by analysis of variance (ANOVA). *, *P* < 0.1. (**B**) Transcript abundance of the select virulence genes in planktonic and dispersed cells relative to biofilm cells. RNA was isolated from biofilms grown for 5 days in fivefold diluted Vogel-Bonner minimal medium (VBMM), while RNA from planktonic cells was obtained from cells grown in VBMM to exponential (exp) and stationary (stat) phase. RNA for dispersed was obtained by collecting biofilm effluents post-exposure to nitric oxide. Transcript abundance of *vqsR*, *piv*, *hcn*, *rhlA*, *rpoS*, *argR*,*exsA*, *pcrV*, *pscL*, and *phzB* is determined by quantitative reverse transcription PCR (qRT-PCR), using *cysD* as housekeeper. Experiments were carried out in triplicate using biological replicates. Statistical significance was determined by ANOVA. *, *P* < 0.1; ***, *P* < 0.001; ****, *P* < 0.0001. (**C**) Abundance of pyocyanin in *P. aeruginosa* biofilm and freshly dispersed cells. Dispersed cells were obtained in response to nitric oxide. Pyocyanins were obtained by chloroform extraction. The absorbance of the bottom phase, containing pyocyanin, was determined at 690 nm. Experiments were carried out at least in triplicate using biological replicates. Statistical significance was determined by an unpaired *t*-test. **, *P* < 0.01.

Three PDEs have been associated with the modulation of c-di-GMP levels in dispersing cells, DipA, RbdA, and PA2133 (9, 55–57). Relative to biofilms, genes encoding the PDEs DipA and RbdA (*dipA, rbdA*) were found to be increased in freshly dispersed cells; however, the increase was not significant (Fig. 5A; Table S4). PA2133 was only significantly upregulated in nitric oxide-induced dispersed cells relative to biofilms (but repressed in glutamate-induced cells) (Fig. 5A). The finding suggests PA2133 plays little to no role in glutamate-induced dispersed cells and is in agreement with the inactivation of PA2133 not impairing native dispersion and dispersion induced by the dispersion inducers nitric oxide and glutamate (11). FimX (PA4959) has been linked to dispersion, although it must be noted that the PDE activity of FimX remains a somewhat contested subject (50, 58, 59). Cai et al. (56) demonstrated that the inactivation of *fimX* enhanced the dispersion of *P. aeruginosa* biofilms in response to nitric oxide relative to wild-type biofilms, likely due to the inactivation of *fimX* impairing twitching (58). Here, the *fimX* gene was found to be somewhat repressed in dispersed cells relative to biofilms (but induced relative to planktonic motility cells) (Fig. 5A). The transcriptome analysis furthermore revealed a PDE not previously linked to dispersion (Fig. 5A). MorA has been recently identified as one of two phosphodiesterases required for the maintenance of the biofilm architecture by affecting the Pel biosynthesis machinery to modulate matrix production (60).

Our study furthermore revealed the expression of two genes encoding diguanylate cyclases (GGDEF domain), *nicD* and *gcbA*, to be induced in dispersed cells (Table 1; Table S4; Fig. 5A). Both have previously been reported to play a role in sensing and relaying dispersion cue sensing, activating the central chemotaxis transducer protein BdlA (7, 55, 61). Additional genes encoding enzymes harboring GGDEF and EAL domains were identified to be differentially expressed in freshly dispersed cells as PA5442, PA2567, *pipA* (PA0285), and *proE* (PA5295). PA5442 appears to be a bifunctional enzyme (both PDE and DGC) whose function contributes to virulence, apparent by overexpression of PA5442 impairing TTSS-mediated cytotoxicity (62). The transcript abundance of PA5442 was reduced relative to biofilm and planktonic cells. Increased transcript abundance was noted for PA2567, *pipA*, and *proE* (Fig. 5A). While harboring GGDEF-EAL domains, all three proteins have been confirmed to harbor PDE activity. Moreover, ProE has been shown to negatively regulate the transcriptional expression of *pel* and *psl* (63). In contrast, PipA has been shown to contribute to autoaggregation in *P. aeruginosa* PAO1, with *pipA* inactivation coinciding with significantly increased c-di-GMP levels and aggregate formation on surfaces and in planktonic cultures (64). Freshly dispersed cells demonstrating increased expression of PA2567, *pipA*, and *proE* are in agreement with their reported function.

While the findings indicate freshly dispersed cells to differentially express genes encoding (confirmed) PDEs primarily, the difference in expression levels was subtle and not significant, suggesting that the reduced intracellular c-di-GMP levels present in freshly dispersed cells (9) are likely the result of a concerted effort of multiple c-di-GMP modulating enzymes or due to posttranslational modification/activation of phosphodiesterases, as demonstrated for DipA (55).

### Low expression levels of antibiotic resistance genes in freshly dispersed cells

Dispersed cells have been reported to be as susceptible to antibiotics as planktonic cells, particularly tobramycin and colistin (12, 14). Our comparative transcriptomic analysis revealed numerous genes previously linked to drug tolerance and drug resistance to be differentially expressed in dispersed cells relative to biofilms, with an expression pattern similar to planktonic cells. Specifically, reduced transcript abundance was noted for genes linked to tobramycin resistance, including *ndvB,* which is required for the synthesis of periplasmic glucans that physically interact with tobramycin (65), PA1874-77 encoding an ABC transporter that contributes to tobramycin tolerance of biofilms (66, 67), and *amgSR* encoding the aminoglycoside resistance-promoting envelope stress-responsive two-component system AmgSR (68). The analysis furthermore revealed reduced transcript abundance of *mex* efflux pumps, especially *mexAB-oprM* and *mexXY* in dispersed cells (Table 3).

**TABLE 3:** List of genes associated with antibiotic resistance^*a*^

Locus ID	Gene	Fold change (log2)		
		Planktonic cells	Glutamate dispersed cells	Nitric oxide dispersed cells
PA0005	lptA	−0.296291025	0.243317038	0.21516025
PA0392		−0.005826736	0.666673345	0.613207061
PA0425	mexA	−0.316823955	0.20510184	−0.281759688
PA0426	mexB	−0.216092268	0.155614565	−0.356704624
PA0427	prM	−0.207681795	0.08415034	−0.17795943
PA1163	ndvB	1.235584378	−0.257652726	0.249845224
PA1179	phoP	2.230114629	−0.56245953	−0.66719584
PA1180	phoQ	1.868689033	0.04202065	−0.131060159
PA1798	parS	0.194374377	0.053325059	0.351647974
PA1799	parR	0.56823097	−0.010711969	0.727087801
PA1874		0.420997023	0.585057701	−0.052071815
PA1875		0.001420291	0.31996359	−0.232362871
PA1876		0.379746495	0.516367584	−0.191360137
PA1877		−0.02198258	0.21519268	−0.338794973
PA1972	eptA	1.55006001	−0.497214537	0.059967207
PA2018	mexY	0.1203929	−0.038182724	−0.089579019
PA2019	mexX	0.469582994	0.559159652	0.18885821
PA2830	hptX	0.144476313	0.487968963	1.12471851
PA3077	cprR	1.09850703	0.085225015	0.667579136
PA3078	cprS	0.473165525	−0.327175072	−0.046685698
PA4119	aph	0.488838046	0.229379447	0.536709781
PA4385	groEL	−0.010271094	0.555217776	0.900314347
PA4386	groES	0.051776364	0.483918608	1.052385714
PA4776	pmrA	−0.779125698	−0.461967948	−0.448219719
PA4777	pmrB	0.850620597	−0.047504302	−0.039678898
PA4878		0.789340397	0.912070804	1.026883275
PA5199	amgS	0.526478265	0.430509993	0.473845946
PA5200	amgR	0.520362573	0.204178241	0.282544792
PA5366	pstB	−0.174470348	1.401960611	0.735851751
PA5471		0.671642476	0.24723728	0.648526105

^*a*^ Emphasis is on genes conferring resistance to tobramycin and colistin. Fold change (log2) is relative to biofilm cells.

In agreement with dispersed cells demonstrating increased susceptibility to colistin, the transcript abundance of the genes linked to lipopolysaccharide (LPS) modifications which in turn leads to colistin resistance, was reduced in dispersed cells relative to biofilms (Table 3). Most notable are genes encoding the two-component regulatory systems PhoPQ, PmrAB, ParSR, and CprSR, and their target, the *arn* LPS modification operon, that is responsible for the addition of L-aminoarabinose to LPS (69, 70). Additional LPS modification conferring resistance to colistin includes phosphoethanolamine (70). This LPS modification is conferred by the lipid A phosphoethanolamine transferase, EptA (3).

The expressions of *phoPQ*, *mexAB-oprM, mexXY,* and PA1874-77 are under the direct control of BrlR, a c-di-GMP responsive transcriptional regulator that has been linked to biofilm drug tolerance (66, 71, 72). In agreement with previous findings (73), freshly dispersed cells were characterized by the reduced expression of *brlR* (Table 3).

Our findings suggest that freshly dispersed cells adopt a more antibiotic susceptible phenotype, likely enhanced by these cells demonstrating a higher metabolic active state relative to biofilms (Fig. 4A; Table 2). However, it is important to note that dispersed cells also demonstrated higher expression of genes contributing to the reactive oxidative stress (ROS) response. These included peroxidases such as PA0848 (*ahpB*), PA0139 (*ahpC*), and PA0140 (*ahpF*), and catalases encoded by *katA*, *katB,* and *katE* (Table 1). The majority of these genes were uniquely expressed by dispersed cells relative to biofilm and planktonic cells (Table 1). Given the link between ROS and antibiotics (74), it is likely that the inverse expression patterns of genes linked to antibiotic susceptibility and ROS may contribute to dispersed cells being rendered susceptible to some but not all antibiotics (14).

### Distinct virulence gene expression profile of freshly dispersed cells

The two modes of growth, the sessile biofilm mode of growth and the planktonic, free-living mode of growth, have been associated with distinct virulence phenotypes. While acute infections are associated with the fast-growing planktonic bacteria, chronic infections have been associated with the slow-growing, aggregated bacteria known as biofilms (75, 76). In contrast, dispersed cells have been reported to be even more virulent than planktonic cells, apparent by dispersed cells, generated by overexpression of the *E. coli* phosphodiesterase YhjH, being highly virulent against macrophages and *Caenorhabditis elegans* compared with planktonic cells (12). Moreover, biofilm disassembly induced by exogenous degradation of the biofilm matrix using glycoside hydrolases was found to not only coincide with large-scale, *in vivo* dispersal of motile biofilm bacteria but to also cause lethal septicemia in the absence of antibiotic therapy in a mouse wound model (21). However, while factors contributing to the acute and chronic virulence phenotypes have been well documented, little is known about the virulence factors contributing to the distinct virulence phenotype of dispersed cells. In contrast, the inability to disperse rendered *P. aeruginosa* bdlA mutants unable to release cytotoxic and biofilm matrix degradative enzymes, but significantly enhanced chronic colonization of the murine lung compared to wild type (19).

As indicated above, among the uniquely downregulated expressed genes was *pvdR* (Table 1), with additional genes associated with the biosynthesis of the siderophores pyoverdine and pyochelin being downregulated in freshly dispersed cells relative to biofilms (Table S3). Decreased transcript abundance of *phzB* encoding phenazine biosynthesis protein PhzB in dispersed cells relative to biofilms was confirmed by quantitative reverse transcription PCR (qRT-PCR) (Fig. 5B). Also, freshly dispersed cells differentially expressed several other secreted factors. For example, dispersed cells were found to increase the expression of genes involved in hydrogen cyanide biosynthesis relative to biofilm. Increased expression of genes linked to hydrogen cyanide biosynthesis (*hcnA*, *hcnB*, *hcnC*) in dispersed cells relative to biofilms was confirmed by qRT-PCR (Fig. 5B). Freshly dispersed cells likewise induced the expression of genes involved in pyocyanin biosynthesis (Table S3). Pyocyanin has emerged as an important virulence factor, with pyocyanin more globally affecting various organ systems, including the respiratory, cardiovascular, urological, and central nervous systems (77), and damaging neutrophils, likely because pyocyanin contributes to the generation of reactive oxygen species. Biochemical analysis confirmed dispersed cells to produce pyocyanin, an exuberant blue-colored phenazine, in greater abundance than biofilms (Fig. 5C).

Freshly dispersed cells also exhibited differential expression of genes related to secretion systems (Table S3). Specifically, dispersed cells demonstrated reduced transcript abundance of type III and type VI secretion systems. Reduced transcript abundance of genes encoding the type III secretion system (*popN*, *pscP, pscR, pcrV*, *pscL, pscH, pscD, pscH*), the effector protein (*exoT*), and the transcriptional activator of the type III secretion system, ExsA, was confirmed by qRT-PCR (Fig. 5B). In contrast to type III and VI pathways, the type II secretion system drives exclusively translocation of effector proteins across the outer membrane (78), in a manner dependent on either the Sec or Tat transport systems (79) The Xcp system is required for secretion of exotoxin A, lipases, phospholipases C, alkaline phosphatase, or elastase (LasB) (80), and the Hxc system is required for the secretion of the low-molecular-weight alkaline phosphatase LapA (81). Freshly dispersed cells exhibited an overall reduced transcript abundance of genes associated with the Xcp system relative to biofilms but an increased transcript abundance of genes associated with the Hxc type II secretion system, with the expression patterns mirroring that of planktonic cells (Table S3).

Both type III and VI secretion systems enable the direct injection of virulence factors into the host cell cytoplasm (82, 83), while the type III secretion system is responsible for killing macrophages (84). The findings suggest that dispersed cells switch from intoxicating host cells and potential competitors to protecting them from circulating immune cells. This is supported by the induced expression of *lapA*, with modulation of LapA having been shown to affect elastase activity, swimming motility, C4-HSL, and 3-oxo-C12-HSL production, rhamnolipid production as well as biofilm formation and virulence by *P. aeruginosa* PAO1 (85). Further evidence of dispersed cells undergoing a switch in virulence (and being well protected from opsonizing immune cells) stems from this cell population, demonstrating increased transcript abundance of genes involved in the synthesis of LPS and the LPS O antigen relative to biofilm cells (Table S3). The LPS O antigen has been shown to play a crucial role in providing serum resistance by preventing the formation of convertase and blocking the C3b-factor B binding sites, thus protecting bacteria from engulfment by prolonging the onset of immune receptors (86). However, the contribution of LPS in virulence is less clear. While LPS has been reported as a prominent factor in mediating both bacterial virulence and host responses by providing protection from macrophages, LPS (87, 88) has also been reported to be immunostimulatory, with the immunomodulatory effect largely dependent on the structure of the LPS (88–90).

Additional virulence genes encoding degradative enzymes, including proteases and lipases, quinolone, cell-to-cell signaling, and regulators, all previously linked to virulence, were differentially expressed in dispersed cells and included *plcB*, *pldA*, *lasA, rhlA*, *lptA*, *pqsA*, *pqsB, pqsC*, *mvfR,* PA0684, PA0685, *vqsR*, *piv*, and *argR* (Table S3). Differential expression of select virulence genes (*vqsR*, *rhlA*, and *argR*) was confirmed by qRT-PCR (Fig. 5B).

Our findings suggested that freshly dispersed cells differ from biofilm and planktonic cells by the reduced expression of genes linked to iron acquisition and effector proteins that are delivered by direct injection while increasing the expression of genes associated with macrophage killing and evasion. The latter is accomplished by freshly dispersed cells preferentially expressing type II secretion system over type III and VI.

### Evasion of freshly dispersed cells from cultured macrophages

**Fig 6:**
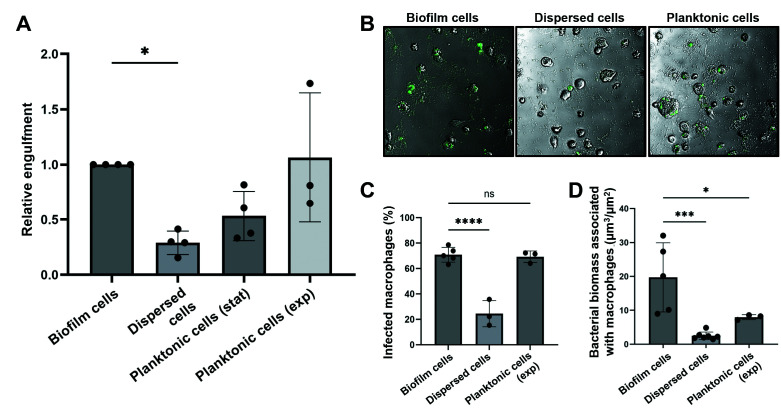
Phagocytosis evasion by biofilm, planktonic, and freshly dispersed cells by *P. aeruginosa* PAO1. Macrophage infection assay-The monocyte cell line THP-1 was differentiated for 3 days, adding 100 nM phorbol-12-myristate-13-acetate. The differentiated macrophages were infected with biofilm cells, planktonic cells grown to exponential (exp) and stationary phase (stat), and dispersed cells obtained in response to nitric oxide, at an MOI of 1:10 for 90 minutes. (**A**) After washing and removing extracellular bacteria by adding gentamicin, macrophages were lysed, and engulfed bacteria were quantified by drop plating. Statistical significance was determined by analysis of variance (ANOVA). *, *P* < 0.1. (**B**) Representative confocal microscopic images of macrophages following infection for 90 minutes with constitutively gfp-expressing *P. aeruginosa* cells grown as biofilms, planktonic cells, and dispersed cells obtained in response to nitric oxide. Infected macrophages were washed and treated with gentamicin to remove extracellular bacteria. Images are shown as overlays (brightfield, fluorescence). (**C**) Percentage of differentiated macrophages infected with biofilm cells, planktonic cells, and dispersed cells obtained in response to nitric oxide. Quantitative analysis based on confocal images. Statistical significance was determined by ANOVA. ****, *P* < 0.0001; ns, not significant. (**D**) Quantitative analysis of the bacterial load associated with differentiated macrophages infected with biofilm cells, planktonic cells, and dispersed cells obtained in response to nitric oxide. Statistical significance was determined by ANOVA. *, *P* < 0.1; ***, *P* < 0.001. Controls for data shown in (**C**) and (**D**) are shown in Fig. S2.

Considering the indication of freshly dispersed cells likely avoiding engulfment by macrophages more efficiently than biofilm or planktonic cells, we next asked if dispersed cells are more protected from macrophages than planktonic or biofilm cells. We, therefore, subjected dispersed cells to macrophages, followed by quantitation of intracellular *P. aeruginosa* cells after phagocytosis. Macrophages were infected with wild-type PAO1 cells grown as biofilm and planktonically (exponential and stationary phase), and dispersed cells obtained in response to nitric oxide were used. The respective cells were adjusted to 5 × 10^7^ cells/mL and exposed to 5 × 10^6^ macrophages at a multiplicity of infection (MOI) of 1:10. After 90 minutes of phagocytosis, the number of engulfed bacteria per macrophage was quantified using viability counts. Among cells representing the different modes of growth (sessile, planktonic, dispersed), macrophages infected with planktonic cells grown to exponential phase harbored the highest number of intracellular cells (Fig. 6A). In contrast, macrophages infected with dispersed cells were found to harbor the least number of intracellular bacteria (Fig. 6A). On average, only 10 dispersed cells were detected per 100 macrophages. This contrasts with planktonic cells grown to stationary phase for which, on average, 35 intracellular cells per 100 macrophages were detected, and those grown to exponential phase cells for which 48 cells per 100 macrophages were recovered after lysis of the macrophages (Fig. 6A). Overall, the number of engulfed dispersed cells was significantly reduced relative to macrophages infected with biofilm and planktonic cells (Fig. 6A).

To determine whether the reduced engulfment of dispersed cells was due to avoidance of phagocytic uptake, we next determined the number of macrophages having engulfed bacterial cells post 90 minutes of incubation. This time was chosen as this exposure of macrophages to *P. aeruginosa* for 90 minutes has previously been shown to be insufficient to effectively eliminate bacterial cells (91). We made use of confocal microscopy and green fluorescent protein (GFP)-labeled *P. aeruginosa* grown as biofilm, planktonically (exponential phase), and dispersed cells obtained in response to nitric oxide to visualize engulfment. Visual analysis of confocal microscope images indicated the majority of macrophages exposed to biofilm and planktonic cells to harbor engulfed cells, while very few infected macrophages harboring dispersed cells were noted (Fig. 6B). We also noticed debris likely indicative of lysed macrophages, particularly in samples exposed to biofilm and planktonic cells (Fig. 6B). The visual observation of reduced engulfment of dispersed cells was supported by quantitative analysis (Fig. 6C). While approximately 70% of macrophages were found to harbor biofilm and planktonic cells, less than 30% of macrophages exposed to dispersed cells harbored engulfed cells (Fig. 6C). Quantitative analysis of the engulfed bacterial biomass by COMSTAT further indicated significant differences in the number of engulfed cells, with the highest bacterial numbers or biomass detected for macrophages exposed to biofilm cells, and the least for those exposed to dispersed cells (Fig. 6D). The difference was not due to differences in the number of macrophages, as no difference in the ratio of surface coverage to biovolume was noted (Fig. S2).

Our findings thus indicate freshly dispersed cells significantly evade macrophages compared to biofilm and planktonic cells, strongly suggesting immune evasion not only to be a trait of freshly dispersed cells that is induced early but also significantly contributing to the enhanced virulence phenotype relative to planktonic cells. Immune evasion may be due to preferential expression of the type II secretion system over type III and VI by freshly dispersed cells, LPS and LPS O antigens, and/or increased expression of ROS response genes. A similar immune evasion trait was noted for dispersed cells 5 hours post dispersion (12). The findings strongly suggest immune evasion of dispersed cells to be a long-lasting trait.

### Conclusion

**Fig 7:**
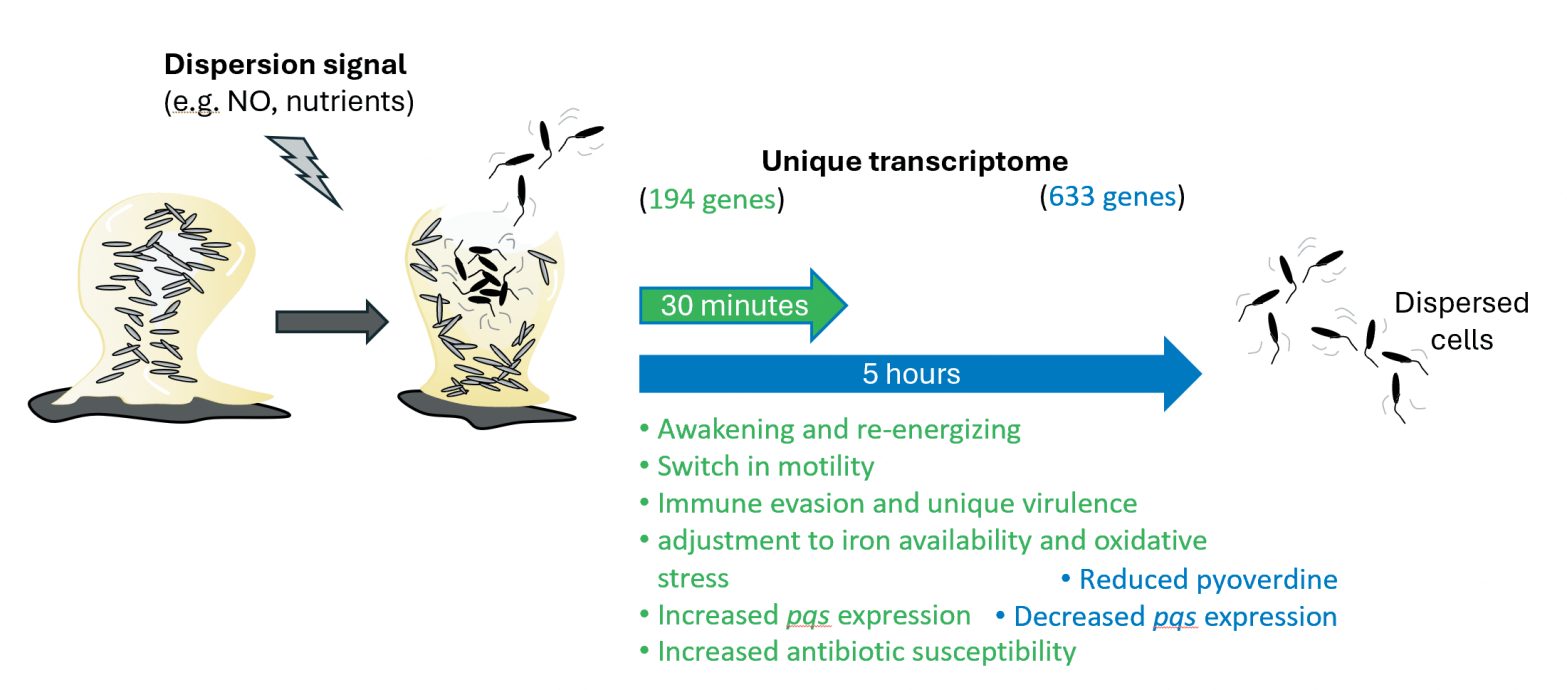
Summary of major traits of freshly dispersed cells based on phenotypic characterization and the unique and differential expression of genes relative to biofilm and planktonic cells. Traits in green specifically refer to freshly dispersed cells but also apply to dispersed cells post 5 hours of dispersion. Traits in blue are unique to 5-hour-old dispersed cells.

Biofilm dispersion is an evasion strategy to escape from the overcrowded biofilm, move to the new site, and establish a new biofilm there (92). Biofilms can disperse in response to various signals, including nitric oxide, nutrient availability (starvation or abundance), increased pH, oxygen limitation, and the fatty acid signaling molecule *cis*-2-decenoic acid that has been shown to have cross-kingdom dispersion-inducing capabilities (3, 92). The goal of this study was to determine the dispersion transcriptome and the phenotypic traits of dispersed cells to better understand what makes them so distinct from planktonic and biofilm cells. Our study demonstrates dispersed cells obtained within 30 minutes in response to exposure to nitric oxide and glutamate to be distinct from biofilm and planktonic cells (Fig. 1). These freshly dispersed cells uniquely express 194 genes suggestive of dispersed cells experiencing a switch in motility, as well as increased protein synthesis, metabolic activities, and energy generation while adjusting to iron availability and oxidative stress (Table 1; Fig. 7). When considering genes differentially expressed to biofilms or planktonic cells, the expanded dispersion transcriptome indicated freshly dispersed cells to be distinguished from biofilms or planktonic cells by signaling, c-di-GMP modulation, antibiotic resistance, and virulence (Fig. 7). With respect to virulence, freshly dispersed cells were shown to evade phagocytosis more significantly than biofilm and planktonic cells (Fig. 6). Moreover, by comparing our findings with those obtained using *P. aeruginosa* dispersed cells post 5 hours of induction of dispersion (12), it is apparent that the transcriptome of dispersed cells expands, with differences in gene expression becoming more pronounced (Fig. 2), and dispersed cells likely maintaining their dispersed state for an extended period of time, including their heightened immune evasion capabilities for hours before reverting to the planktonic mode (Fig. 7). It is thus likely that dispersed cells can likewise remain highly virulent within the host before re-colonizing a new infection site. Therefore, potential biofilm dispersal strategies should be combined with, for example, antimicrobial strategies capable of killing dispersed cells to eliminate the threat dispersed cells represent to the immune system.

## MATERIALS AND METHODS

### Bacterial strain, media, and growth conditions

*Pseudomonas aeruginosa* strain PAO1 was used in this study. Where indicated, a constitutively expressing GFP strain, PAO1::miniTn7-gfp, was used. All planktonic cultures were grown in Vogel-Bonner minimal medium (VBMM) (4) in flasks at 220 rpm and 37°C. Fresh planktonic cultures were started from a 1% inoculum of an overnight culture grown from a frozen stock. The resulting suspension was briefly spun down (16,000 × *g*, 1 min), resuspended in fresh VBMM, and diluted to a final OD_600_ of 0.2 using fresh VBMM. The suspension was subsequently grown for up to 8 hours to exponential phase at 22°C as previously reported (14). Biofilms were grown as indicated below.

### Biofilm formation and dispersion assays

*P. aeruginosa* PAO1 biofilms were grown using a continuous flow tube reactor system consisting of a 1-m silicone tubing (size 14; Masterflex; Cole Parmer, Inc.). The biofilms were grown in fivefold-diluted VBMM at a temperature of 22°C and a flow rate of 0.2 mL/min, following previously established protocols (14). Briefly, after 5 days of biofilm growth, dispersion was induced by a sudden switch to a growth medium containing L-glutamate (18 mM) or 500 µM sodium nitroprusside, as described in previous studies (11, 93). Sodium nitroprusside was utilized as a source of NO. Regardless of the dispersion-inducing conditions, dispersed cells were collected from the effluent of the tube reactor at 1-minute intervals. The biofilm effluent optical density (OD) was measured using spectrophotometry. Dispersion events were identified by an increase in turbidity observed at 600 nm compared to the effluents from biofilms that were not exposed to dispersion cues (14).

### RNA-seq library construction and sequencing

To isolate RNA from dispersed cells, the dispersed cells were directly collected into equal volumes of RNAprotect (Qiagen). The respective strains were initially grown in VBMM to isolate RNA from planktonic cells. Subsequently, approximately 6 mL of the bacterial culture was harvested by centrifugation (10,000 × *g*, 5 minutes at 4°C) and resuspended in 1 mL of RNA protect. The mRNA isolation process followed a previously described protocol.

To remove rRNA, the Ribo-Zero rRNA Removal kit for Gram-negative bacteria (Illumina, San Diego, CA) was utilized according to the manufacturer’s instructions. The quality and concentration of the isolated RNA were assessed by measuring absorbance and calculating the A260/A280 and A260/A230 ratios using a NanoDrop One Microvolume UV spectrophotometer (Thermo Scientific, MA, USA). The RNA integrity was evaluated using an Agilent 2100 Bioanalyzer (Agilent Technologies, CA, USA), and only samples with an RNA integrity number of ≥9 were used for subsequent analysis.

For library construction, the Ion Total RNA-Seq kit v2 (Life Technologies, Carlsbad, CA) was employed, and the libraries were evaluated using a Qubit 2 Fluorometer (Thermo Scientific, MA, USA). Finally, the libraries were sequenced using the ION Torrent Personal Genome System (Life Technologies, Carlsbad, CA).

### Transcriptomic analysis

To process and analyze the raw sequencing reads, the FASTQC software was utilized to perform quality assessment and cleaning. Reads containing adapter sequences and low-quality bases (Q < 20) were removed. Additionally, the Trimmomatic software was employed to eliminate rRNA reads. The resulting cleaned reads were then aligned to the reference genome of the *P. aeruginosa* PAO1 strain (Taxonomy ID: 208964) using bowtie2. For downstream analysis, samtools and featurecounts were used to count the aligned reads with respect to genomic features (94). Differential gene expression analysis was performed using the DESeq2 package (Bioconductor) (95), employing a negative binomial distribution in the R software. Genes exhibiting an adjusted *P*-value (*P*_adj_) <0.05 and an absolute log2 fold change >1 were considered as DEGs. PCA, heatmap, and volcano plots were generated using the pheatmap and ggplot2 packages to visualize and explore the data. Moreover, GO enrichment and gene set enrichment analysis were conducted using the clusterProfiler package to identify the biological functions and pathways primarily affected by the DEGs (96).

### qRT-PCR

To validate our findings, we performed qRT-PCR to assess the expression of virulence genes, namely *vqsR*, *piv*, *hcn*, *rhlA*, *exsA*, *argR*, *pcrV*, *pscL* and *phzB* using the Bio-Rad CFX Connect Real-Time PCR Detection System (Bio-Rad) and SsoAdvanced SYBR Green Supermix (Bio-Rad). We used *cysD* as a control and normalized the transcript abundance based on the threshold cycle value [Ct]. We determined transcript abundance ratios and verified specific single-product amplification using melting curve analyses. Primers used in this study are listed in Table S5.

### CTC staining

CTC is a monotetrazolium redox dye that generates a fluorescent formazan (CTC-formazan, CTF) that gives off a red fluorescence upon biological reduction, indicating metabolic activity. The metabolic activity of dispersed cells, biofilm cells, planktonic exponential and stationary cells was assessed by utilizing the BacLight RedoxSensor CTC Vitality Kit (Invitrogen). Syto24 staining was used as a counter stain to account for total cells. All the cells were washed, OD adjusted to 0.2 and incubated with CTC (50 mM) and Syto24 (10 nM) for 30 minutes at 37°C, protected from light. Cells without any staining were used as unstained bacterial control. After 30 minutes, the fluorescence of CTC (excitation: 450 nm, emission: 630 nm) and Syto24 (excitation: 490 nm, emission: 515 nm) was measured using a plate reader (Spectramax i3x, Molecular Devices).

### Macrophage engulfment assay

THP-1 monocytes were cultured in RPMI-1640 with 10% fetal bovine serum (FBS) for 3–5 days until they reach a concentration of 8 × 10^5^ cells/mL. After this, 5 × 10^5^ cells/mL THP-1 were activated with 100 nM phorbol-12-myristate-13-acetate for 3 days at 37°C in 5% CO_2_ (97). Dispersed cells were pooled and washed to remove any dispersion inducer and resuspended in infection media to 5 × 10^6^ cells/mL and added to the differentiated THP-1 cells at a multiplicity of infection (MOI) of 10:1. After a 90-minute incubation, cells were washed with phosphate buffered saline (PBS) to remove extracellular bacteria. Cells were treated with gentamicin (40 mg/L) to remove extracellular bacteria. Macrophages were subsequently lysed with 10% Triton-X 100 to release engulfed bacteria quantified by plating (98). Additionally, macrophages post 90-minute incubation and exposure to gentamicin were viewed by confocal microscopy. Confocal images were used to determine the percent of infected macrophages, and the engulfed bacterial biomass was quantitatively analyzed using COMSTAT (99). For quantification, at least six random confocal images per replicate were used.

### Pyocyanin and pyoverdine

As previously described, pyocyanin and pyoverdine concentrations were determined (100–102) Briefly, OD-adjusted biofilm and dispersed cells were pelleted (7,500× rpm, 10 minutes), and the supernatant was added to equal parts of chloroform in a glass test tube. Samples were mixed well and rested at 22°C for 5 minutes. The top layer containing pyoverdine was removed and placed in a separate tube. The pyoverdine-containing solution was diluted and plated (200 µL) into a black flat-bottom 96-well polystyrene plate. The fluorescence (excitation: 460 nm, emission: 405 nm) was measured using a plate reader (Spectramax i3x, Molecular Devices). The bottom layer containing pyocyanin was measured using a plate reader (Spectramax i3x) at an absorbance of 690 nm.

### Statistics

For pairwise comparison, a two-tailed Student’s *t*-test assuming equal variance or using single-factor analysis of variance (ANOVA) was used. In addition, statistical differences between strains and/or conditions were determined using a one-way ANOVA, followed by a Dunnett’s *post hoc* test using Prism5 software (GraphPad, La Jolla, CA, USA). Unless otherwise noted, all experiments were performed at least in triplicate using biological replicates. Statistics for differential gene expression and generation of plots is performed using R language, which further calculated values for fold change, *P*-value, and adjusted *P*-value.

## Data Availability

All data will be made fully available and without restriction upon request.

## SUPPLEMENTAL MATERIAL

Supplemental Figures msphere.00884-24-s0001.pdf Figures S1 and S2.

Legends msphere.00884-24-s0002.pdf Supplemental table and figure legends.

Table S1 msphere.00884-24-s0003.pdf Dispersion transcriptome.

Table S2 msphere.00884-24-s0004.docx Transcript abundance of genes linked to motility and exopolysaccharide biosynthesis.

Table S3 msphere.00884-24-s0005.docx Transcript abundance of genes linked to virulence.

Table S4 msphere.00884-24-s0006.docx Differential expression of genes encoding c-di-GMP modulating enzymes.

Table S5 msphere.00884-24-s0007.docx Primers.

ASM does not own the copyrights to Supplemental Material that may be linked to, or accessed through, an article. The authors have granted ASM a non-exclusive, world-wide license to publish the Supplemental Material files. Please contact the corresponding author directly for reuse.

## References

[1] Barraud N, Schleheck D, Klebensberger J, Webb JS, Hassett DJ, Rice SA, Kjelleberg S. 2009. Nitric oxide signaling in Pseudomonas aeruginosa biofilms mediates phosphodiesterase activity, decreased cyclic di-GMP levels, and enhanced dispersal. J Bacteriol 191:7333–7342. doi:10.1128/JB.00975-09 19801410 PMC2786556

[2] Huynh TT, McDougald D, Klebensberger J, Al Qarni B, Barraud N, Rice SA, Kjelleberg S, Schleheck D. 2012. Glucose starvation-induced dispersal of Pseudomonas aeruginosa biofilms is cAMP and energy dependent. PLoS ONE 7:e42874. doi:10.1371/journal.pone.0042874 22905180 PMC3419228

[3] Davies DG, Marques CNH. 2009. A fatty acid messenger is responsible for inducing dispersion in microbial biofilms. J Bacteriol 191:1393–1403. doi:10.1128/JB.01214-08 19074399 PMC2648214

[4] Sauer K, Cullen MC, Rickard AH, Zeef LAH, Davies DG, Gilbert P. 2004. Characterization of nutrient-induced dispersion in Pseudomonas aeruginosa PAO1 biofilm. J Bacteriol 186:7312–7326. doi:10.1128/JB.186.21.7312-7326.2004 15489443 PMC523207

[5] Kalia M, Amari D, Davies DG, Sauer K. 2023. Cis-DA-dependent dispersion by Pseudomonas aeruginosa biofilm and identification of cis-DA-sensory protein DspS:mBio. doi:10.1128/mbio.02570-23Abstract PMC10746223 38014955

[6] Petrova OE, Sauer K. 2012. PAS domain residues and prosthetic group involved in bdlA-dependent dispersion response by Pseudomonas aeruginosa biofilms. J Bacteriol 194:5817–5828. doi:10.1128/JB.00780-12 22923587 PMC3486124

[7] Petrova OE, Sauer K. 2012. Dispersion by Pseudomonas aeruginosa requires an unusual posttranslational modification of BdlA. Proc Natl Acad Sci U S A 109:16690–16695. doi:10.1073/pnas.1207832109 23012421 PMC3478618

[8] Morgan R, Kohn S, Hwang SH, Hassett DJ, Sauer K. 2006. BdlA, a chemotaxis regulator essential for biofilm dispersion in Pseudomonas aeruginosa. J Bacteriol 188:7335–7343. doi:10.1128/JB.00599-06 17050921 PMC1636253

[9] Roy AB, Petrova OE, Sauer K. 2012. The phosphodiesterase DipA (PA5017) is essential for Pseudomonas aeruginosa biofilm dispersion. J Bacteriol 194:2904–2915. doi:10.1128/JB.05346-11 22493016 PMC3370607

[10] Jones CJ, Newsom D, Kelly B, Irie Y, Jennings LK, Xu B, Limoli DH, Harrison JJ, Parsek MR, White P, Wozniak DJ. 2014. ChIP-Seq and RNA-Seq reveal an AmrZ-mediated mechanism for cyclic di-GMP synthesis and biofilm development by Pseudomonas aeruginosa. PLoS Pathog 10:e1003984. doi:10.1371/journal.ppat.1003984 24603766 PMC3946381

[11] Kalia M, Resch MD, Cherny KE, Sauer K. 2022. The alginate and motility regulator AmrZ is essential for the regulation of the dispersion response by Pseudomonas aeruginosa biofilms . mSphere 7. doi:10.1128/msphere.00505-22 PMC9769550 36374041

[12] Chua SL, Liu Y, Yam JKH, Chen Y, Vejborg RM, Tan BGC, Kjelleberg S, Tolker-Nielsen T, Givskov M, Yang L. 2014. Dispersed cells represent a distinct stage in the transition from bacterial biofilm to planktonic lifestyles. Nat Commun 5:4462. doi:10.1038/ncomms5462 25042103

[13] Vaysse PJ, Sivadon P, Goulas P, Grimaud R. 2011. Cells dispersed from Marinobacter hydrocarbonoclasticus SP17 biofilm exhibit a specific protein profile associated with a higher ability to reinitiate biofilm development at the hexadecane-water interface. Environ Microbiol 13:737–746. doi:10.1111/j.1462-2920.2010.02377.x 21087383

[14] Chambers JR, Cherny KE, Sauer K. 2017. Susceptibility of Pseudomonas aeruginosa dispersed cells to antimicrobial agents Is dependent on the dispersion cue and class of the antimicrobial agent used. Antimicrob Agents Chemother 61:e00846-17. doi:10.1128/AAC.00846-17 28971863 PMC5700346

[15] Cherny KE, Sauer K. 2020. Untethering and degradation of the polysaccharide matrix are essential steps in the dispersion response of Pseudomonas aeruginosa biofilms. J Bacteriol 202:e00575-19. doi:10.1128/JB.00575-19 31712279 PMC6964737

[16] Cherny KE, Sauer K. 2019. Pseudomonas aeruginosa requires the DNA-specific endonuclease enda to degrade extracellular genomic DNA to disperse from the biofilm. J Bacteriol 201:e00059-19. doi:10.1128/JB.00059-19 30988033 PMC6707924

[17] Rollet C, Gal L, Guzzo J. 2009. Biofilm-detached cells, a transition from a sessile to a planktonic phenotype: a comparative study of adhesion and physiological characteristics in Pseudomonas aeruginosa. FEMS Microbiol Lett 290:135–142. doi:10.1111/j.1574-6968.2008.01415.x 19054076

[18] Wille J, Teirlinck E, Sass A, Van Nieuwerburgh F, Kaever V, Braeckmans K, Coenye T. 2020. Does the mode of dispersion determine the properties of dispersed Pseudomonas aeruginosa biofilm cells? Int J Antimicrob Agents 56:106194. doi:10.1016/j.ijantimicag.2020.106194 33039591

[19] Li Y, Petrova OE, Su S, Lau GW, Panmanee W, Na R, Hassett DJ, Davies DG, Sauer K. 2014. BdlA, DipA and induced dispersion contribute to acute virulence and chronic persistence of Pseudomonas aeruginosa. PLoS Pathog 10:e1004168. doi:10.1371/journal.ppat.1004168 24901523 PMC4047105

[20] Guilhen C, Miquel S, Charbonnel N, Joseph L, Carrier G, Forestier C, Balestrino D. 2019. Colonization and immune modulation properties of Klebsiella pneumoniae biofilm-dispersed cells. NPJ Biofilms Microbiomes 5:25. doi:10.1038/s41522-019-0098-1 31583108 PMC6760147

[21] Fleming D, Rumbaugh K. 2018. The consequences of biofilm dispersal on the host. Sci Rep 8:10738. doi:10.1038/s41598-018-29121-2 30013112 PMC6048044

[22] Sauer K, Camper AK, Ehrlich GD, Costerton JW, Davies DG. 2002. Pseudomonas aeruginosa displays multiple phenotypes during development as a biofilm. J Bacteriol 184:1140–1154. doi:10.1128/jb.184.4.1140-1154.2002 11807075 PMC134825

[23] Leggett A, Li DW, Bruschweiler-Li L, Sullivan A, Stoodley P, Brüschweiler R. 2022. Differential metabolism between biofilm and suspended Pseudomonas aeruginosa cultures in bovine synovial fluid by 2D NMR-based metabolomics. Sci Rep 12:17317. doi:10.1038/s41598-022-22127-x 36243882 PMC9569359

[24] Spindler EC, Hale JDF, Giddings TH, Hancock REW, Gill RT. 2011. Deciphering the mode of action of the synthetic antimicrobial peptide Bac8c. Antimicrob Agents Chemother 55:1706–1716. doi:10.1128/AAC.01053-10 21282431 PMC3067151

[25] Yu Z, Cai Y, Qin W, Lin J, Qiu J. 2015. Polymyxin e induces rapid paenibacillus polymyxa death by damaging cell membrane while Ca2+ can protect cells from damage. PLoS ONE 10:e0135198. doi:10.1371/journal.pone.0135198 26252512 PMC4529208

[26] Park YK, Ko KS. 2015. Effect of carbonyl cyanide 3-chlorophenylhydrazone (CCCP) on killing Acinetobacter baumannii by colistin. J Microbiol 53:53–59. doi:10.1007/s12275-015-4498-5 25557480

[27] Mohamed YF, Abou-Shleib HM, Khalil AM, El-Guink NM, El-Nakeeb MA. 2016. Membrane permeabilization of colistin toward pan-drug resistant gram-negative isolates. Braz J Microbiol 47:381–388. doi:10.1016/j.bjm.2016.01.007 26991296 PMC4874589

[28] Ni W, Li Y, Guan J, Zhao J, Cui J, Wang R, Liu Y. 2016. Effects of efflux pump inhibitors on colistin resistance in multidrug-resistant gram-negative bacteria. Antimicrob Agents Chemother 60:3215–3218. doi:10.1128/AAC.00248-16 26953203 PMC4862505

[29] Jüngst A, Zumft WG. 1992. Interdependence of respiratory NO reduction and nitrite reduction revealed by mutagenesis of nirQ, a novel gene in the denitrification gene cluster of Pseudomonas stutzeri. FEBS Lett 314:308–314. doi:10.1016/0014-5793(92)81495-8 1468562

[30] Thauer RK, Jungermann K, Decker K. 1977. Energy conservation in chemotrophic anaerobic bacteria. Bacteriol Rev 41:100–180. doi:10.1128/br.41.1.100-180.1977 860983 PMC413997

[31] Créach V, Baudoux AC, Bertru G, Rouzic BL. 2003. Direct estimate of active bacteria: CTC use and limitations. J Microbiol Methods 52:19–28. doi:10.1016/s0167-7012(02)00128-8 12401223

[32] Smith JJ, McFeters GA. 1997. Mechanisms of INT (2-(4-iodophenyl)-3-(4-nitrophenyl)-5-phenyl tetrazolium chloride), and CTC (5-cyano-2,3-ditolyl tetrazolium chloride) reduction in Escherichia coli K-12. J Microbiol Methods 29:161–175. doi:10.1016/S0167-7012(97)00036-5 8642015

[33] Dasgupta N, Wolfgang MC, Goodman AL, Arora SK, Jyot J, Lory S, Ramphal R. 2003. A four-tiered transcriptional regulatory circuit controls flagellar biogenesis in Pseudomonas aeruginosa. Mol Microbiol 50:809–824. doi:10.1046/j.1365-2958.2003.03740.x 14617143

[34] Bouteiller M, Dupont C, Bourigault Y, Latour X, Barbey C, Konto-Ghiorghi Y, Merieau A. 2021. Pseudomonas flagella: generalities and specificities. Int J Mol Sci 22:3337. doi:10.3390/ijms22073337 33805191 PMC8036289

[35] Wang S, Yu S, Zhang Z, Wei Q, Yan L, Ai G, Liu H, Ma LZ. 2014. Coordination of swarming motility, biosurfactant synthesis, and biofilm matrix exopolysaccharide production in Pseudomonas aeruginosa. Appl Environ Microbiol 80:6724–6732. doi:10.1128/AEM.01237-14 25172852 PMC4249032

[36] Jennings LK, Storek KM, Ledvina HE, Coulon C, Marmont LS, Sadovskaya I, Secor PR, Tseng BS, Scian M, Filloux A, Wozniak DJ, Howell PL, Parsek MR. 2015. Pel is a cationic exopolysaccharide that cross-links extracellular DNA in the Pseudomonas aeruginosa biofilm matrix. Proc Natl Acad Sci U S A 112:11353–11358. doi:10.1073/pnas.1503058112 26311845 PMC4568648

[37] Ma L, Conover M, Lu H, Parsek MR, Bayles K, Wozniak DJ. 2009. Assembly and development of the Pseudomonas aeruginosa biofilm matrix. PLoS Pathog 5:e1000354. doi:10.1371/journal.ppat.1000354 19325879 PMC2654510

[38] Evans LR, Linker A. 1973. Production and characterization of the slime polysaccharide of Pseudomonas aeruginosa. J Bacteriol 116:915–924. doi:10.1128/jb.116.2.915-924.1973 4200860 PMC285463

[39] Marmont LS, Whitfield GB, Rich JD, Yip P, Giesbrecht LB, Stremick CA, Whitney JC, Parsek MR, Harrison JJ, Howell PL. 2017. PelA and PelB proteins form a modification and secretion complex essential for Pel polysaccharide-dependent biofilm formation in Pseudomonas aeruginosa J Biol Chem 292:19411–19422. doi:10.1074/jbc.M117.812842 28972168 PMC5702679

[40] Baker P, Hill PJ, Snarr BD, Alnabelseya N, Pestrak MJ, Lee MJ, Jennings LK, Tam J, Melnyk RA, Parsek MR, Sheppard DC, Wozniak DJ, Howell PL. 2016. Exopolysaccharide biosynthetic glycoside hydrolases can be utilized to disrupt and prevent Pseudomonas aeruginosa biofilms. Sci Adv 2:e1501632. doi:10.1126/sciadv.1501632 27386527 PMC4928890

[41] Cooke AC, Florez C, Dunshee EB, Lieber AD, Terry ML, Light CJ, Schertzer JW. 2020. Pseudomonas quinolone signal-induced outer membrane vesicles enhance biofilm dispersion in Pseudomonas aeruginosa. mSphere 5:mSphere doi:10.1128/mSphere.01109-20 PMC7690959 33239369

[42] Valentini M, Filloux A. 2016. Biofilms and cyclic di-GMP (c-di-GMP) signaling: lessons from Pseudomonas aeruginosa and other bacteria. J Biol Chem 291:12547–12555. doi:10.1074/jbc.R115.711507 27129226 PMC4933438

[43] Jenal U, Reinders A, Lori C. 2017. Cyclic di-GMP: second messenger extraordinaire. Nat Rev Microbiol 15:271–284. doi:10.1038/nrmicro.2016.190 28163311

[44] Römling U, Galperin MY, Gomelsky M. 2013. Cyclic di-GMP: the first 25 years of a universal bacterial second messenger. Microbiol Mol Biol Rev 77:1–52. doi:10.1128/MMBR.00043-12 23471616 PMC3591986

[45] Colvin KM, Irie Y, Tart CS, Urbano R, Whitney JC, Ryder C, Howell PL, Wozniak DJ, Parsek MR. 2012. The Pel and Psl polysaccharides provide Pseudomonas aeruginosa structural redundancy within the biofilm matrix. Environ Microbiol 14:1913–1928. doi:10.1111/j.1462-2920.2011.02657.x 22176658 PMC3840794

[46] Berne C, Ellison CK, Ducret A, Brun YV. 2018. Bacterial adhesion at the single-cell level. Nat Rev Microbiol 16:616–627. doi:10.1038/s41579-018-0057-5 30008468

[47] Ha D-G, O’Toole GA. 2015. C-di-GMP and its effects on biofilm formation and dispersion: a Pseudomonas Aeruginosa review. Microbiol Spectr 3:MB–0003 doi:10.1128/microbiolspec.MB-0003-2014 PMC4498269 26104694

[48] Simm R, Morr M, Kader A, Nimtz M, Römling U. 2004. GGDEF and EAL domains inversely regulate cyclic di-GMP levels and transition from sessility to motility. Mol Microbiol 53:1123–1134. doi:10.1111/j.1365-2958.2004.04206.x 15306016

[49] Ryjenkov DA, Tarutina M, Moskvin OV, Gomelsky M. 2005. Cyclic diguanylate is a ubiquitous signaling molecule in bacteria: insights into biochemistry of the GGDEF protein domain. J Bacteriol 187:1792–1798. doi:10.1128/JB.187.5.1792-1798.2005 15716451 PMC1064016

[50] Chan C, Paul R, Samoray D, Amiot NC, Giese B, Jenal U, Schirmer T. 2004. Structural basis of activity and allosteric control of diguanylate cyclase. Proc Natl Acad Sci U S A 101:17084–17089. doi:10.1073/pnas.0406134101 15569936 PMC535365

[51] Hengge R. 2009. Principles of c-di-GMP signalling in bacteria. Nat Rev Microbiol 7:263–273. doi:10.1038/nrmicro2109 19287449

[52] Cohen D, Mechold U, Nevenzal H, Yarmiyhu Y, Randall TE, Bay DC, Rich JD, Parsek MR, Kaever V, Harrison JJ, Banin E. 2015. Oligoribonuclease is a central feature of cyclic diguanylate signaling in Pseudomonas aeruginosa. Proc Natl Acad Sci U S A 112:11359–11364. doi:10.1073/pnas.1421450112 26305928 PMC4568660

[53] Orr MW, Donaldson GP, Severin GB, Wang J, Sintim HO, Waters CM, Lee VT. 2015. Oligoribonuclease is the primary degradative enzyme for pGpG in Pseudomonas aeruginosa that is required for cyclic-di-GMP turnover. Proc Natl Acad Sci U S A 112:E5048–57. doi:10.1073/pnas.1507245112 26305945 PMC4568665

[54] Dow JM, Fouhy Y, Lucey JF, Ryan RP. 2006. The HD-GYP domain, cyclic di-GMP signaling, and bacterial virulence to plants. Mol Plant Microbe Interact 19:1378–1384. doi:10.1094/MPMI-19-1378 17153922

[55] Basu Roy A, Sauer K. 2014. Diguanylate cyclase NicD-based signalling mechanism of nutrient-induced dispersion by Pseudomonas aeruginosa. Mol Microbiol 94:771–793. doi:10.1111/mmi.12802 25243483 PMC4227967

[56] Cai Y, Hutchin A, Craddock J, Walsh MA, Webb JS, Tews I. 2020. Differential impact on motility and biofilm dispersal of closely related phosphodiesterases in Pseudomonas aeruginosa. Sci Rep 10. doi:10.1038/s41598-020-63008-5 PMC7148300 32277108

[57] An S, Wu J, Zhang LH. 2010. Modulation of Pseudomonas aeruginosa biofilm dispersal by a cyclic-Di-GMP phosphodiesterase with a putative hypoxia-sensing domain . Appl Environ Microbiol 76:8160–8173. doi:10.1128/AEM.01233-10 20971871 PMC3008239

[58] Kazmierczak BI, Lebron MB, Murray TS. 2006. Analysis of FimX, a phosphodiesterase that governs twitching motility in Pseudomonas aeruginosa. Mol Microbiol 60:1026–1043. doi:10.1111/j.1365-2958.2006.05156.x 16677312 PMC3609419

[59] Qi Y, Chuah MLC, Dong X, Xie K, Luo Z, Tang K, Liang ZX. 2011. Binding of cyclic diguanylate in the non-catalytic EAL domain of FimX induces a long-range conformational change. J Biol Chem 286:2910–2917. doi:10.1074/jbc.M110.196220 21098028 PMC3024786

[60] Katharios-Lanwermeyer S, Whitfield GB, Howell PL, O’Toole GA. 2021. Pseudomonas aeruginosa Uses c-di-GMP phosphodiesterases RmcA and MorA to regulate biofilm maintenance. MBio 12:mBio doi:10.1128/mBio.03384-20 PMC7858071 33531388

[61] Petrova OE, Cherny KE, Sauer K. 2015. The diguanylate cyclase GcbA facilitates Pseudomonas aeruginosa biofilm dispersion by activating bdlA. J Bacteriol 197:174–187. doi:10.1128/JB.02244-14 25331436 PMC4288676

[62] Kulesekara H, Lee V, Brencic A, Liberati N, Urbach J, Miyata S, Lee DG, Neely AN, Hyodo M, Hayakawa Y, Ausubel FM, Lory S. 2006. Analysis of Pseudomonas aeruginosa diguanylate cyclases and phosphodiesterases reveals a role for bis-(3′-5′)-cyclic-GMP in virulence . Proc Natl Acad Sci USA 103:2839–2844. doi:10.1073/pnas.0511090103 16477007 PMC1413825

[63] Feng Q, Ahator SD, Zhou T, Liu Z, Lin Q, Liu Y, Huang J, Zhou J, Zhang L-H. 2020. Regulation of exopolysaccharide production by ProE, a cyclic-Di-GMP phosphodiesterase in Pseudomonas aeruginosa PAO1. Front Microbiol 11:1226. doi:10.3389/fmicb.2020.01226 32582123 PMC7290235

[64] Cai YM, Yu KW, Liu JH, Cai Z, Zhou ZH, Liu Y, Wang TF, Yang L. 2022. The c-di-GMP phosphodiesterase PipA (PA0285) regulates autoaggregation and Pf4 bacteriophage production in Pseudomonas aeruginosa PAO1. Appl Environ Microbiol 88:e0003922. doi:10.1128/aem.00039-22 35638845 PMC9238385

[65] Mah T-F, Pitts B, Pellock B, Walker GC, Stewart PS, O’Toole GA. 2003. A genetic basis for Pseudomonas aeruginosa biofilm antibiotic resistance. Nature New Biol 426:306–310. doi:10.1038/nature02122 14628055

[66] Poudyal B, Sauer K. 2018. The ABC of biofilm drug tolerance: the MerR-like regulator brlr is an activator of ABC transport systems, with PA1874-77 contributing to the tolerance of Pseudomonas aeruginosa biofilms to tobramycin. Antimicrob Agents Chemother 62:e01981-17. doi:10.1128/AAC.01981-17 29180529 PMC5786766

[67] Zhang L, Mah TF. 2008. Involvement of a novel efflux system in biofilm-specific resistance to antibiotics. J Bacteriol 190:4447–4452. doi:10.1128/JB.01655-07 18469108 PMC2446775

[68] Poole K, Hay T, Gilmour C, Fruci M. 2019. The aminoglycoside resistance-promoting AmgRS envelope stress-responsive two-component system in Pseudomonas aeruginosa is zinc-activated and protects cells from zinc-promoted membrane damage. Microbiology (Reading, Engl) 165:563–571. doi:10.1099/mic.0.000787 30835196

[69] McPhee JB, Bains M, Winsor G, Lewenza S, Kwasnicka A, Brazas MD, Brinkman FSL, Hancock REW. 2006. Contribution of the PhoP-PhoQ and PmrA-PmrB two-component regulatory systems to Mg2+-induced gene regulation in Pseudomonas aeruginosa. J Bacteriol 188:3995–4006. doi:10.1128/JB.00053-06 16707691 PMC1482896

[70] Lee H, Hsu FF, Turk J, Groisman EA. 2004. The PmrA-regulated pmrC gene mediates phosphoethanolamine modification of lipid A and polymyxin resistance in Salmonella enterica . J Bacteriol 186:4124–4133. doi:10.1128/JB.186.13.4124-4133.2004 15205413 PMC421605

[71] Liao J, Sauer K. 2012. The MerR-like transcriptional regulator BrlR contributes to Pseudomonas aeruginosa biofilm tolerance. J Bacteriol 194:4823–4836. doi:10.1128/JB.00765-12 22730129 PMC3430307

[72] Liao Julie, Schurr MJ, Sauer K. 2013. The MerR-like regulator BrlR confers biofilm tolerance by activating multidrug efflux pumps in Pseudomonas aeruginosa biofilms. J Bacteriol 195:3352–3363. doi:10.1128/JB.00318-13 23687276 PMC3719540

[73] Chambers JR, Sauer K. 2013. The MerR-like regulator BrlR impairs Pseudomonas aeruginosa biofilm tolerance to colistin by repressing PhoPQ. J Bacteriol 195:4678–4688. doi:10.1128/JB.00834-13 23935054 PMC3807428

[74] Van Acker H, Coenye T. 2017. The role of reactive oxygen species in antibiotic-mediated killing of bacteria. Trends Microbiol 25:456–466. doi:10.1016/j.tim.2016.12.008 28089288

[75] Fernández-Billón M, Llambías-Cabot AE, Jordana-Lluch E, Oliver A, Macià MD. 2023. Mechanisms of antibiotic resistance in Pseudomonas aeruginosa biofilms. Biofilm 5:100129. doi:10.1016/j.bioflm.2023.100129 37205903 PMC10189392

[76] Kolpen M, Kragh KN, Enciso JB, Faurholt-Jepsen D, Lindegaard B, Egelund GB, Jensen AV, Ravn P, Mathiesen IHM, Gheorge AG, Hertz FB, Qvist T, Whiteley M, Jensen PØ, Bjarnsholt T. 2022. Bacterial biofilms predominate in both acute and chronic human lung infections. Thorax 77:1015–1022. doi:10.1136/thoraxjnl-2021-217576 35017313 PMC9510407

[77] Hall S, McDermott C, Anoopkumar-Dukie S, McFarland AJ, Forbes A, Perkins AV, Davey AK, Chess-Williams R, Kiefel MJ, Arora D, Grant GD. 2016. Cellular effects of pyocyanin, a secreted virulence factor of Pseudomonas aeruginosa. Toxins (Basel) 8:236. doi:10.3390/toxins8080236 27517959 PMC4999852

[78] Pugsley AP. 1993. The complete general secretory pathway in gram-negative bacteria. Microbiol Rev 57:50–108. doi:10.1128/mr.57.1.50-108.1993 8096622 PMC372901

[79] Voulhoux R, Ball G, Ize B, Vasil ML, Lazdunski A, Wu LF, Filloux A. 2001. Involvement of the twin-arginine translocation system in protein secretion via the type II pathway. EMBO J 20:6735–6741. doi:10.1093/emboj/20.23.6735 11726509 PMC125745

[80] Filloux A, Michel G, Bally M. 1998. GSP-dependent protein secretion in gram-negative bacteria: the Xcp system of Pseudomonas aeruginosa. FEMS Microbiol Rev 22:177–198. doi:10.1111/j.1574-6976.1998.tb00366.x 9818381

[81] Ball G, Durand E, Lazdunski A, Filloux A. 2002. A novel type II secretion system in Pseudomonas aeruginosa. Mol Microbiol 43:475–485. doi:10.1046/j.1365-2958.2002.02759.x 11985723

[82] Horna G, Ruiz J. 2021. Type 3 secretion system of Pseudomonas aeruginosa. Microbiol Res 246:126719. doi:10.1016/j.micres.2021.126719 33582609

[83] Yin R, Cheng J, Lin J. 2024. The role of the type VI secretion system in the stress resistance of plant-associated bacteria. Stress Biol 4:16. doi:10.1007/s44154-024-00151-3 38376647 PMC10879055

[84] Garai P, Berry L, Moussouni M, Bleves S, Blanc-Potard AB. 2019. Killing from the inside: Intracellular role of T3SS in the fate of Pseudomonas aeruginosa within macrophages revealed by mgtC and oprF mutants. PLoS Pathog 15:e1007812. doi:10.1371/journal.ppat.1007812 31220187 PMC6586356

[85] Tan X, Cheng X, Xiao J, Liu Q, Du D, Li M, Sun Y, Zhou J, Zhu G. 2023. Alkaline phosphatase LapA regulates quorum sensing-mediated virulence and biofilm formation in Pseudomonas aeruginosa PAO1 under phosphate depletion stress. Microbiol Spectr 11:e0206023. doi:10.1128/spectrum.02060-23 37796007 PMC10715133

[86] Saldías MS, Ortega X, Valvano MA. 2009. Burkholderia cenocepacia O antigen lipopolysaccharide prevents phagocytosis by macrophages and adhesion to epithelial cells. J Med Microbiol 58:1542–1548. doi:10.1099/jmm.0.013235-0 19713359

[87] Tjendana Tjhin V, Oda M, Yamashita M, Iwaki T, Fujita Y, Wakame K, Inagawa H, Soma GI. 2024. Baseline data collections of lipopolysaccharide content in 414 herbal extracts and its role in innate immune activation. Sci Rep 14:15394. doi:10.1038/s41598-024-66081-2 38965275 PMC11224407

[88] Feng X, Deng T, Zhang Y, Su S, Wei C, Han D. 2011. Lipopolysaccharide inhibits macrophage phagocytosis of apoptotic neutrophils by regulating the production of tumour necrosis factor α and growth arrest-specific gene 6. Immunology 132:287–295. doi:10.1111/j.1365-2567.2010.03364.x 21039473 PMC3050451

[89] Matsuura M. 2013. Structural modifications of bacterial lipopolysaccharide that facilitate gram-negative bacteria evasion of host innate immunity. Front Immunol. doi:10.3389/fimmu.2013.00109 PMC3662973 23745121

[90] Steimle A, Autenrieth IB, Frick JS. 2016. Structure and function: lipid A modifications in commensals and pathogens. Int J Med Microbiol 306:290–301. doi:10.1016/j.ijmm.2016.03.001 27009633

[91] Bastaert F, Kheir S, Saint-Criq V, Villeret B, Dang PMC, El-Benna J, Sirard JC, Voulhoux R, Sallenave JM. 2018. Pseudomonas aeruginosa LasB subverts alveolar macrophage activity by interfering with bacterial killing through downregulation of innate immune defense, reactive oxygen species generation, and complement activation. Front Immunol 9:1675. doi:10.3389/fimmu.2018.01675 30083156 PMC6064941

[92] Rumbaugh KP, Sauer K. 2020. Biofilm dispersion. Nat Rev Microbiol 18:571–586. doi:10.1038/s41579-020-0385-0 32533131 PMC8564779

[93] Barraud N, Hassett DJ, Hwang SH, Rice SA, Kjelleberg S, Webb JS. 2006. Involvement of nitric oxide in biofilm dispersal of Pseudomonas aeruginosa. J Bacteriol 188:7344–7353. doi:10.1128/JB.00779-06 17050922 PMC1636254

[94] Liao Y, Smyth GK, Shi W. 2014. FeatureCounts: an efficient general purpose program for assigning sequence reads to genomic features. Bioinformatics 30:923–930. doi:10.1093/bioinformatics/btt656 24227677

[95] Love MI, Huber W, Anders S. 2017. Analyzing RNA-seq data with DESeq2. Bioconductor

[96] Wu T, Hu E, Xu S, Chen M, Guo P, Dai Z, Feng T, Zhou L, Tang W, Zhan L, Fu X, Liu S, Bo X, Yu G. 2021. clusterProfiler 4.0: A universal enrichment tool for interpreting omics data. The Innov 2:100141. doi:10.1016/j.xinn.2021.100141 PMC8454663 34557778

[97] Hölken JM, Teusch N. 2023. The monocytic cell line THP-1 as a validated and robust surrogate model for human dendritic cells. Int J Mol Sci 24:1452. doi:10.3390/ijms24021452 36674966 PMC9866978

[98] Carryn S, Van Bambeke F, Mingeot-Leclercq M-P, Tulkens PM. 2002. Comparative intracellular (THP-1 macrophage) and extracellular activities of beta-lactams, azithromycin, gentamicin, and fluoroquinolones against Listeria monocytogenes at clinically relevant concentrations. Antimicrob Agents Chemother 46:2095–2103. doi:10.1128/AAC.46.7.2095-2103.2002 12069960 PMC127291

[99] Heydorn A, Nielsen AT, Hentzer M, Sternberg C, Givskov M, Ersbøll BK, Molin S. 2000. Quantification of biofilm structures by the novel computer program COMSTAT. Microbiology (Reading) 146 (Pt 10):2395–2407. doi:10.1099/00221287-146-10-2395 11021916

[100] Cueva AR, Pham O, Diaby A, Fleming D, Rumbaugh KP, Fernandes GE. 2020. Pyoverdine assay for rapid and early detection of Pseudomonas aeruginosa in burn wounds. ACS Appl Bio Mater 3:5350–5356. doi:10.1021/acsabm.0c00665 35021709

[101] Kaleta MF, Sauer K. 2023. MoaB1 homologs contribute to biofilm formation and motility by Pseudomonas aeruginosa and Escherichia coli. J Bacteriol 205:e0000423. doi:10.1128/jb.00004-23 37098964 PMC10210980

[102] Hassett DJ, Charniga L, Bean K, Ohman DE, Cohen MS. 1992. Response of Pseudomonas aeruginosa to pyocyanin: mechanisms of resistance, antioxidant defenses, and demonstration of a manganese-cofactored superoxide dismutase. Infect Immun 60:328–336. doi:10.1128/iai.60.2.328-336.1992 1730464 PMC257632

