# Supplementary figures and images for: Distinct transcriptome and traits of freshly dispersed Pseudomonas aeruginosa cells
Source: mSphere. 2024 Nov 27;9(12):e00884-24. doi: 10.1128/msphere.00884-24 (PMC11656770; doi:10.1128/msphere.00884-24)

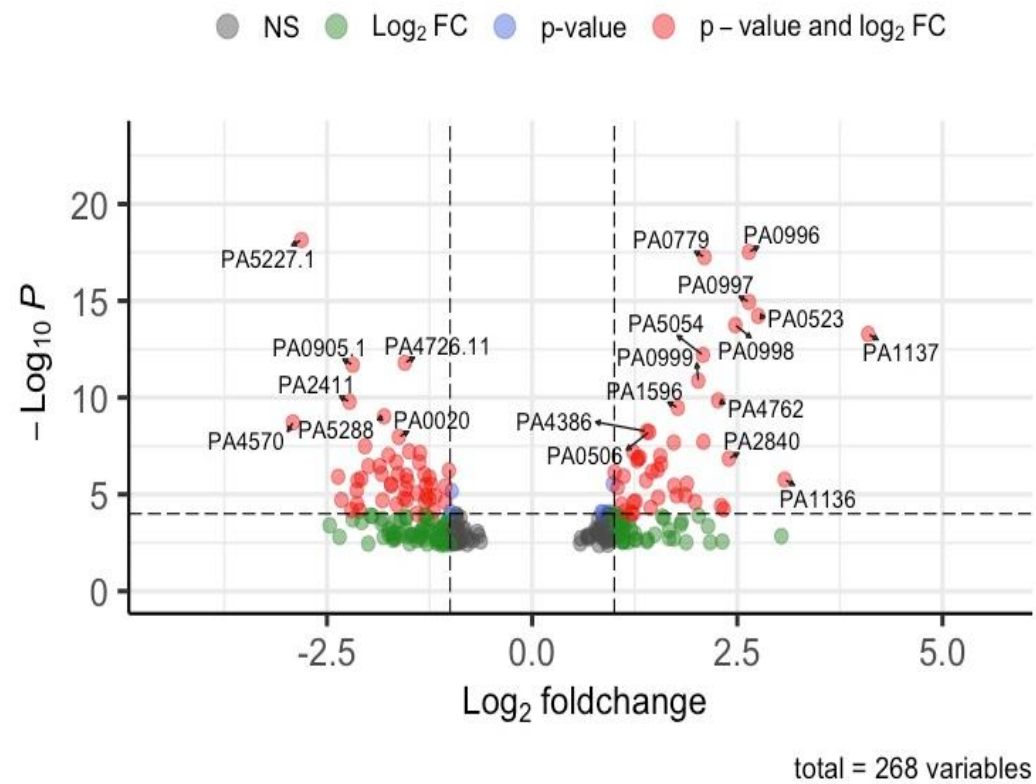

Supplemental Figure 1

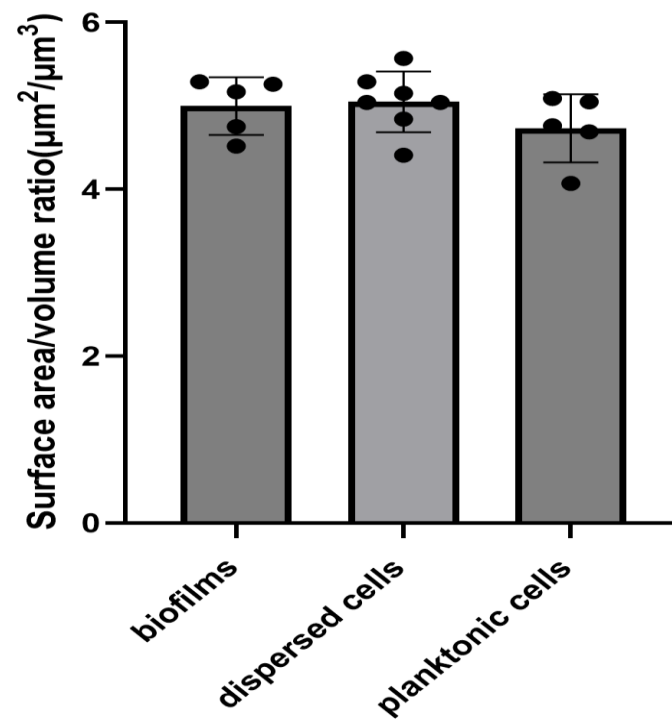

Supplemental Figure 2

Supplement: Supplemental Figures — Figures S1 and S2. [file msphere.00884-24-s0001.pdf]
