# Supplementary material for: Distinct transcriptome and traits of freshly dispersed Pseudomonas aeruginosa cells
Source: mSphere. 2024 Nov 27;9(12):e00884-24. doi: 10.1128/msphere.00884-24 (PMC11656770; doi:10.1128/msphere.00884-24)
Supplement: Legends — Supplemental table and figure legends. [file msphere.00884-24-s0002.pdf]

SUPPLEMENTAL MATERIAL

SUPPLEMENTAL FIGURE LEGENDS

Supplemental Figure 1. Volcano plot of transcriptomic changes in biofilms versus freshly dispersed cells obtained in response to glutamate. Volcano plots were generated by the Enhanced volcano package using a DESeq2 data set, with default log fold-change thresholds of −1 and 1 and an adjusted *P*-value threshold of 0.05. The cutoff value for log2FC was >|2|, and the cutoff value for P value was 10e-5. Only the variables passing the log2FC and P value thresholds are colored red.

Supplemental Figure 2. Quantitative analysis of the surface area to volume ratio of macrophages 90 min post exposure to biofilm cells, planktonic cells, and dispersed cells obtained in response to nitric oxide. Analysis was based on confocal images using COMSTAT. Statistical significance was determined by Anova, and no significant difference was noted.

SUPPLEMENTAL TABLES

Supplemental Table 1. Dispersion transcriptome, showing genes that differentially expressed at least 2-fold between glutamate and nitric oxide dispersed cells, as compared to biofilm and planktonic cells, and planktonic cells exposed to the dispersion signals glutamate and nitric oxide for 30 minutes. Fold change (log2) is relative to biofilm cells. Genes were identified using a negative binomial test with a significance threshold of p-value 0.05.

Supplemental Table 2. Transcript abundance of genes linked to motility and exopolysaccharide biosynthesis. Fold change (log2) is relative to biofilm cells.

Supplemental Table 3. Transcript abundance of genes linked to virulence. Fold change (log2) is relative to biofilm cells.

Supplemental Table 4. Differential expression of genes encoding c-di-GMP modulating enzymes in dispersed and planktonic cells relative to biofilm cells. Fold change (log2) is relative to biofilm cells.

Supplemental Table 5. Primers used in this study.
