# Supplementary material for: Distinct transcriptome and traits of freshly dispersed Pseudomonas aeruginosa cells
Source: mSphere. 2024 Nov 27;9(12):e00884-24. doi: 10.1128/msphere.00884-24 (PMC11656770; doi:10.1128/msphere.00884-24)
Supplement: Table S1 — Dispersion transcriptome. [file msphere.00884-24-s0003.pdf]

Supplemental Table 1. Dispersion transcriptome, showing genes that differentially expressed at least 2-fold between glutamate and nitric oxide dispersed cells, as compared to biofilm and planktonic cells, and planktonic cells exposed to the dispersion signals glutamate and nitric oxide for 30 minutes. Fold change (log2) is relative to biofilm cells. Genes were identified using a negative binomial test with a significance threshold of p-value 0.05.

		Fold change (log2)						
Locus ID	Description	Glutamate dispersed cells		NO dispersed cells		Planktonic cells	Planktonic cells exposed to nitric oxide	Planktonic cells exposed to glutamate
PA0506	acyl-coA dehydrogenase	upregulated	1.574357	upregulated	1.223554	-0.03607159	0.854460506	1.309806264
PA0840	oxidoreductase	upregulated	1.83432	upregulated	2.018073	0.381782741	0.682084945	-0.283798102
PA1093	hypothetical protein	upregulated	1.293367	upregulated	1.228576	-0.896922544	0.728567508	1.045271096
PA1094	fliD-B type flagellar hook-associated protein	upregulated	0.966737	upregulated	0.838656	-0.008645728	0.705878998	0.913001461
PA1136	transcriptional regulator	upregulated	2.874245	upregulated	3.124099	-0.077678751	-0.76050676	-0.574387391
PA1137	oxidoreductase	upregulated	3.332602	upregulated	3.866577	0.097277739	-0.836991019	-0.442285035
PA1581	sdhC-succinate dehydrogenase subunit C	upregulated	1.123353	upregulated	0.880398	-1.102580977	-0.14568256	-0.778990619
PA1787	acnB-aconitate hydratase B	upregulated	1.081276	upregulated	0.702492	-0.487451384	0.88030876	0.708396304
PA2197	conserved hypothetical protein	upregulated	0.710304	upregulated	1.153924	0.270513081	0.903562833	1.265253889
PA2550	acyl-coA dehydrogenase	upregulated	1.42034	upregulated	1.157598	0.170856141	-0.114098508	0.219578144
PA2561	methyl-accepting chemotaxis protein-ctpH	upregulated	1.031822	upregulated	1.155627	0.298175906	0.183737683	-0.144715828
PA2736.1	tRNA-pro	upregulated	0.349694	upregulated	0.895849	3.050204416	3.545674813	3.618813182
PA2840	ATP-dependent RNA helicase	upregulated	2.776142	upregulated	2.419303	-0.055610094	0.586270954	0.054533907
PA3160	O-Antigen chain length regulator	upregulated	0.409712	upregulated	0.700575	-0.305416169	0.037116015	-0.142281734
PA3183	zwf-glucose-6-phosphate 1-dehydrogenase	upregulated	0.992333	upregulated	1.245576	0.869210365	0.371638326	0.120352641
PA3194		upregulated	1.003429	upregulated	1.268914	0.94433558	1.047475955	0.911667468
PA3234	acetate permease	upregulated	0.850115	upregulated	0.722684	-0.530915592	-1.481671968	-1.708628308
PA3235		upregulated	1.168466	upregulated	1.139177	0.063100913	-0.285389091	-1.113075003
PA3742	rplS-50S ribosomal protein L19	upregulated	1.238263	upregulated	1.159481	-0.459022777	-0.363276042	-0.030409147
PA3920	metal transporting P-type ATPase	upregulated	1.274297	upregulated	1.608189	1.281691385	1.225921688	0.749076345
PA4239		upregulated	1.027846	upregulated	0.613988	-1.514675875	-0.750607366	0.077706936
PA4244		upregulated	1.057922	upregulated	0.741539	-1.304157322	-0.946987078	-0.057109116
PA4245	rpmD-50S ribosomal protein L30	upregulated	1.083746	upregulated	0.966522	-1.188821966	-0.751223035	-0.100435353
PA4248		upregulated	1.036283	upregulated	0.702379	-1.321627254	-1.163789374	-0.052187196
PA4249	rpsH-30S ribosomal protein S8	upregulated	1.439878	upregulated	1.194785	-0.586363454	-0.672493312	-0.146470625
PA4255	rpmC-50S ribosomal protein L29	upregulated	0.987271	upregulated	0.700314	-1.137867572	-0.305305419	0.208699573
PA4256	rplP-50S ribosomal protein L16	upregulated	1.448582	upregulated	1.059879	-0.804389824	0.210488482	0.770040679
PA4261	rplW-50S ribosomal protein L23	upregulated	1.18652	upregulated	0.891766	-1.340936433	-1.237304566	-0.486122202
PA4266	fusA1-elongation factor G	upregulated	1.157333	upregulated	0.617394	-0.71907806	-0.158832439	0.577268369
PA4267	rpsG-30S ribosomal protein S7	upregulated	1.082922	upregulated	0.61328	-0.830194301	-0.123953294	0.377667988
PA4273	rplA-50S ribosomal protein L1	upregulated	1.236553	upregulated	0.816959	-0.939020404	-0.329150355	0.41669847
PA4274	rplK-50S ribosomal protein L11	upregulated	1.255703	upregulated	0.907037	-1.1172377	-0.194651488	0.717819863
PA4386		upregulated	0.483919	upregulated	1.052386	0.051776364	1.222363974	1.817329085
PA4430	cytochromeB	upregulated	1.244303	upregulated	0.843705	-0.710752175	1.023120681	0.702744465
PA4658		upregulated	0.939783	upregulated	1.015749	-0.545279377	-0.414668726	0.034485133
PA5316		upregulated	1.352634	upregulated	1.400194	-1.173065684	0.28629971	0.749780423
PA5367	pstA-phosphate ABC transporter permease	upregulated	2.119658	upregulated	1.54155	0.22230709	0.554327809	0.703921471
PA5368	pstC-phosphate ABC transporter permease	upregulated	1.776169	upregulated	1.306284	0.645656389	0.45048942	-0.547815217
PA5369	pstS-(PA5369) phosphate ABC transporter substrate-binding protein	upregulated	2.380728	upregulated	1.58242	-0.138692795	0.070556682	0.60354192
PA0008	glyS-glysyl-tRNA synthetase	upregulated	1.025369	upregulated	0.840142	0.175263227	0.537916966	0.756951144
PA0140	alkylhydroperoxide reductase subunit F	upregulated	1.296643	upregulated	0.635864	0.705662773	1.30897219	1.873603246
PA0283	sulfate binding protein precursor	upregulated	0.569928	upregulated	2.084621	0.840691407	-0.056998768	-0.192807242
PA0432	sahH- S-adenosyl-L-homocysteine hydrolase	upregulated	0.555658	upregulated	0.100262	-0.033177635	0.726200176	0.827259389
PA0508	probable acyl-coA deydrogenase	upregulated	1.638803	upregulated	1.018265	0.4143359	0.051474548	-0.037028226
PA0546	methionine adenosyltransferase	upregulated	0.776164	upregulated	0.340434	-0.458276596	0.189418944	0.482654751
PA0548	transketolase	upregulated	0.64417	upregulated	0.129716	-0.370930757	0.295812047	0.739840942
PA0729.1	tRNA-Gly	upregulated	0.784979	upregulated	1.081866	0.366187579	0.685140441	0.957416564
PA0833	hypothetical protein	upregulated	0.927836	upregulated	1.258061	-0.044015549	1.01791313	0.892228853
PA0888	aotJ-arginine/ornithine binding protein AotJ	upregulated	0.842525	upregulated	0.372018	-0.514684549	0.467985126	0.707074692
PA0889	aotQ- arginine/ornithine transport protein AotQ	upregulated	1.193255	upregulated	0.246144	-0.189763319	-0.16847177	0.358684533
PA0898	aruD-Sucinylglutamate 5-semialdehyde dehydrogenase	upregulated	1.097161	upregulated	0.371946	-0.61718084	-0.549971257	-0.7446734
PA0916	conserved hypothetical protein	upregulated	1.291185	upregulated	0.643439	0.383078088	0.179092393	-0.487230691
PA1076	hypothetical protein		-0.10099	upregulated	0.1182	-0.690124007	-0.582204214	-0.681486827
PA1099	two component response regulator	upregulated	0.84346	upregulated	1.007035	0.456571534	0.880001051	1.329154995
PA1288	oxylipin transporter	upregulated	0.999622	upregulated	0.703785	0.038265862	0.558862663	0.499003012
PA1317	cyoA-cytochrome o ubiquinol oxidase subunit II	upregulated	1.24985	upregulated	0.820095	0.083628814	0.09120562	-0.164897057
PA1318	cyoB-cytochrome o ubiquinol oxidase subunit 1	upregulated	0.840862	upregulated	0.713546	0.465321442	0.246346619	0.68565272
PA1340	AatM	upregulated	1.068743	upregulated	0.607256	-0.238970876	0.281932886	0.540403258
PA1551	probable ferredoxin	upregulated	1.101473	upregulated	1.379079	0.300394369	1.560790588	1.332535856
PA1552	Cytochrome c oxidase, cbb3-type CcoP subunit	upregulated	1.176545	upregulated	0.800953	-0.186485216	0.494212443	0.728160522
PA1553	Cytochrome c oxidase, cbb3-type, coO subunit	upregulated	0.973052	upregulated	0.658904	-0.321433295	0.425881655	0.302351493
PA1554	Cytochrome c oxidase, cbb3-type, coN subunit	upregulated	1.344648	upregulated	0.834431	-0.267087178	0.341173237	0.536570607
PA1585	2-oxoglutrate dehydrogenase (E1 subunit)	upregulated	0.590433	upregulated	0.257621	-0.635876487	0.095881174	0.394982924
PA1588	succinyl-coA synthetase beta chain	upregulated	0.803002	upregulated	0.393744	-0.834845622	0.494392281	0.772024421
PA1673	mhr-microoxic hemerythrin, Mhr		-0.71481	upregulated	0.284462	-0.466391106	0.274375294	0.072333147
PA1796.4	tRNA-His	upregulated	0.659719	upregulated	0.691559	0.951697229	0.692797766	1.94930403
PA1984	exaC	upregulated	0.803372	upregulated	0.446629	0.103821619	-0.270603823	-0.406412281
PA2147	katE- catalase HPII	upregulated	0.960918	upregulated	0.105907	0.326308201	-0.665902435	-0.386673551

PA2252	probable AGCS sodium/alanine/glycine symporter	upregulated	1.003606	upregulated	0.372598	-0.130034861	-1.087102611	-0.637948847
PA2567	hypothetical protein	upregulated	1.437009	upregulated	1.697091	0.836207228	1.190642631	1.24747919
PA2624	isocitrate dehydrogenase	upregulated	0.834501	upregulated	0.710761	-0.224299569	0.11355377	-0.032308388
PA2638	nuoB- NADH Dehydrogenase	upregulated	1.339827	upregulated	1.053319	0.232345004	1.028276044	0.950018566
PA2639	nuoD-NADH dehydrogenase I chain C,D	upregulated	0.908409	upregulated	0.38737	0.245231391	1.334739625	0.872524965
PA2642	nuoG -NADH dehydrogenase I chain G	upregulated	0.932978	upregulated	0.532292	0.415544398	1.540091797	1.564391427
PA2647	nuoL- NADH dehydrogenase I chain L	upregulated	0.971877	upregulated	0.588869	0.616782456	1.523190196	1.484406146
PA2649	nuoN- NADH dehydrogenase I chain N	upregulated	0.842895	upregulated	0.823345	0.777682948	1.158014143	1.141504566
PA2740	pheS-phenylalanine-tRNA synthetase, alpha-subunit	upregulated	0.866551	upregulated	0.452265	-0.528874644	-0.087928494	0.295637098
PA2754	conserved hypothetical protein		-0.60057	upregulated	0.480409	-1.411491679	-0.130110158	0.061655941
PA2830	heat shock protein HtpX	upregulated	0.487969	upregulated	1.124719	0.144476313	1.364650626	1.66665087
PA2976	ribonucleaseE	upregulated	0.68855	upregulated	0.378461	-0.361059669	-0.053309077	0.502437474
PA3044	two component sensor RocS2		-0.81144	upregulated	0.056449	1.47212258	-0.039448073	-0.386760572
PA3068	gdhB- NAD-dependent glutamate dehydrogenase	upregulated	0.628916	upregulated	0.11409	-0.519139321	-0.019095656	-0.287473241
PA3126	heat-shock protein IbpA	upregulated	0.061266	upregulated	0.868018	0.347354991	0.780663527	1.160496393
PA3262.1	tRNA-Asp	upregulated	0.590536	upregulated	0.338509	-0.393452344	-0.039398461	0.70614821
PA3385	amrZ-alginate and motility regulator Z	upregulated	1.734455	upregulated	1.610377	-0.081466145	0.322223151	0.740349996
PA3418	ldh-leucine dehydrogenase		-0.35858	upregulated	0.014165	-0.606817447	0.236517159	0.100708674
PA3637	CTP synthase	upregulated	0.880197	upregulated	0.62778	-0.2976735	0.651462876	0.859212453
PA3700	lysyl-tRNA synthetase	upregulated	1.021677	upregulated	0.73513	-0.507196309	-0.021837355	0.624762603
PA3768	probable metallo-oxidoreductase		-0.73303	upregulated	0.061489	-0.221552835	-1.691693124	0.381951231
PA3769	GMP synthase	upregulated	0.878636	upregulated	0.30658	-0.274437679	0.427315868	0.928184813
PA3770	inosine -5'-monophosphate dehydrogenase	upregulated	0.855098	upregulated	0.448326	-0.223571796	0.690646028	1.24817611
PA3814	L-cysteine desulfurase (pyridoxal phosphate-dependent)	upregulated	0.76699	upregulated	0.410849	-0.416673644	0.326080447	0.643818958
PA3913	probable protease	upregulated	0.797904	upregulated	1.297057	-0.846193065	0.598122137	-0.59213362
PA3930	cyanide insensitive terminal oxidase	upregulated	1.13987	upregulated	0.258596	0.562204203	1.415056703	0.912465946
PA3972	probable acyl-coA dehydrogenase	upregulated	1.014161	upregulated	1.2143	0.284479673	0.579171235	0.944260909
PA4053	6,7-dimethyl-8-ribityllumazine synthase	upregulated	0.797768	upregulated	0.456159	-0.854190065	-0.64142964	0.119668202
PA4131	probable iron sulfur protein	upregulated	1.332596	upregulated	0.423339	-0.253979086	1.101328787	0.600523513
PA4133	cytochrome c oxidse subunit(cbb3 type)	upregulated	0.661268	upregulated	0.344249	-0.229400289	0.545413259	1.148056824
PA4237	50S ribosomal protein L17	upregulated	0.924128	upregulated	0.695099	-1.510419666	-0.349227573	0.41128518
PA4252	50S ribosomal protein L24	upregulated	0.815203	upregulated	0.508448	-1.09097954	-0.67866186	-0.071802573
PA4259	30S ribosomal protein S19	upregulated	0.840249	upregulated	0.998411	-0.730168113	-0.492441107	0.073091889
PA4265	elongation factor Tu	upregulated	0.678767	upregulated	0.365132	-1.07032705	-0.396133576	0.183121157
PA4269	DNA directed RNA Polymerase beta chain	upregulated	0.680811	upregulated	0.298718	-0.849169905	0.06214006	0.688639895
PA4270	DNA directed RNA Polymerase beta chain	upregulated	0.546756	upregulated	0.231753	-0.708128552	-0.252202095	0.364213471
PA4277	elongation factor Tu	upregulated	0.831075	upregulated	0.498717	-1.121889209	-0.440406017	-0.005095742
PA4333	probable fumrase	upregulated	0.849468	upregulated	0.634796	0.130391013	0.262090942	0.105818354
PA4352	conserved hypothetical protein		-0.9037	upregulated	0.078307	-0.24667895	0.698812369	0.987235101
PA4356	xenobiotic reductase		-0.51786	upregulated	0.100857	0.906324859	-0.699380011	1.244538105
PA4385	GroEL protein	upregulated	0.555218		0.900314	-0.010271094	1.206915421	1.677991484
PA4429	probable cytochrome c1 precursor	upregulated	0.950586	upregulated	0.480124	-0.662516747	0.621255319	0.212070511
PA4431	probable iron-sulfur protein	upregulated	0.893213	upregulated	0.335328	-0.562388963	0.137584254	-0.006261555
PA4519	ornithine decarboxylase	upregulated	0.781826	upregulated	0.334712	-0.682152776	-0.434004978	-0.485981894
PA4598	resistance-nodulation-cell division(RND) multidrug efflux transporter MexD	upregulated	1.737234	upregulated	0.604697	1.333838088	0.047018633	0.289721544
PA4602	serine hydroxymethyltransferase	upregulated	1.056377	upregulated	0.755105	-0.725890487	-0.044229925	0.313001504
PA4758	carbamoyl-phosphate synthase small chain	upregulated	0.761742	upregulated	0.975523	-1.08603815	-0.18201701	0.319604335
PA4759	dihydrodipicolinate reductase	upregulated	0.910949	upregulated	1.203546	-0.008401994	1.181870623	1.830333328
PA4761	DnaK protein	upregulated	0.152286	upregulated	0.941725	-0.335071442	0.996179312	1.516667222
PA4855	phosphoribosylamine-glycine ligase	upregulated	1.202422	upregulated	0.740721	-0.074171916	-0.860049382	0.226534159
PA4929	NicD	upregulated	1.348215	upregulated	1.124534	1.039967913	1.19703088	1.079144296
PA5015	pyruvate dehydrogenase	upregulated	0.599174	upregulated	0.454132	-0.15110843	0.66996471	0.804900127
PA5053	heat shock protein HslV	upregulated	1.34497	upregulated	1.759096	0.152390025	0.738915675	1.410461997
PA5098	hutH-histidine ammonia-lyase	upregulated	1.021263	upregulated	0.482614	0.261958962	-0.324178534	-0.459987014
PA5119	glnA-glutamine synthetase	upregulated	0.474793	upregulated	0.541001	-0.536342231	0.189974113	0.381330311
PA5315	50S ribosomal protein L33	upregulated	0.84785	upregulated	0.761561	-0.767739345	-0.614325518	-0.136025338
PA5366	ATP binding component of ABC phosphate transporter	upregulated	1.401961	upregulated	0.735852	-0.174470348	-0.020949862	0.217637061
PA5427	alcohol dehydrogenase		-0.79947	upregulated	0.06287	-1.537787438	-0.299142686	0.228962536
PA5490	cc4- cytochrome c4 precursor	upregulated	0.897352	upregulated	0.531742	-0.666071606	0.377835146	0.321879949
PA5495	homoserine kinase		-0.04312	upregulated	0.320808	-0.635109015	0.280591514	0.753015216
PA0027	hypothetical protein	downregulated	-1.14918	downregulated	-1.38885	-0.313403117	-0.204203733	-0.127282933
PA0039	hypothetical protein	downregulated	-0.97711	downregulated	-1.29894	-0.901670102	-1.576852077	-1.759096136
PA0078	tssL1	downregulated	-1.61908	downregulated	-1.653	-1.781829215	-2.218036727	-1.938807891
PA0396	pilU twitching motility protein	downregulated	-0.81476	downregulated	-1.03689	-1.14910541	-0.741521489	-0.692905437
PA0451	conserved hypothetical protein	downregulated	-1.49988	downregulated	-0.92378	-1.031021514	-1.659260012	-1.058103111
PA0553	hypothetical protein	downregulated	-0.78642	downregulated	-0.65239	0.068702228	-0.942769733	-0.16104086
PA0657	probable ATPase	downregulated	-1.54727	downregulated	-1.28335	-1.124343964	-0.659571244	-0.635901547
PA0665	iron-sulfur cluster insertion prtein ErpA	downregulated	-0.54209	downregulated	-0.56788	-0.469901592	-0.676695261	-0.363745701
PA0668.5	5S ribosomalRNA	downregulated		downregulated				
PA0672	heme oxygenase	downregulated	-0.49761	downregulated	-0.14531	0.246195672	-2.949934011	0.899146377
PA0905.1	tRNA-Ser	downregulated	-1.15073	downregulated	-1.1498	-0.876266333	-2.713548875	-1.39298048
PA1013.1	tRNA-ser	downregulated	-0.45042	downregulated	-0.31687	-0.723585365	-1.408433016	-0.649085601
PA1134	hypothetical protein	downregulated	-1.22292	downregulated	-0.28335	0.048097224	-0.885765685	-0.606536875
PA1159	cold shock protein	downregulated	-1.30326	downregulated	-1.63393	-0.838648457	-2.617801315	-2.722582915
PA1248	aprF-alkaline protease secretion protein AprF	downregulated	-1.33189	downregulated	-1.04807	0.174192743	-0.868823393	-1.00463188

PA1300	ECF subfamily sigma-70 factor	downregulated	-1.44306	downregulated	-0.88122	-0.997982549	-3.573322458	-0.901777947
PA1301	transmembrane sensor	downregulated	-1.7332	downregulated	-1.29064	-0.757276345	-3.011239266	-0.584114058
PA1392	hypothetical protein	downregulated	-1.75365	downregulated	-1.13984	0.312447164	-0.66922107	-0.686385085
PA1530.1	ffs			downregulated				
PA1604	hypothetical protein	downregulated	-1.08326	downregulated	-0.02943	-0.967900856	-0.463243134	-0.721042095
PA1796.3	tRNA-Leu	downregulated	-0.9461	downregulated	-0.38983	0.362015421	-0.642620006	0.295571661
PA2034	hypothetical protein	downregulated	-1.29795	downregulated	-0.98811	-2.216985838	-5.030133937	-1.465506935
PA2389	pvdR-pyoverdine biosynthesis PvdR	downregulated	-1.32394	downregulated	-1.31531	-1.318347241	-3.611059256	-1.696243097
PA2570.1	tRNA-Leu	downregulated	-0.24898	downregulated	-0.61465	-0.790139528	-1.566508354	-1.21689045
PA2591	vqsR	downregulated	-0.86386	downregulated	-0.70927	-0.567060137	-1.26025093	-1.231000204
PA2662	conserved hypothetical protein		1.397852	downregulated	-0.46411	-0.689832598	0.108575859	-0.988758103
PA2738	himA-integration hostfactor subunit alpha	downregulated	-0.96275	downregulated	-0.95322	-1.247695084	-0.908593487	-0.748091459
PA2852.1		downregulated	-1.98698	downregulated	-0.28953	-1.158139439	-1.81582022	-1.139052809
PA3104	xcpP- secretin protein XcpP	downregulated	-0.87546	downregulated	-0.68226	-1.264054124	-1.256473366	-1.137024848
PA3115	fimV-Motility protein FimV	downregulated	-0.58301	downregulated	-0.53673	-0.959505313	-0.529385719	-0.37185517
PA3224	hypothetical protein	downregulated	-0.99838	downregulated	-0.28753	-1.38482571	-1.167826548	-0.30363536
PA3309	conserved hypothetical protein	downregulated	-1.17036	downregulated	-0.51119	-1.641516214	-0.892653969	-0.542160246
PA3417	probable pyruvate dehydrogenase E1 component, alpha subunit	downregulated	-1.36532	downregulated	-0.31572	-0.546934587	-0.124386122	-0.158656602
PA3530	hypothetical protein	downregulated	-1.93133	downregulated	-1.08977	-0.730715489	-3.850725022	-1.45048433
PA3749	probable major facilitator superfamily (MFS) transporter	downregulated	-1.75895	downregulated	-1.958	-0.94586248	-1.998614161	-2.027507482
PA3945	conserved hypothetical protein	downregulated	-0.73707	downregulated	-0.49597	-1.105634337	-0.994377033	-1.208187998
PA4132	MpaR		0.800486	downregulated	-0.02167	0.162665934	1.054414938	0.722686256
PA4168	second ferric pyoverdine receptor FpvB	downregulated	-1.03613	downregulated	-0.6785	-0.637040576	-1.085239487	-0.958013626
PA4227	pchR-transcriptional regulator PchR	downregulated	-2.02201	downregulated	-1.43216	-0.627728754	-3.342107828	-0.018486289
PA4404		downregulated	-1.45882	downregulated	-1.56322	-1.17442831	-1.670659969	-1.242567745
PA4470	fumC1-fumarate hydratase	downregulated	-1.57899	downregulated	-0.9378	-1.299094917	-4.556206378	-0.340546052
PA4471	hypothetical protein	downregulated	-1.40406	downregulated	-0.61311	-1.384389367	-3.56444123	-0.503857288
PA4550		downregulated	-1.27935	downregulated	-1.05859	-1.16195471	-1.33624954	-1.749591923
PA4607		downregulated	-1.16974	downregulated	-1.67189	-0.055414083	-0.50987984	-0.477852705
PA4613	katB, catalase		1.582839	downregulated	-0.20076	0.297715391	0.591408559	-0.029826161
PA4614		downregulated	-0.64012	downregulated	-0.85867	-0.376208023	0.117538468	-0.100147014
PA4690.1	5S ribosomalRNA	downregulated		downregulated				
PA4709	phuS	downregulated	-1.41225	downregulated	-0.71866	0.054056865	-3.368153695	0.849673363
PA5039	aroK- shikimate kinase	downregulated	-1.2024	downregulated	-1.08712	-1.15168657	-1.641550219	-1.224817818
PA5208	conserved hypothetical protein	downregulated	-0.39574	downregulated	-0.30675	-0.394358198	-0.60155314	-0.346902239
PA5227.1	ssrS-ncRNA	downregulated	-1.38714	downregulated	-1.31138	-0.341432156	-2.119226796	-1.78844357
PA5369.1	5S ribosomalRNA	downregulated		downregulated				
PA5475	hypothetical protein	downregulated	-0.87033	downregulated	-0.06788	-1.688761788	-0.930853901	-0.733474889
PA0256	hypothetical protein	downregulated	-0.22622	downregulated	-0.40584	-0.0959537	-0.158196604	-0.513786251
PA0423	PasP	downregulated	-0.75411	downregulated	-0.74138	-1.330831429	-0.710209103	-0.19146076
PA0706	cat-chloramphenicol acetyltransferase	downregulated	-0.70645	downregulated	-1.06702	-1.065344479	-2.008549186	-1.646858224
PA0867	membrane bound lysozyme inhibotor of c-type lysozyme MilC	downregulated	-0.96531	downregulated	-1.32176	-1.145413362	-1.50175045	-1.041949321
PA1245	AprX	downregulated	-0.82295	downregulated	-0.86183	-0.675150505	-1.919725363	-0.952104447
PA1430	transcriptional regulator LasR	downregulated	-0.35513	downregulated	-0.42317	-0.869834802	-0.724185715	-0.767712286
PA1804	DNA binding protein HU	downregulated	-0.48589	downregulated	-0.7316	-1.015601071	-1.484891803	-0.949764684
PA1869	Acp1	downregulated	-0.82325	downregulated	-1.13489	-1.056693542	-1.297954212	-1.46072975
PA3623	conserved hypothetical protein	downregulated	-0.75899	downregulated	-0.6439	-0.651630001	-0.075521005	-0.043788859
PA3808	conserved hypothetical protein	downregulated	-0.44342	downregulated	-0.86057	-0.791967219	-0.412437108	-0.355600555
PA4408	cell division protein FtsA	downregulated	-0.51826	downregulated	-0.96422	-0.355105861	0.080844423	-0.024158027
PA4466	probable phosphoryl carrier protein	downregulated	-0.10875	downregulated	-0.17412	-0.852887699	0.160787714	-0.975020199
PA4876	osmE	downregulated	-0.26483	downregulated	-0.46575	-0.713625378	-0.752211803	-1.036412064
