# Supplementary material for: Distinct transcriptome and traits of freshly dispersed Pseudomonas aeruginosa cells
Source: mSphere. 2024 Nov 27;9(12):e00884-24. doi: 10.1128/msphere.00884-24 (PMC11656770; doi:10.1128/msphere.00884-24)
Supplement: Table S2 — Transcript abundance of genes linked to motility and exopolysaccharide biosynthesis. [file msphere.00884-24-s0004.docx]

**Supplemental Table 2. Transcript abundance of genes linked to motility and exopolysaccharide biosynthesis.** Fold change (log2) is relative to biofilm cells.

|  |  |  |  | **Fold change (log2)** | | |
| --- | --- | --- | --- | --- | --- | --- |
| **Category**  **Category** | **Locus ID** | **Gene** | **Gene Product** | **Glutamate dispersed cells** | **Nitric oxide dispersed cells** | **Planktonic cells** |
| **Flagella** | PA1077 | flgB | flagellar basal-body rod protein FlgB | -0.47247 | -0.10804 | -0.64989 |
|  | PA1078 | flgC | flagellar basal-body rod protein FlgC | 1.033785 | 1.226767 | 0.612085 |
|  | PA1079 | flgD | flagellar basal-body rod modification protein FlgD | -0.10304 | 0.358703 | -0.33968 |
|  | PA1080 | flgE | flagellar hook protein FlgE | 0.353743 | 0.599736 | -0.05457 |
|  | PA1081 | flgF | flagellar basal-body rod protein FlgF | -0.04562 | -0.04216 | -0.50298 |
|  | PA1082 | flgG | flagellar basal-body rod protein FlgG | 0.211178 | 0.518986 | -0.4277 |
|  | PA1083 | flgH | flagellar L-ring protein precursor FlgH | 0.404882 | 0.367167 | -0.92585 |
|  | PA1084 | flgI | flagellar P-ring protein precursor FlgI | -0.08375 | -0.09967 | -0.02602 |
|  | PA1085 | flgJ | flagellar protein FlgJ | 0.294761 | 0.482438 | 0.280722 |
|  | PA1086 | flgK | flagellar hook-associated protein 1 FlgK | 0.06571 | 0.579181 | 0.350755 |
|  | PA1087 | flgL | flagellar hook-associated protein type 3 FlgL | 0.547703 | 0.557931 | 0.396208 |
|  | PA1092 | fliC | flagellin type B | 1.179553 | 1.248113 | -1.13614 |
|  | PA1093 |  | hypothetical protein | 1.750969 | 1.769221 | -0.4796 |
|  | PA1094 | fliD | flagellar capping protein FliD | 0.927776 | 1.101826 | 0.316452 |
|  | PA1095 |  | hypothetical protein | 0.946938 | 0.221607 | -1.36836 |
|  | PA1096 |  | hypothetical protein | 1.295392 | 0.588009 | -1.27616 |
|  | PA1097 | fleQ | transcriptional regulator FleQ | 0.068748 | 0.138079 | 0.861323 |
|  | PA1098 | fleS | two-component sensor | 0.469827 | 0.650688 | 0.869948 |
|  | PA1099 | fleR | two-component response regulator | 1.393269 | 1.098931 | 0.628527 |
|  | PA1100 | fliE | flagellar hook-basal body complex protein FliE | 0.315555 | -0.13713 | -0.78056 |
|  | PA1101 | fliF | Flagella M-ring outer membrane protein precursor | 0.625031 | 0.759102 | 0.029669 |
|  | PA1102 | fliG | flagellar motor switch protein FliG | -0.01741 | 0.378932 | 0.166733 |
|  | PA1103 |  | probable flagellar assembly protein | -0.11618 | -0.04203 | 0.486679 |
|  | PA1104 | fliI | flagellum-specific ATP synthase FliI | -0.1446 | 0.323321 | 0.284467 |
|  | PA1105 | fliJ | flagellar protein FliJ | -0.04976 | 0.168965 | 0.788121 |
|  | PA1441 |  | hypothetical protein | 0.281466 | 0.125288 | -0.19653 |
|  | PA1442 |  | conserved hypothetical protein | -0.13803 | 0.064477 | -0.64703 |
|  | PA1443 | fliM | flagellar motor switch protein FliM | -0.47316 | 0.130662 | -0.31003 |
|  | PA1444 | fliN | flagellar motor switch protein FliN | -0.00812 | 0.088255 | -3.46E-05 |
|  | PA1445 | fliO | flagellar protein FliO | -0.57691 | 0.304733 | 0.223034 |
|  | PA1446 | fliP | flagellar biosynthetic protein FliP | 0.014988 | 0.87361 | 0.510387 |
|  | PA1447 | fliQ | flagellar biosynthetic protein FliQ | -0.23195 | -0.1586 | -0.00252 |
|  | PA1448 | fliR | flagellar biosynthetic protein FliR | -0.24815 | 0.636765 | 0.231206 |
|  | PA1449 | flhB | flagellar biosynthetic protein FlhB | 0.614972 | -0.18463 | 0.355168 |
|  | PA1452 | flhA | flagellar biosynthesis protein FlhA | 0.152383 | -0.14207 | 0.058034 |
|  | PA1453 | flhF | flagellar biosynthesis protein FlhF | 0.387978 | 0.14618 | 0.397457 |
|  | PA1454 | fleN | flagellar synthesis regulator FleN | -0.10535 | 0.284797 | 0.374089 |
|  | PA1455 | fliA | sigma factor FliA | -0.09988 | -0.19622 | 0.137439 |
|  | PA3350 |  | hypothetical protein | -0.10986 | -0.67082 | -0.19164 |
|  | PA3351 | flgM | See pseudomonas.com | 0.780661 | -0.02985 | -0.62765 |
|  | PA3352 |  | hypothetical protein | 0.847042 | 0.788528 | -0.62015 |
|  | PA4953 | motB | chemotaxis protein MotB | 0.599523 | 0.531362 | 0.613809 |
|  | PA4954 | motA | chemotaxis protein MotA | 0.479395 | 0.716265 | -0.19095 |
|  | PA1460 | motC | MotC | 0.188924 | 0.238879 | 0.019161 |
|  | PA1461 | motD | MotD | 0.462426 | 0.237056 | -0.0517 |
| **Type IV pili biosynthesis** | PA4525 | pilA | type 4 fimbrial precursor PilA | -0.57524 | -0.16034 | -3.04667 |
|  | PA4526 | pilB | type 4 fimbrial biogenesis protein PilB | -0.5715 | -0.1659 | -0.87125 |
|  | PA4527 |  | pseudo=true | -0.0858 | -0.20583 | -1.11509 |
|  | PA4528 | pilD | type 4 prepilin peptidase PilD | -0.0858 | -0.20583 | -1.11509 |
|  | PA4556 | pilE | type 4 fimbrial biogenesis protein PilE | -1.03814 | -0.4449 | -1.16332 |
|  | PA3805 | pilF | type 4 fimbrial biogenesis protein PilF | 0.201259 | 0.537759 | -0.30489 |
|  | PA5044 | pilM | type 4 fimbrial biogenesis protein PilM | -0.78748 | -1.55239 | -1.03001 |
|  | PA5043 | pilN | type 4 fimbrial biogenesis protein PilN | -1.10633 | -1.12599 | -1.03077 |
|  | PA5042 | pilO | type 4 fimbrial biogenesis protein PilO | -1.60353 | -1.49478 | -1.11415 |
|  | PA5041 | pilP | type 4 fimbrial biogenesis protein PilP | -1.18107 | -1.37659 | -1.09704 |
|  | PA5040 | pilQ | Type 4 fimbrial biogenesis outer membrane protein PilQ precursor | -0.96075 | -0.98095 | -1.35086 |
|  | PA0395 | pilT | twitching motility protein PilT | -0.91317 | -0.69328 | -1.09188 |
|  | PA0396 | pilU | twitching motility protein PilU | -1.09186 | -0.99845 | -0.75357 |
|  | PA4551 | pilV | type 4 fimbrial biogenesis protein PilV | -0.49445 | -0.87134 | -1.27875 |
|  | PA4552 | pilW | type 4 fimbrial biogenesis protein PilW | -1.17985 | -0.52115 | -1.2459 |
|  | PA4553 | pilX | type 4 fimbrial biogenesis protein PilX | -0.6998 | -0.7525 | -0.48236 |
|  | PA4554 |  | type 4 fimbrial biogenesis protein PilY1 | -0.49755 | -0.45093 | -0.63602 |
|  | PA4555 |  | type 4 fimbrial biogenesis protein PilY2 | -0.23586 | -0.44281 | -1.30185 |
|  | PA2960 | pilZ | type 4 fimbrial biogenesis protein PilZ | -0.64447 | -0.71972 | -0.57551 |
|  | PA4549 | fimT | type 4 fimbrial biogenesis protein FimT | -1.36234 | -1.95949 | -1.15399 |
|  | PA4550 | fimU | type 4 fimbrial biogenesis protein FimU | -1.50941 | -1.62738 | -1.0542 |
|  | PA3115 | fimV | Motility protein FimV | -0.55177 | -0.80635 | -0.6154 |
|  | PA4547 | pilR | two-component response regulator PilR | -0.64825 | 0.142997 | 0.458791 |
|  | PA4546 | pilS | two-component sensor PilS | -1.2377 | -0.88681 | -0.38677 |
| **Type IV pili related proteins** | PA0408 | pilG | twitching motility protein PilG | -0.36268 | -0.99199 | -1.22952 |
|  | PA0409 | pilH | twitching motility protein PilH | -0.46369 | -0.92142 | -1.28864 |
|  | PA0410 | pilI | twitching motility protein PilI | -0.83993 | -0.36462 | -1.19014 |
|  | PA0411 | pilJ | twitching motility protein PilJ | -0.67422 | -0.66723 | -1.57536 |
|  | PA0412 | pilK | methyltransferase PilK | -0.90145 | -1.39912 | -1.11688 |
|  | PA0413 | chpA | See pseudomonas.com | -0.54688 | -0.65338 | -0.63674 |
|  | PA0414 | chpB | probable methylesterase | -0.31902 | -0.68703 | -1.22491 |
|  | PA0415 | chpC | probable chemotaxis protein | 1.568943 | -0.09759 | 0.8865 |
|  | PA0416 | chpD | probable transcriptional regulator | 0.79777 | 0.641416 | 0.948965 |
|  | PA0417 | chpE | probable chemotaxis protein | -0.46059 | -1.01154 | -0.21117 |
| **Alginate biosynthesis** | PA3540 | algD | GDP-mannose 6-dehydrogenase AlgD | -0.01297 | -0.56131 | 0.605051 |
|  | PA3541 | alg8 | alginate biosynthesis protein Alg8 | 0.293935 | -0.27501 | 0.727914 |
|  | PA3542 |  | alginate biosynthesis protein Alg44 | 0.750141 | -0.66477 | 0.383486 |
|  | PA3543 | algK | alginate biosynthetic protein AlgK precursor | 1.43346 | 0.363573 | 1.879911 |
|  | PA3544 | algE | Alginate production outer membrane protein AlgE precursor | -0.68558 | -0.72508 | -0.65978 |
|  | PA3545 | algG | alginate-c5-mannuronan-epimerase AlgG | 0.629093 | -0.03609 | 0.289433 |
|  | PA3546 | algX | alginate biosynthesis protein AlgX | 1.773283 | 1.518519 | 2.324664 |
|  | PA3547 | algL | poly(beta-d-mannuronate) lyase precursor AlgL | 0.114488 | -1.08166 | 1.158179 |
|  | PA3548 | algI | alginate o-acetyltransferase AlgI | 0.676466 | 0.280045 | 1.124636 |
|  | PA3549 | algJ | alginate o-acetyltransferase AlgJ | 0.980853 | 0.230854 | 1.72913 |
|  | PA3550 | algF | alginate o-acetyltransferase AlgF | -0.81345 | 0.072028 | 0.407753 |
|  | PA3551 | algA | alginate o-acetyltransferase AlgF | 1.012025 | 0.901733 | 1.847406 |
|  | PA5322 | algC | phosphomannomutase AlgC | 0.052729 | 0.580979 | -0.90528 |
| **Alginate regulation** | PA0762 | algU | sigma factor AlgU | -0.58507 | -0.95936 | -1.25498 |
|  | PA0763 | mucA | anti-sigma factor MucA | -0.31604 | -0.96438 | -0.95554 |
|  | PA0764 | mucB | negative regulator for alginate biosynthesis MucB | 0.776805 | 0.502111 | 0.016375 |
|  | PA0765 | mucC | positive regulator for alginate biosynthesis MucC | 0.101472 | 0.148108 | -0.06879 |
|  | PA0766 | mucD | serine protease MucD precursor | 0.169242 | 0.451153 | 0.489318 |
|  | PA5261 | algR | alginate biosynthesis regulatory protein AlgR | -0.45666 | -0.37216 | 0.161537 |
|  | PA5262 | algZ | alginate biosynthesis protein AlgZ/FimS | -0.31711 | -0.3476 | -0.03862 |
|  | PA4446 | algW | AlgW protein | 0.255332 | 0.634414 | -0.2814 |
|  | PA4033 |  | hypothetical protein | 3.69643 | 3.97135 | 4.277369 |
|  | PA3649 |  | conserved hypothetical protein | -0.14956 | 0.00842 | -0.69553 |
|  | PA5253 | algP | alginate regulatory protein AlgP | 0.316971 | -0.16632 | 0.827662 |
|  | PA5255 | algQ | Alginate regulatory protein AlgQ | -0.68151 | -0.55199 | 0.011385 |
| **Pel polysaccharide** | PA3064 | pelA | See pseudomonas.com | 1.464759 | 0.449031 | 1.339318 |
|  | PA3063 | pelB | See pseudomonas.com | 0.969111 | 0.17291 | 1.036065 |
|  | PA3062 | pelC | See pseudomonas.com | 0.80084 | -0.05381 | 0.878769 |
|  | PA3061 | pelD | See pseudomonas.com | 0.450701 | -0.32476 | 0.187211 |
|  | PA3060 | pelE | See pseudomonas.com | 0.396689 | 0.321227 | 0.802454 |
|  | PA3059 | pelF | See pseudomonas.com | 0.658756 | 0.24913 | 1.165153 |
|  | PA3058 | pelG | See pseudomonas.com | -0.90537 | -0.4581 | -0.68506 |
| **Psl polysaccharide** | PA2231 | pslA | See pseudomonas.com | 0.772569 | 0.534348 | 0.526156 |
|  | PA2232 | pslB | PslB | 0.452857 | 0.438083 | 0.893674 |
|  | PA2233 | pslC | PslC | 0.652916 | 0.501836 | 1.149674 |
|  | PA2234 | pslD | PslD | 0.569723 | 0.819132 | 0.923372 |
|  | PA2235 | pslE | PslE | 0.125647 | 0.670886 | 0.879117 |
|  | PA2236 | pslF | PslF | -0.01543 | 0.181447 | 0.884699 |
|  | PA2237 | pslG | PslG | 0.228394 | 0.72169 | 0.706545 |
|  | PA2238 | pslH | PslH | 0.091674 | 0.645311 | 0.424585 |
|  | PA2239 | pslI | PslI | -0.5399 | 0.346634 | 0.440027 |
|  | PA2240 | pslJ | PslJ | -0.2914 | 0.325382 | 0.301922 |
|  | PA2241 | pslK | PslL | -0.22209 | 0.015185 | 0.017671 |
|  | PA2242 | pslL | hypothetical protein | 0.400014 | 0.994563 | 0.393946 |
|  | PA2243 | pslM | hypothetical protein | -0.09446 | -0.35009 | 0.433435 |
|  | PA2244 | pslN | hypothetical protein | -0.41132 | -0.30839 | 0.741416 |
|  | PA2245 | pslO | hypothetical protein | 0.123685 | -1.64114 | 0.218858 |
| **Chemotaxis** | PA0173 |  | probable methyltransferase | 1.01 | 0.91 | 1.86 |
|  | PA0174 |  | conserved hypothetical protein | 1.2 | 1.43 | 0.82 |
|  | PA0175 |  | cheR2 | 1.32 | 1.45 | 2.06 |
|  | PA0176 |  | aerotaxis transducer Aer2 | 0.963 | 1.39 | 1.9 |
|  | PA0177 |  | Probable purine-binding chemotaxis protein | 0.9 | 1.7 | 1.82 |
|  | PA0178 |  | probable two component sensor | 0.43 | 1.01 | 1.46 |
|  | PA0179 |  | probable two component response regulator | 0.44 | 1.18 | 1.33 |
|  | PA1102 |  | flagellar motor switch protein FliG | -0.017 | 0.37 | 0.166 |
|  | PA1423 |  | BdlA | 0.699 | -0.02 | -1.54 |
|  | PA1443 |  | Flagellar motor switch protein FliM | -0.473 | 0.13 | -0.31 |
|  | PA1444 |  | Flagellar motor switch protein FliN | -0.008 | 0.088 | -3.4 |
|  | PA1456 |  | two component response rgeulator CheY | -0.22 | -0.138 | 0.38 |
|  | PA1457 |  | chemotaxis protein CheZ | -0.34 | -0.07 | -0.11 |
|  | PA1458 |  | probable two component sensor | 0.38 | 0.236 | 0.088 |
|  | PA1459 |  | probable methyltransferase | 0.142 | 0.004 | -0.01 |
|  | PA1460 |  | motC | 0.486 | 0.327 | 0.019 |
|  | PA1461 |  | motD | -0.232 | 0.094 | -0.015 |
|  | PA1463 |  | hypothetical protein | 0.381 | 0.278 | -0.27 |
|  | PA1464 |  | probable purine binding chemotaxis protein | -0.169 | 0.024 | -0.286 |
|  | PA1561 |  | aerotaxis receptor Aer | 0.896 | 0.441 | -0.51 |
|  | PA1608 |  | probable chemotaxis transducer | 0.91 | 0.232 | -0.313 |
|  | PA1646 |  | probable chemotaxis transducer | -0.61 | -0.69 | -1.5 |
|  | PA1930 |  | probable chemotaxis transducer | -0.467 | -0.153 | -0.66 |
|  | PA1946 |  | binding protein component precursor of ABC ribose transporter | -0.402 | 0.168 | 0.155 |
|  | PA2561 |  | CtpH | 1.67 | 1.28 | 0.76 |
|  | PA2573 |  | probable chemotaxis transducer | -0.61 | -0.544 | -0.17 |
|  | PA2652 |  | methyl-accepting chemotaxis protein | 0.256 | 0.61 | -0.008 |
|  | PA2654 |  | TlpQ | 1.1 | 0.247 | -0.46 |
|  | PA2920 |  | probable chemotaxis transducer | -0.105 | -0.29 | -0.15 |
|  | PA3348 |  | CherR1 | 0.536 | 0.529 | 0.322 |
|  | PA3349 |  | Probable chemotaxis protein | 0.83 | 0.535 | 0.135 |
|  | PA4290 |  | probable chemotaxis transducer | 0.77 | 0.55 | 1.47 |
|  | PA4307 |  | chemotactic transducer PctC | 0.646 | 0.937 | 0.65 |
|  | PA4309 |  | chemotactic transducer PctA | 1.02 | 0.356 | -0.5 |
|  | PA4310 |  | chemotactic transducer PctB | 0.67 | 0.455 | -0.127 |
|  | PA4496 |  | probable binding protein component of ABC transporter | -0.41 | 0.256 | -1.15 |
|  | PA4500 |  | probable binding protein component of ABC transporter | 0.076 | 0.59 | -1.42 |
|  | PA4502 |  | probable binding protein component of ABC transporter | -0.04 | 0.362 | -1.82 |
|  | PA4520 |  | probable chemotaxis transducer | 0.077 | -0.02 | 0.28 |
|  | PA4633 |  | probable chmotaxis transducer | 0.64 | 0.386 | -0.47 |
|  | PA4915 |  | probable chemotaxis transducer | -0.84 | -0.766 | 0.032 |
|  | PA4953 |  | chemotaxis protein MotB | 0.507 | 0.45 | 0.61 |
|  | PA4954 |  | chemotaxis protein MotA | 0.601 | 0.128 | 0.61 |
|  | PA5072 |  | McpK | 1.544 | 1.62 | 0.545 |
