# Supplementary material for: Distinct transcriptome and traits of freshly dispersed Pseudomonas aeruginosa cells
Source: mSphere. 2024 Nov 27;9(12):e00884-24. doi: 10.1128/msphere.00884-24 (PMC11656770; doi:10.1128/msphere.00884-24)
Supplement: Table S3 — Transcript abundance of genes linked to virulence. [file msphere.00884-24-s0005.docx]

**Supplemental Table 3. Transcript abundance of genes linked to virulence.** Fold change (log2) is relative to biofilm cells.

|  |  |  |  | **Fold change (log2)** | | |
| --- | --- | --- | --- | --- | --- | --- |
| **Category** | **Locus ID** | **Gene** | **Gene Product** | **Glutamate dispersed cells** | **Nitric oxide dispersed cells** | **Planktonic cells** |
| **LPS O-antigen (*P*. *aeruginosa)*** | PA3160 |  | O-antigen chain length regulator | 1.111664 | 0.814302 | -0.28209 |
|  | PA3159 | wbpA | UDP-N-acetyl-d-glucosamine 6-Dehydrogenase | 0.055019 | 0.454141 | -0.87217 |
|  | PA3158 | wbpB | UDP-2-acetamido-2-deoxy-d-glucuronic acid 3-dehydrogenase, WbpB | 0.369178 | 0.683725 | -0.65028 |
|  | PA3157 |  | probable acetyltransferase | -0.06254 | 0.042715 | -0.72318 |
|  | PA3156 | wbpD | UDP-2-acetamido-3-amino-2,3-dideoxy-d-glucuronic acid N-acetyltransferase, WbpD | 0.263598 | 0.733072 | 0.217859 |
|  | PA3155 | wbpE | UDP-2-acetamido-2-dideoxy-d-ribo-hex-3-uluronic acid transaminase, wbpE | 0.246533 | 0.70932 | -0.05048 |
|  | PA3154 |  | B-band O-antigen polymerase | 0.227208 | 0.254759 | -0.14617 |
|  | PA3153 |  | O-antigen translocase | 0.305691 | 0.2737 | -0.20412 |
|  | PA3152 |  | glutamine amidotransferase | 0.448362 | 0.572272 | 0.059592 |
|  | PA3151 |  | imidazoleglycerol-phosphate synthase, cyclase subunit | -0.33759 | 0.40564 | -0.30221 |
|  | PA3150 | wbpG | LPS biosynthesis protein WbpG | 0.543563 | 0.94934 | 0.593328 |
|  | PA3149 | wbpH | probable glycosyltransferase WbpH | 0.339691 | 0.703517 | -0.2214 |
|  | PA3148 | wbpI | UDP-N-acetylglucosamine 2-epimerase WbpI | 0.03437 | 0.535518 | -0.246 |
|  | PA3147 | wbpJ | probable glycosyl transferase WbpJ | -0.08978 | 0.379818 | -0.00625 |
|  | PA3146 | wbpK | probable NAD-dependent epimerase/dehydratase WbpK | -0.04673 | -0.05385 | 0.139712 |
|  | PA3145 | wbpL | glycosyltransferase WbpL | 0.42006 | 0.666427 | 0.79482 |
|  | PA3144 |  | hypothetical protein | 0.354898 | 0.335896 | -0.26396 |
|  | PA3143 |  | hypothetical protein | 0.372995 | 0.575749 | 1.089169 |
|  | PA3142 |  | hypothetical protein | -0.75667 | -0.54951 | -1.88518 |
|  | PA3141 | wbpM | nucleotide sugar epimerase/dehydratase WbpM | -0.55327 | -0.34969 | -0.71979 |
| **Phenazines biosynthesis** | PA4210 |  | probable phenazine biosynthesis protein | -0.79046 | -0.43413 | 0.036742 |
|  | PA4211 |  | probable phenazine biosynthesis protein | -0.30923 | -0.01351 | 1.862404 |
|  | PA4212 |  | phenazine biosynthesis protein PhzC | 0.328175 | 0.243664 | 0.465402 |
|  | PA4213 |  | phenazine biosynthesis protein PhzD | 0.783365 | 0.857868 | 0.325992 |
|  | PA4214 |  | phenazine biosynthesis protein PhzE | 0.204237 | 0.440478 | 0.139141 |
|  | PA4215 |  | probable phenazine biosynthesis protein | 0.20598 | 0.233845 | -0.01488 |
|  | PA4216 |  | probable pyridoxamine 5'-phosphate oxidase | 0.057547 | 0.416934 | -0.05321 |
|  | PA1899 |  | probable phenazine biosynthesis protein | -1.21398 | -0.76446 | -1.39454 |
|  | PA1900 |  | probable phenazine biosynthesis protein | -0.19677 | 0.006852 | 0.059214 |
|  | PA1901 |  | phenazine biosynthesis protein PhzC | -0.09776 | 0.028852 | 0.459541 |
|  | PA1902 |  | phenazine biosynthesis protein PhzD | 0.303791 | 0.92555 | 0.455711 |
|  | PA1903 |  | phenazine biosynthesis protein PhzE | 0.151465 | 0.353378 | 0.185134 |
|  | PA1904 |  | probable phenazine biosynthesis protein | 0.148455 | -0.0031 | -0.1751 |
|  | PA1905 |  | probable pyridoxamine 5'-phosphate oxidase | -0.72596 | 0.02451 | -0.35861 |
|  | PA4209 | phzM | probable phenazine-specific methyltransferase | -0.53904 | 0.127071 | 1.45645 |
|  | PA4217 | phzS | flavin-containing monooxygenase | -1.0656 | 0.141424 | 1.924372 |
|  | PA0051 | phzH | potential phenazine-modifying enzyme | 0.26009 | 0.607023 | 0.916728 |
| **Rhamnolipid biosynthesis** | PA3479 | rhlA | See pseudomonas.com | -0.79879 | -0.57505 | 1.307596 |
|  | PA3478 | rhlB | rhamnosyltransferase chain B | -0.59456 | -0.44778 | 1.408872 |
|  | PA1130 | rhlC | rhamnosyltransferase 2 | -0.09659 | 0.134872 | -0.77892 |
| **Pyochelin (upregulated in plk cells)** | PA4222 |  | probable ATP-binding component of ABC transporter | -0.68097 | -0.90513 | 2.255901 |
|  | PA4223 |  | probable ATP-binding component of ABC transporter | -0.4055 | -0.4133 | 2.56134 |
|  | PA4224 | pchG | pyochelin biosynthetic protein PchG | -0.57614 | -0.91578 | 2.825562 |
|  | PA4225 | pchF | pyochelin synthetase | -0.39362 | -0.84327 | 3.228566 |
|  | PA4226 | pchE | dihydroaeruginoic acid synthetase | -0.25181 | -0.84811 | 3.447293 |
|  | PA4227 | pchR | transcriptional regulator PchR | -1.61177 | -2.87568 | -0.10722 |
|  | PA4228 | pchD | pyochelin biosynthesis protein PchD | -0.42052 | -0.66298 | 3.265746 |
|  | PA4229 | pchC | pyochelin biosynthetic protein PchC | -0.83689 | -1.37737 | 2.854046 |
|  | PA4230 | pchB | salicylate biosynthesis protein PchB | -1.20077 | -0.81335 | 2.74582 |
|  | PA4231 | pchA | salicylate biosynthesis isochorismate synthase | -0.79319 | -0.63464 | 2.820703 |
| **Pyochelin receptor** | PA4221 | fptA | Fe(III)-pyochelin outer membrane receptor precursor | -1.30862 | -1.86494 | 2.453229 |
| **Pyoverdine** | PA2385 | pvdQ | See pseudomonas.com | -0.95824 | -0.89788 | -2.17031 |
|  | PA2386 | pvdA | L-ornithine N5-oxygenase | -1.63049 | -1.35502 | -1.8982 |
|  | PA2392 | pvdP | PvdP | -2.12019 | -2.25884 | -1.64852 |
|  | PA2393 |  | probable dipeptidase precursor | -1.94016 | -1.25445 | -1.96276 |
|  | PA2394 | pvdN | PvdN | -1.43639 | -1.21411 | -1.73367 |
|  | PA2395 | pvdO | PvdO | -1.41733 | -1.33919 | -1.39033 |
|  | PA2396 | pvdF | pyoverdine synthetase F | -1.75359 | -1.5444 | -2.08142 |
|  | PA2397 | pvdE | pyoverdine biosynthesis protein PvdE | -1.00723 | -0.83074 | -1.49095 |
|  | PA2399 | pvdD | pyoverdine synthetase D | -1.00712 | -1.34921 | -1.73005 |
|  | PA2400 | pvdJ | PvdJ | -1.65201 | -0.93938 | -1.88704 |
|  | PA2402 |  | probable non-ribosomal peptide synthetase | -1.2729 | -1.10455 | -1.74304 |
|  | PA2413 | pvdH | L-2,4-diaminobutyrate:2-ketoglutarate 4-aminotransferase, PvdH | -1.21239 | -1.35351 | -1.75777 |
|  | PA2424 | pvdL | PvdL | -1.18506 | -1.07886 | -1.84271 |
|  | PA2425 | pvdG | PvdG | -2.2853 | -2.25469 | -1.90249 |
|  | PA2426 | pvdS | sigma factor PvdS | -1.97419 | -1.80692 | -1.68166 |
|  | PA2427 |  | hypothetical protein | -2.33476 | -2.60946 | -1.99001 |
| **Pyoverdine receptors** | PA2398 | fpvA | ferripyoverdine receptor | -1.00712 | -1.45229 | -1.76125 |
| **Hemolytic phospholipase C** | PA0844 | plcH | hemolytic phospholipase C precursor | 0.231607 | 0.696812 | 0.853771 |
| **Non-hemolytic phospholipase C** | PA3319 | plcN | non-hemolytic phospholipase C precursor | 0.08051 | -0.37322 | 0.572979 |
| **Phospholipase C** | PA0026 | plcB | phospholipase C, PlcB | -0.5936 | -0.5611 | 0.365155 |
| **Phospholipase D** | PA3487 | pldA | phospholipase D | -0.15683 | -0.0569 | -0.19553 |
| **Alkaline protease** | PA1249 | aprA | alkaline metalloproteinase precursor | -0.83743 | -0.3472 | -0.7065 |
| **Elastase** | PA1871 | lasA | LasA protease precursor | -1.2478 | -0.90504 | 2.716839 |
|  | PA3724 | lasB | elastase LasB | -0.55576 | -0.057 | -1.14796 |
| **Protease IV** | PA4175 |  | See pseudomonas.com | -0.70644 | -0.18901 | -4.53723 |
| **Acylhomoserine lactone synthase** | PA0005 | lptA | lysophosphatidic acid acyltransferase, LptA | 0.023916 | 0.025245 | -0.86886 |
| **N-(3-oxo-dodecanoyl)-L-homoserine lactone QS system** | PA1430 | lasR | transcriptional regulator LasR | -0.87856 | -0.39707 | -0.55597 |
|  | PA1432 | lasI | autoinducer synthesis protein LasI | -0.94297 | -0.2064 | -0.28095 |
| **N-(butanoyl)-L-homoserine lactone QS system** | PA3477 | rhlR | transcriptional regulator RhlR | -0.78934 | -0.34837 | -0.35169 |
|  | PA3476 | rhlI | autoinducer synthesis protein RhlI | -0.88321 | -0.47048 | -0.79695 |
| **2-heptyl-3-hydroxy-4-quinolone QS system (upregulated in dispersed and planktonic cells cells )** | PA0996 | pqsA | probable coenzyme A ligase | 2.731543 | 2.89535 | 1.82549 |
|  | PA0997 | pqsB | PqsB | 2.687062 | 2.777572 | 2.127628 |
|  | PA0998 | pqsC | PqsC | 2.569951 | 2.694718 | 1.948579 |
|  | PA0999 | pqsD | 3-oxoacyl-[acyl-carrier-protein] synthase III | 2.127066 | 2.19257 | 1.633124 |
|  | PA1000 | pqsE | Quinolone signal response protein | 2.221171 | 2.106025 | 1.865643 |
|  | PA1001 | phnA | anthranilate synthase component I | 1.642569 | 2.01209 | 1.550547 |
|  | PA1002 | phnB | anthranilate synthase component II | -0.26318 | -0.37079 | -0.27017 |
|  | PA1003 | mvfR | See pseudomonas.com | 0.057967 | 0.38972 | -0.50569 |
| **GacS/GacA two-component system** | PA0928 | gacS | sensor/response regulator hybrid | -0.41191 | -0.01372 | -0.44789 |
|  | PA2586 | gacA | response regulator GacA | -0.46368 | -0.5296 | -0.1742 |
| ***P*. *aeruginosa* TTSS** | PA1690 | pscU | translocation protein in type III secretion | -0.47357 | -0.70423 | -2.47053 |
|  | PA1691 | pscT | translocation protein in type III secretion | -0.12355 | -0.43908 | -0.19039 |
|  | PA1692 |  | probable translocation protein in type III secretion | -1.49587 | -2.30766 | -0.46806 |
|  | PA1693 | pscR | translocation protein in type III secretion | -0.66968 | -0.54214 | 0.188059 |
|  | PA1694 | pscQ | translocation protein in type III secretion | 1.503385 | 0.746455 | 0.78582 |
|  | PA1695 | pscP | translocation protein in type III secretion | 0.172692 | -0.93115 | 0.274974 |
|  | PA1696 | pscO | translocation protein in type III secretion | 1.536459 | -0.47544 | -0.72718 |
|  | PA1697 |  | ATP synthase in type III secretion system | 0.523185 | 0.282586 | 0.225528 |
|  | PA1698 | popN | Type III secretion outer membrane protein PopN precursor | -1.33792 | -0.47864 | -1.6454 |
|  | PA1699 |  | conserved hypothetical protein in type III secretion | 2.00389 | 0.822089 | 2.36402 |
|  | PA1700 |  | conserved hypothetical protein in type III secretion | -1.48314 | 0.379481 | 1.131198 |
|  | PA1701 |  | conserved hypothetical protein in type III secretion | 3.893799 | 2.051823 | 4.615602 |
|  | PA1702 |  | conserved hypothetical protein in type III secretion | -1.05116 | -0.91988 | -0.7772 |
|  | PA1703 | pcrD | type III secretory apparatus protein PcrD | -0.43052 | -0.52027 | -1.23579 |
|  | PA1704 | pcrR | transcriptional regulator protein PcrR | -1.38689 | -1.22651 | -0.21106 |
|  | PA1705 | pcrG | regulator in type III secretion | -0.40118 | -1.30266 | -2.90315 |
|  | PA1706 | pcrV | type III secretion protein PcrV | -0.7066 | -0.69472 | -1.28393 |
|  | PA1707 | pcrH | regulatory protein PcrH | 1.453233 | 0.05029 | -0.49306 |
|  | PA1708 | popB | translocator protein PopB | -0.40456 | -0.2017 | -1.96911 |
|  | PA1709 | popD | Translocator outer membrane protein PopD precursor | -0.58597 | -0.68293 | -1.97642 |
|  | PA1710 | exsC | ExsC, exoenzyme S synthesis protein C precursor | -1.39247 | -0.9182 | -1.98541 |
|  | PA1711 | exsE | ExsE | -0.95193 | -1.38694 | -1.29766 |
|  | PA1712 | exsB | exoenzyme S synthesis protein B | -1.05235 | -1.17643 | -1.5804 |
|  | PA1713 | exsA | transcriptional regulator ExsA | -1.72759 | -1.96722 | -3.22925 |
|  | PA1714 | exsD | ExsD | -0.6797 | -0.74703 | -1.1234 |
|  | PA1715 | pscB | type III export apparatus protein | 0.362148 | -1.12927 | -2.2246 |
|  | PA1716 | pscC | Type III secretion outer membrane protein PscC precursor | 0.724097 | -0.34591 | -0.09586 |
|  | PA1717 | pscD | type III export protein PscD | -0.20944 | -0.8738 | -0.47056 |
|  | PA1718 | pscE | type III export protein PscE | 1.925203 | 2.00729 | 2.294099 |
|  | PA1719 | pscF | type III export protein PscF | 0.266269 | -1.2924 | -0.79313 |
|  | PA1720 | pscG | type III export protein PscG | 0.108266 | -1.12791 | 0.313926 |
|  | PA1721 | pscH | type III export protein PscH | -0.32061 | -0.01997 | -1.1629 |
|  | PA1722 | pscI | type III export protein PscI | 0.143835 | -0.2043 | 0.711947 |
|  | PA1723 | pscJ | type III export protein PscJ | -0.66322 | -0.94764 | -0.80165 |
|  | PA1724 | pscK | type III export protein PscK | 0.454979 | 0.060082 | 0.857099 |
|  | PA1725 | pscL | type III export protein PscL | 0.090303 | -1.49107 | -0.55282 |
| ***P*. *aeruginosa* TTSS translocated effectors** | PA3841 | exoS | exoenzyme S | -0.07538 | -0.29405 | -0.04875 |
|  | PA0044 | exoT | exoenzyme T | -0.18585 | -0.58341 | -0.21997 |
|  | PA2191 | exoY | adenylate cyclase ExoY | -0.87287 | -0.40942 | -0.82461 |
| **Exototoxin-A (ETA)** | PA1148 | toxA | exotoxin A precursor | -1.56104 | -1.38997 | -3.49412 |
| **Hydrogen cyanide production** | PA2193 | hcnA | hydrogen cyanide synthase HcnA | 2.098934 | 2.3871 | 2.23564 |
|  | PA2194 | hcnB | hydrogen cyanide synthase HcnB | 0.640149 | 0.688967 | 0.670584 |
|  | PA2195 | hcnC | hydrogen cyanide synthase HcnC | 0.228893 | 0.036738 | 0.594564 |
| **Xcp type II secretion system** | PA3095 | xcpZ | general secretion pathway protein M | -0.24625 | 0.181663 | 0.934331 |
|  | PA3096 | xcpY | general secretion pathway protein L | -0.85976 | -0.65488 | -0.5128 |
|  | PA3097 | xcpX | general secretion pathway protein K | -1.02201 | -0.31514 | -0.78337 |
|  | PA3098 | xcpW | general secretion pathway protein J | -0.65539 | 0.085091 | -0.68343 |
|  | PA3099 | xcpV | general secretion pathway protein I | -0.08404 | 0.047134 | -0.21456 |
|  | PA3100 | xcpU | General secretion pathway outer membrane protein H precursor | -0.53797 | -0.13211 | -0.55981 |
|  | PA3101 | xcpT | general secretion pathway protein G | -0.34946 | -0.04813 | -0.63462 |
|  | PA3102 | xcpS | general secretion pathway protein F | -0.54214 | -0.33695 | -0.73124 |
|  | PA3103 | xcpR | general secretion pathway protein E | -0.48643 | -0.16917 | 0.006895 |
|  | PA3104 | xcpP | secretion protein XcpP | -0.77433 | -1.12952 | -0.86678 |
|  | PA3105 | xcpQ | general secretion pathway protein D | -0.59454 | -0.34495 | -0.49654 |
| **Hxc type II secretion system** | PA0677 |  | HxcW putative pseudopilin | 1.292354 | 0.045349 | -0.73154 |
|  | PA0678 |  | HxcU putative pseudopilin | -0.99724 | -0.59929 | -0.78484 |
|  | PA0679 |  | hypothetical protein | -0.63565 | -1.80913 | 0.096568 |
|  | PA0680 |  | HxcV putative pseudopilin | -1.01862 | 0.400085 | -3.23904 |
|  | PA0681 |  | HxcT pseudopilin | -1.10392 | 0.724067 | 1.176029 |
|  | PA0682 |  | HxcX atypical pseudopilin | 2.092681 | 0.138907 | 2.777842 |
|  | PA0683 |  | probable type II secretion system protein | 2.093051 | 1.346327 | 2.324767 |
|  | PA0684 |  | probable type II secretion system protein | -0.87538 | 0.015509 | 0.173982 |
|  | PA0685 |  | probable type II secretion system protein | 0.324677 | 0.055506 | 1.095953 |
|  | PA0686 |  | probable type II secretion system protein | 1.42973 | 0.059386 | 1.845038 |
|  | PA0687 |  | probable type II secretion system protein | 0.429927 | 1.124632 | 0.508054 |
