# Supplementary material for: Distinct transcriptome and traits of freshly dispersed Pseudomonas aeruginosa cells
Source: mSphere. 2024 Nov 27;9(12):e00884-24. doi: 10.1128/msphere.00884-24 (PMC11656770; doi:10.1128/msphere.00884-24)
Supplement: Table S4 — Differential expression of genes encoding c-di-GMP modulating enzymes. [file msphere.00884-24-s0006.docx]

**Supplemental Table 4. Differential expression of genes encoding c-di-GMP modulating enzymes in dispersed and planktonic cells relative to biofilm cells.** Fold change (log2) is relative to biofilm cells.

|  |  |  |  |  | **Fold change (log2)** | | |
| --- | --- | --- | --- | --- | --- | --- | --- |
| **Predicted enzyme activity ^a^** | **Locus ID** | **Domain** ^b^ | **Mutant biofilm** ^c^ | **other biofilm related functions** | **Planktonic cells** | **Glutamate dispersed cells** | **Nitric oxide dispersed cells** |
| DGC | PA4396 | GGDEF | None |  | -0.46089 | -0.88097 | -0.461939 |
|  | PA0169 (siaD) | GGDEF | reduced |  | -0.278305 | -0.623044 | -0.625578 |
|  | PA0290 | GGDEF | reduced |  | -1.979293 | -1.211072 | -1.146693 |
|  | PA0338 | GGDEF | reduced |  | -0.455355 | 0.118554 | -0.202279 |
|  | PA0847 | GGDEF | None |  | 0.687795 | 0.342425 | 0.347414 |
|  | PA1107 (roeA) | GGDEF | reduced | Arginine-induced | -0.448579 | 0.323126 | 0.345144 |
|  | PA1120 (yfiN) | GGDEF | reduced |  | 0.431414 | 0.052987 | -0.189253 |
|  | PA1433 | GGDEF-EAL |  |  | -1.172028 | -0.411326 | -0.613289 |
|  | PA1851 | GGDEF | None |  | 0.053367 | -0.04134 | 0.199022 |
|  | PA2870 | GGDEF | reduced |  | -0.200781 | 0.106169 | 0.067774 |
|  | PA3177 | GGDEF | reduced | stress response, drug tolerance | -0.126725 | 0.530032 | 0.512511 |
|  | PA3343 (hsbd) | GGDEF | None | HptB pathway | 0.38024 | 0.507019 | 0.706568 |
|  | PA3702 (wspR) | GGDEF | reduced | surface contact sensing | -0.659733 | -0.71155 | -0.647556 |
|  | PA4332 (sadC) | GGDEF | reduced | surface contact sensing (Pil-Chp), SadC/BifA, arginine-induced | -0.694197 | 0.125869 | -0.161901 |
|  | PA4843 (gcbA) | GGDEF | reduced | attachment, dispersion, AmrZ reglulated | 1.560196 | 1.456049 | 1.043771 |
|  | PA4929 (nicD) | GGDEF | None | dispersion | 1.039968 | 1.348215 | 1.124534 |
|  | PA5487 | GGDEF | reduced |  | -0.354492 | -0.101724 | -0.237055 |
| DGC+PDE | PA1727 (mucR) | GGDEF-EAL | none (PAO1), reduced (PA14) | dispersion | 1.341815 | 0.403916 | 0.052717 |
| PDE | PA0575 (rmcA) | GGDEF-EAL | reduced ** | biofilm maintenance | 0.405242 | 0.667144 | 0.658196 |
|  | PA0861 (rbdA) | GGDEF-EAL | None | Dispersion | -0.467205 | 0.080482 | 0.035683 |
|  | PA2133 | EAL | None |  | 3.347089 | 0.581097 | 2.943365 |
|  | PA2200 | EAL | None |  | -0.697807 | -0.819574 | -0.704035 |
|  | PA2572 (FimX) | HD-GYP | reduced ** |  | -0.353842 | 0.159491 | 0.17958 |
|  | PA3311 (nbdA) | GGDEF-EAL |  | NO-induced dispersion | -0.851851 | 0.220032 | -0.110821 |
|  | PA3825 | EAL | None |  | -0.581472 | -0.205995 | -0.746946 |
|  | PA3947 (rocR) | EAL | None |  | 1.437244 | -0.804932 | -0.186111 |
|  | PA4108 | HD-GYP | None |  | -0.921272 | -0.595346 | -0.645499 |
|  | PA4367 (bifA) | GGDEF-EAL | hyperbiofilm | SadC/BifA, attachment | -0.503755 | -0.274155 | -0.333634 |
|  | PA4601 (morA) | GGDEF-EAL | reduced ** | biofilm maintenance | 0.351973 | 0.863721 | 0.817228 |
|  | PA4781 | HD-GYP | reduced ** |  | -0.450539 | -0.211269 | -0.383493 |
|  | PA4959 (fimX) | GGDEF-EAL |  |  | -1.195918 | -0.583601 | -0.66033 |
|  | PA5017 (dipA) | GGDEF-EAL | hyperbiofilm | dispersion | -0.284488 | 0.455192 | 0.525111 |
|  | PA5295 (proE) | GGDEF-EAL |  |  | -0.805565 | 0.30495 | -0.110076 |
| GGDEF-EAL | PA0285 (pipA) | GGDEF-EAL |  |  | 0.782809 | 0.28534 | 0.548619 |
|  | PA1181 | GGDEF-EAL |  |  | 0.039438 | 0.137952 | -0.100019 |
|  | PA2072 | GGDEF-EAL |  |  | -0.22977 | -0.122376 | -0.273611 |
|  | PA2567 | GGDEF-EAL |  |  | 0.836207 | 1.437009 | 1.697091 |
|  | PA3258 | GGDEF-EAL |  |  | 0.756666 | -0.02211 | 0.229685 |
|  | PA5442 | GGDEF-EAL |  |  | 0.951716 | -0.314249 | -0.126544 |

^**,^ observed reduction of biofilms not in accordance with current model on the role of c-di-GMP in biofilm formation

^a^, enzyme activity based on (1) and others, see source

^b^, domain assignment based on (2)

^c^, evaluation of the mutant biofilm architecture relative to the parental PA14 stain, based on (2)

1. **Kulesekara H, Lee V, Brencic A, Liberati N, Urbach J, Miyata S, Lee DG, Neely AN, Hyodo M, Hayakawa Y, Ausubel FM, Lory S.** 2006. Analysis of *Pseudomonas aeruginosa* diguanylate cyclases and phosphodiesterases reveals a role for bis-(3'-5')-cyclic-GMP in virulence. Proceedings of the National Academy of Sciences **103:**2839-2844.

2. **Ha D-G, Richman ME, O'Toole GA.** 2014. Deletion mutant library for investigation of functional outputs of cyclic diguanylate metabolism in *Pseudomonas aeruginosa* PA14. Applied and Environmental Microbiology **80:**3384-3393.

3. **Kazmierczak BI, Lebron MB, Murray TS.** 2006. Analysis of FimX, a phosphodiesterase that governs twitching motility in *Pseudomonas aeruginosa*. Molecular microbiology **60:**1026-1043.

4. **Navarro MV, De N, Bae N, Wang Q, Sondermann H.** 2009. Structural analysis of the GGDEF-EAL domain-containing c-di-GMP receptor FimX. Structure **17:**1104-1116.
