# Supplementary material for: Distinct transcriptome and traits of freshly dispersed Pseudomonas aeruginosa cells
Source: mSphere. 2024 Nov 27;9(12):e00884-24. doi: 10.1128/msphere.00884-24 (PMC11656770; doi:10.1128/msphere.00884-24)
Supplement: Table S5 — Primers. [file msphere.00884-24-s0007.docx]

| **Gene name** | **Forward Primer (5’-3’)** | **Reverse Primer (5’-3’)** |
| --- | --- | --- |
| *vqsR* | CTCCGAAGATTTCGAAACGA | GGCATAGGGTTCTTTCACCA |
| *rhlA* | GCGCGATGGCGACCAC | CACCACCGAGCTGCGG |
| *pscL* | CGCGACTACCAGGACTACCT | CGCTTCTGCTCCTGGTAAAC |
| *piv* | GTCAACCGTCCCTACTGGAG | CGAGTCGGCGAAATACGATA |
| *exsA* | TCCATGAATAGCTGCAGACG | ATCGAGGAGTTGCTGATGCT |
| *pcrV* | GAGCCGCTGAGAAAATCCTT | GACCCCACGCTATATGGCTA |
| *phzB* | CCAAAGGCCAGGATCGCCTG | CTGCGGGACCGGAATGCTC |
| *hcn* | TGAACGTCAACACGATATCCA | CATTGAGCACGTTGAGCAC |
| *argR* | ATCGCTTGCCGAGGAAGC | GAACAGCGGCTCGTAGAG |
| *rpoS* | GCATCATGCTGGACGAGT | GCGTGTAGTCGATGTGCT |

**Supplementary Table 5.** Primers used in this study.
